# Dehydrative π-extension to nanographenes with zig-zag edges

**DOI:** 10.1038/s41467-018-07095-z

**Published:** 2018-11-12

**Authors:** Dominik Lungerich, Olena Papaianina, Mikhail Feofanov, Jia Liu, Mirunalini Devarajulu, Sergey I. Troyanov, Sabine Maier, Konstantin Amsharov

**Affiliations:** 1 Department of Chemistry and Pharmacy, Organic Chemistry II, Friedrich-Alexander-University Erlangen-Nuernberg, Nikolaus-Fiebiger-Str. 10, 91058 Erlangen, Germany; 2 Department of Chemistry & Molecular Technology Innovation Presidential Endowed Chair, University of Tokyo, 7-3-1 Hongo, Bunkyo-ku, Tokyo, 113-0033 Japan; 3 Department of Physics, Friedrich-Alexander-University Erlangen-Nuernberg, Erwin-Rommel-Str. 1, 91058 Erlangen, Germany; 4 Chemistry Department, Moscow State University, Leninskie Gory, Moscow Russia 119991

## Abstract

Zig-zag nanographenes are promising candidates for the applications in organic electronics due to the electronic properties induced by their periphery. However, the synthetic access to these compounds remains virtually unexplored. There is a lack in efficient and mild strategies origins in the reduced stability, increased reactivity, and low solubility of these compounds. Herein we report a facile access to pristine zig-zag nanographenes, utilizing an acid-promoted intramolecular reductive cyclization of arylaldehydes, and demonstrate a three-step route to nanographenes constituted of angularly fused tetracenes or pentacenes. The mild conditions are scalable to gram quantities and give insoluble nanostructures in close to quantitative yields. The strategy allows the synthesis of elusive low bandgap nanographenes, with values as low as 1.62 eV. Compared to their linear homologues, the structures have an increased stability in the solid-state, even though computational analyses show distinct diradical character. The structures were confirmed by X–ray diffraction or scanning tunneling microscopy.

## Abstract (web summary)

Nanographenes with zig-zag peripheries are expected to have unique electronic properties, but their application in organic electronics has been curbed by their difficult synthesis. Here, the authors develop a facile route to zig-zag nanographenes based on a key dehydrative π-extension reaction.

## Introduction

**Fig. 1:**
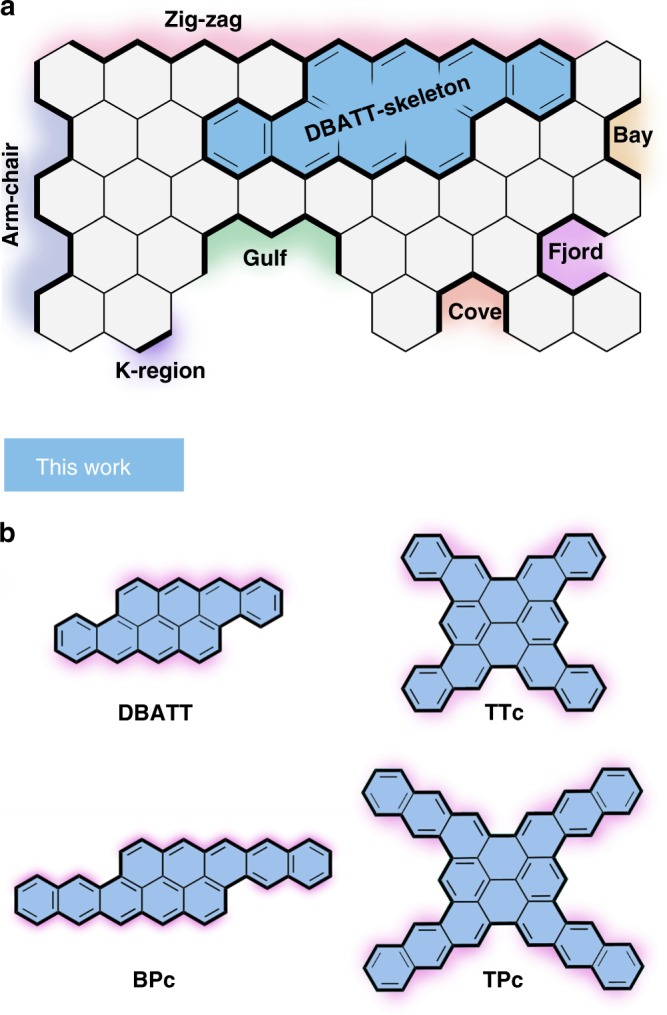
Relevant terminologies and overview of this work. **a** Nomenclature and schematic depiction of relevant edge structures; **b** Herein enabled zig-zag NGs by the DPEX reaction

The chemistry of polycyclic aromatic hydrocarbons (PAHs), essentially founded by E. Clar in the middle of the 20th century^1^, is nowadays flourishing more than ever in form of structurally precise carbon nanostructures^2,3^. Driven by the vast potential in next generation technologies^4–6^, and accelerated by the continuous development of synthetic methodologies^7–12^, the field of PAHs and nanographenes (NGs) remains intensively studied by researchers from the physical sciences. The high interest origins in the fact that the physical and chemical properties of the hydrocarbons are directly related to their size, shape, and especially edge topology (Fig. 1a)^13^. In that respect, PAHs with zig-zag periphery are especially interesting, because they show typically higher charge carrier mobilities than compounds with an armchair periphery. Further, they exhibit strong absorptions in the visible region as a result of a decreased highest occupied molecular orbital–lowest unoccupied molecular orbital gap (HOMO-LUMO gap; HLG)^14,15^. As a consequence, significant contributions from open-shell resonances in the ground state are frequently observed^16^. Keywords that highlight acene-type PAHs with zig-zag periphery range from singlet fission^17–21^, auspicious charge carrier mobilities^22,23^, to amplified spontaneous emission^24^, and enlarge the interest in PAHs by the fields of spintronics and molecular magnetism^25-27^. Recently, various research groups reported on substituted angularly fused-tetracenes^28–32^, which overcome the low stability of linear acenes, by increasing the intrinsic number of Clar’s-sextets^33^. These fused structures showed excellent performances in field-effect transistors^28,29,31,32^. Among these slipped bis-tetracenes, a significant focus was set on derivatives of the 2.3,8.9-dibenzanthanthrene (**DBATT**) aromatic carbon skeleton. Earlier, **DBATT** was already revealed as a probe for the Shpol’skii effect^34–36^, it was used as single molecule optical transistor^37^, or as solid-state single-quantum emitter^38^. Interestingly, even though this molecule was known since the early explorations of E. Clar in the 1940s, to date its structure has been assigned only by chemical rational and UV/Vis spectroscopy^39,40^. However, repetition of Clar’s route in our own laboratories yielded **DBATT** only in trace amounts and required intensive purification procedures.

Herein, we report on the preparation and investigation of π-extended molecules based on the **DBATT** carbon skeleton. We developed a facile three-step synthesis, which utilizes an acid-promoted reductive intramolecular cyclization of aromatic aldehydes – a dehydrative π-extension (DPEX) reaction – as key transformation. Our DPEX protocol allows the synthesis of **DBATT** in highly pure form in close to quantitative yield. Importantly, DPEX is suitable for the preparation of pristine zig-zag edges, without the need of bulky or electronically stabilizing substituents, which preserves and allows to study the true nature of the zig-zag nanographenes (zzNGs). Finally, the power of DPEX is demonstrated by the preparation of the π-extended and unprecedented homologues, fused tetrakis-tetracene (**TTc**), slipped bis-pentacene (**BPc**), and fused tetrakis-pentacene (**TPc**); pushing the boundaries of the bottom-up preparation of zzNGs (Fig. 1b).

## Results

### Synthesis of nanographenes by DPEX

**Fig. 2:**
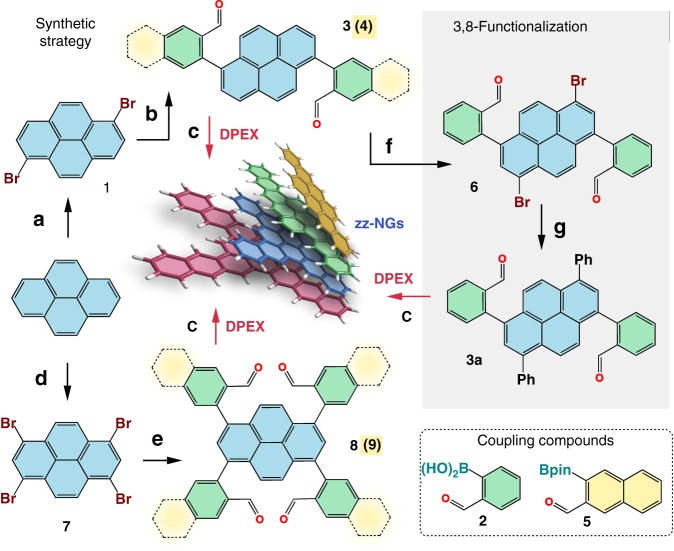
Synthesis of zig-zag NGs. **a** CHCl_3_, Br_2_; **b** 2:1 toluene/MeOH, 2.5% Pd(PPh_3_)_4_, K_2_CO_3_, 80 °C, N_2_ (3: 61%, 4: 65%); **c** DPEX*:* CH_2_Cl_2_, 2 vol% sat. SnCl_2_•2H_2_O/*i*-PrOH, 1 vol% conc. H_2_SO_4_, rt, (quant.); **d** nitrobenzene, Br_2_; **e** 2:1 toluene/MeOH, 4% Pd(PPh_3_)_4_, K_2_CO_3_, 80 °C, N_2_ (8: 81 %, 9: 91 %); **f** CH_2_Cl_2_, 1.6 equiv. HMTAB, rt (quant.); **g** 2:1 toluene/MeOH, 2.5 % Pd(PPh_3_)_4_, K_2_CO_3_, 80 °C, N_2_ (**3a**: 66 % with phenylboronic acid); HMTAB: 1:2 hexamethylentetramine-bromine complex

In the case of **DBATT** and its derivatives, pyrene was brominated with bromine in chloroform, yielding 1,6-dibromopyrene **1** on a decagram scale, which was purified by a single recrystallization step from xylenes (see Supplementary Methods)^41,42^. Two-fold Suzuki-Miyaura coupling of **1** with 2-formylphenylboronic acid **2** gave the diarylated pyrene precursors, 1,6-bis(2-formylphenyl)pyrene **3**, which was obtained in pure form and good yields after silica gel plug filtration and precipitation with hexanes from dichloromethane. The preparation of **BPc**-precursor **4**, was achieved analogously by the coupling with 3-formylnaphthalene-2-boronic acid pinacol ester **5**, which was obtained in three steps from 1,2,4,5-tetrabromobenzene (see Supplementary Methods). As proof of principle, regioselective 3,8-functionalization of **3** could be easily achieved by bromination to **6** which can be either directly condensed to dibromo DBATT (**bBr-DBATT**) via DPEX (see Supplementary Methods) or converted to the respective aryl derivative by Suzuki-Miyaura coupling with e.g., phenylboronic acid to **3a**, yielding respective diaryl **DBATT**s (see also compound **16** and pyridinyl substituted compound **S5**). This route avoids the earlier reported formation of non-selective 1,2 and 1,4-Michael addition products^30,43^. For small scale brominations, the 1:2 hexamethylentetramine-bromine complex (HMTAB) was found to be particularly useful, however not mandatory for the selective bromination^44^. Starting from 1,3,6,8-tetrabromopyrene **7**, four-fold-substituted pyrenes **8** and **9** were obtained in equal manner. An overview on the synthetic scheme is shown in Fig. 2. Precursors **3**, **3a**, **4**, **8**, and **9** were subjected to the elaborated DPEX conditions (vide infra, see also Supplementary Table 1), giving the target NGs in close to quantitative yield, without any sign of side-product formation, as determined by HPLC and MS. Supplementary Table 2 shows all synthesized precursors and Supplementary Table 3 shows all nanographenes accessed by DPEX (**DBATT**, **bPh-DBATT**, **bBr-DBATT**, **TTc**, **BPc**, **TPc**, **11**, **13**, **17**, and **S7**). Even though DPEX takes place already in neat H_2_SO_4_ in moderate yields (see Supplementary Table 1), the best possible conditions were first refined by the conversion of **3** to **DBATT** on an analytical scale. The reaction outcome was followed by quantitative HPLC analysis.

**3** and the other nanographene precursors show relatively good solubility in CH_2_Cl_2_ and THF compared to other tested solvents like e.g., hexanes, toluene, and ethyl acetate, which subsequently determined the tested reaction media for the DPEX reaction. However, only CH_2_Cl_2_ as solvent provides the best reaction outcome. As reducing agent, we focused on common SnCl_2_•2H_2_O. The addition of 2 vol% of a saturated solution of SnCl_2_•2H_2_O, dissolved in iso-propanol is superior over all other investigated reduction systems. Interestingly, using methanol instead, which dissolves SnCl_2_•2H_2_O by far better than iso-propanol, the conversion appears to be faster, however the reaction is accompanied by the formation of unidentified side products. Although the side products are formed in trace amounts, as indicated by HPLC analysis, further post synthetic purification appears to be difficult due to the low solubility. On the other hand, the addition of iso-propanol significantly slows down the conversion but remarkably improves the selectivity and the reaction outcome; used as mere solvent however, the reaction is inhibited completely. The addition of 1 vol% conc. H_2_SO_4_ initiates the reaction, which is indicated (in case of **3** and **3a**) by a rapid purple coloration of the mixture, accompanied by the formation of a white precipitate. Supplementary Fig. 1 shows a pictured illustration of the single reaction steps. Hereby, the empiric ratio of 2:1 of the SnCl_2_/*i-*PrOH–solution and the H_2_SO_4_ plays a crucial role for a successful outcome. After work-up with aqueous hydrochloric acid and extraction, the product is precipitated with MeOH, which gives pure **DBATT** in close to quantitative yields. The usage of stronger acids like e.g., trifluoromethanesulfonic acid (TfOH), or 34% oleum shows good performances, however significantly worse than conc. H_2_SO_4_. On the other hand, weaker acids like e.g., trifluoroacetic acid (HTFA) and acetic acid, show no conversion at all. As mentioned above, reactions carried out in neat H_2_SO_4_ and thus lacking a reducing agent were found to give **DBATT** in moderate yields. Since the required two-electron reduction process cannot stem from H_2_SO_4_, we surmise a disproportionation reaction between two intermediate molecules, leading to **DBATT** and oxidized derivatives. This assumption is further supported by the fact that the yields of **DBATT** decreased upon lowering the concentration of **3** and never exceeded yields of 50%. Further details can be extracted from Supplementary Table 1.

**Fig. 3:**
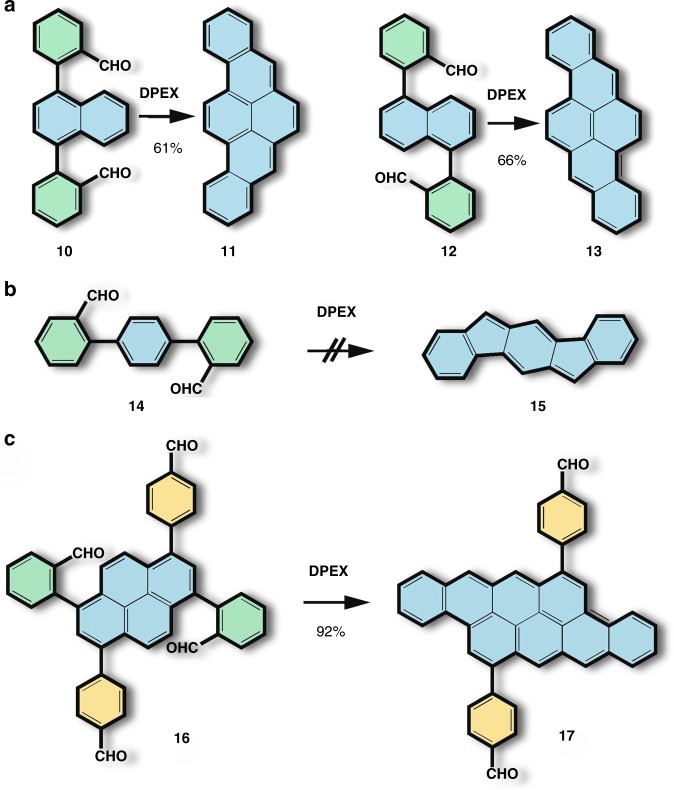
The scope of the DPEX protocol. **a** Reaction with less reactive naphthalene core units; **b** No formation of five-membered rings; **c** Tolerance towards misplaced formyl groups

With respect to the scope of the DPEX protocol, the reactivity is demonstrated using the less reactive naphthalene core as the central aromatic unit. The respective precursor molecules **10** and **12** are obtained from 1,4-dibromonaphthalene and 1,5-diiodonaphthalene by standard two-fold Suzuki-Miyaura coupling reactions (see Supplementary Methods). Despite the lower activity of the naphthalene core, the DPEX cyclization results the desired benzo[*rst*]pentaphene **11** and dibenzo[*b,def*]chrysene **13** in moderate yields (Fig. 3a). Interestingly, in both cases the cyclisation is only successful in the presence of SnCl_2_, indicating that SnCl_2_ plays a crucial role in the DPEX process and participates already in the first reaction step. Thus, the mechanism of DPEX appears to be more complex than the intuitively assumed two-step domino reaction. This is also supported by the lack in formation of undesired pentagons under DPEX conditions, which otherwise would be expected for protonated forms of aldehydes. This transformation however, is completely suppressed as demonstrated by the attempt to synthesize indeno[1,2-*b*]fluorene **15** from *p*-terphenyl dicarbaldehyde **14** (Fig. 3b). Surprisingly, compound **14** remains completely intact under DPEX condition, pointing out that the aldehyde functionality tolerates the reaction conditions if being misplaced. In other words, the aldehyde group shows activity only if it is placed in the formal cove region of the PAH. In order to support further this claim, the para-formylphenyl DBATT precursor **16**, bearing two aldehyde groups in active, and two in inactive positions was prepared, starting from dibromo precursor **6**. As shown in Fig. 3c, **16** reacts under typical DPEX conditions selectively, yielding the desired **DBATT** derivative **17** in 92% isolated yield. This unprecedentedly high regioselectivity of DPEX provides essential flexibility in design and facile access to complex functional PAHs. Regarding other functionalities, DPEX shows to be tolerant towards keto-groups, which undergo no transformation (compare compound **S6** in the Supplementary Methods); heterocycles like pyridine substituted precursors do not affect the outcome of DPEX and nearly quantitative conversions are obtained (see compound **S7** in the Supplementary Methods).

Noteworthy, the solvents were neither degassed, pre-distilled or purified, and used as obtained from the suppliers, which underpins the applicability of this protocol. Moreover, the reactions were performed under ambient atmosphere at room temperature. The only pre-caution taken was the avoidance of direct light irradiation, since **DBATT** is known to undergo light-induced oxidative decomposition^39^. In order to proof that this reductive-condensation protocol is suitable for the preparative–scale production, we carried out the reaction on a 0.50 g scale of **3**, which allowed us to isolate **DBATT** in 0.44 g as pure dark blue solid (isolated yield 96%). Supplementary Fig. 1 shows a detailed and pictured illustration of the single reaction steps. Accordingly, **TTc** was prepared on a 100 mg scale and **BPc** and **TPc** on a 20 mg scale.

### Structure elucidation

**Fig. 4:**
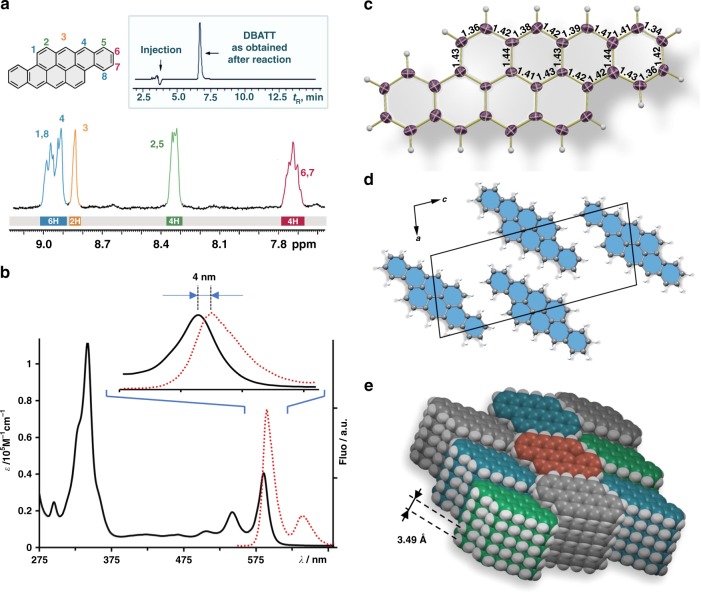
Spectroscopic analysis and structure elucidation of **DBATT**. **a** ^1^H NMR (*o-*DCB-D_4_, 100 °C, 400 MHz); proton assignments were correlated with computed NMR spectra at the DFT RB3LYP 6–311 + G(d, p) GIAO level of theory; inset shows HPLC chromatogram after reaction work-up; **b** absorption (black) and emission (red) in THF at rt; **c** single crystal X-ray structure depicted as ORTEP model with 50% thermal ellipsoids, independent C–C bond lengths are indicated; **d** View onto the (101) face of the crystal (lattice) structure; depicted as balls and sticks model; **e** columnar crystal packing motif of **DBATT** with an interlayer distance of 3.49 Å; depicted as space filling model

Structure analysis of highly insoluble pristine NGs is evolving as major problem in modern nanographene sciences. Therefore, solubilizing groups are typically attached to the PAH’s skeleton, leading inevitably to an alteration of its original characteristics^28–30,32^. Spectroscopically at the border of solubility for NMR analysis, we elucidated the ^1^H NMR spectrum of **DBATT**, obtained at 100 °C in *o*-DCB (Fig. 4a). The peaks were assigned by the correlation with its computed NMR spectrum (RB3LYP 6–311 + G(d,p) GIAO). The absorption and emission spectra of **DBATT** are shown in Fig. 4b. The lowest energy absorption maximum is found at *λ*_max_ = 586 nm; it is absorption onset of *λ*_onset_ = 600 nm corresponds to the HOMO–LUMO transition of 2.07 eV and compares well to its computed HLG (vide infra). The small Stoke’s shift of 4 nm gives an emission maximum at 600 nm and is characteristic for the rigid carbon skeleton. For the irrefutable structure determination, we took advantage of the stability of our zzNGs towards thermal sublimation. In case of **DBATT**, we were able to grow crystals in shape of dark blue needles (see Supplementary Fig. 2) by sublimation at 310 °C at 10^–5^ mbar, suitable for single crystal X-ray diffraction (see Fig. 4c). Unlike the examples from substituted **DBATT** structures^28–30,32^, the aromatic skeleton of pristine **DBATT** remains flat. However, the lack of substituents becomes most apparent in view of the crystal packing. While e.g., threefold substituted triisopropylsilylethynyl-**DBATT**^30^, packs in a pseudo-sandwich herringbone motif with a π–π distance of 3.61 Å, pristine **DBATT** assembles in slipped co-facially aligned columnar stacks with an interlayer distance of 3.49 Å (Fig. 4d, e). This particular arrangement is especially favorable for energy efficient exciton splitting processes, known as singlet fission, as the eclipsed conformation maximizes the frontier molecular orbital overlap of the HOMO and the LUMO^45,46^.

**Fig. 5:**
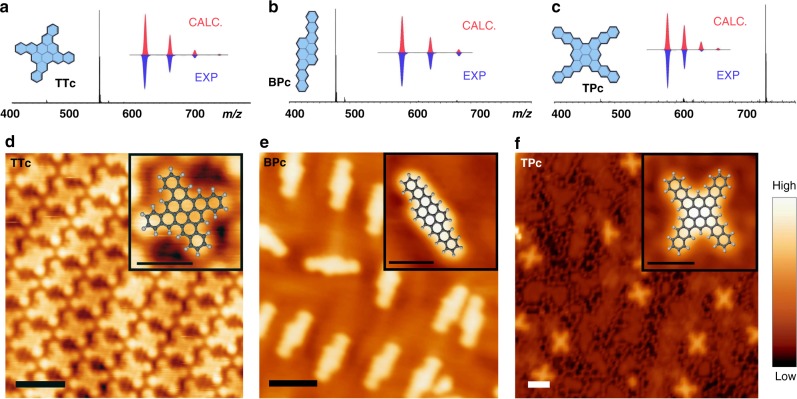
Structure elucidation of larger NGs – **TTc**, **BPc** and **TPc**. **a**–**c** LDI-MS, insets show the calculated and measured isotope pattern, respectively; **d**–**f** STM images of **TTc** on Au(111) at 77 K, **BPc** on Au(111) at 4.7 K and **TPc** on Ag(111) at 4.7 K. The perfect fit of the superimposed DFT models corroborates the unambiguous identification of the NGs. The **TPc**’s are surrounded by bright protrusions that are assigned to halogens, which are residues from the synthesis (see SI). Scale bars: 2 nm and 1 nm for the insets with the DFT model overlaid images, respectively. Tunneling conditions: 100 pA/1 V, 50 pA/-500 mV, and 100 pA/-500 mV

With 10, 12, and 16 annulated benzene rings respectively, the actual highlight-NGs – **TTc, BPc** and **TPc** – could not be brought into solution without decomposition (boiling 1,2,4-trichlorobenzene). In that respect, laser desorption ionization mass spectrometry, as shown in Fig. 5a–c, indicate the high selectivity and full completeness of the DPEX process. No starting material, intermediates and other side products can be detected by LDI-MS which gave the first evidence for the constitutional integrity of the NGs. In the case of **BPc** a small intensity signal corresponding to the oxygen adduct can be detected, indicating slow oxidation of the compound under ambient conditions (no special precautions were taken during the MS preparation and analysis).

The structural proof was unambiguously obtained by low-temperature scanning tunneling microscopy (STM). The NGs were sublimed in ultra-high vacuum between 300 and 395 °C onto a Au(111) or Ag(111) surface, which was kept at room temperature. The STM results shown in Fig. 5d–f reveal that the size and shape of the NGs fit perfectly to the superimposed structural models, which were obtained by DFT. **BPc** and **TPc** adsorb as single molecules on Au(111) and Ag(111) respectively, while **TTc** forms a self-assembly on Au(111) (detailed analysis of the self-assembly is discussed in the Supplementary Discussion and depicted in Supplementary Fig. 14). This is the first report on the successful preparation and characterization of such extended zig-zag nanographenes.

### Computations

**Fig. 6:**
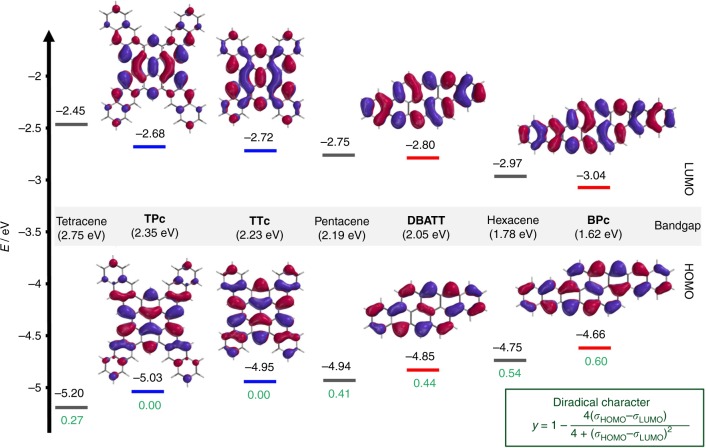
Computational analysis of the zzNGs. Calculated HOMO-LUMO energies and frontier orbitals (isoval: 0.02 a.u.) at the DFT B3LYP 6–311 G + (d,p) level of theory; Theoretical diradical character *y* (green digits) was obtained from the broken symmetry calculation at the UHF 6–31 + G(d,p) level of theory

In order to shed light into the electronic properties of the NGs, we carried out DFT calculations. We determined the HOMO–LUMO levels at the B3LYP-6–311 + G(d,p) level of theory; the theoretical diradical character *y* (*y* = 0 pure closed-shell; *y* = 1 pure open-shell) was determined according to the equation in the inset in Fig. 6^47^, from the occupation number of the frontier molecular orbitals (*σ*_HOMO_, *σ*_LUMO_), at the UHF 6–31 + G(d,p) level of theory^48,49^. The values were compared to the parent linear acenes (tetracene, pentacene, and hexacene) and are depicted in Fig. 6. With respect to the HOMO–LUMO gap the cross-shaped NGs **TTc** and **TPc** allocate values of 2.23 eV and 2.35 eV between tetracene and pentacene, respectively. At the first glance counterintuitive, **TPc** reveals a larger gap than **TTc**. However, this can be attributed to the stabilizing effect of Clar’s sextets in the outer benzene rings, which are more dominant in **TPc** and thus lower the HOMO level due to an increased aromatic stabilization energy. Interestingly, from broken symmetry calculations, no mixing of the highest occupied natural orbital (HONO) and lowest unoccupied natural orbital (LUNO), and therefore no diradical character (*y* = 0) for **TTc** and **TPc** can be observed. Even though the bandgap of tetracene is bigger than of the respective fourfold fused NGs, it shows already a diradical character of *y* = 0.27. Unlike the cross-shaped NGs, the bis-fused compounds **DBATT** and **BPc** show, with an increasing number of annulated benzene rings, a decreasing HOMO–LUMO gap (HLG). With a computed gap of 2.05 eV, which is in good agreement with its absorption spectrum, **DBATT** can be located between pentacene and hexacene; with *y* = 0.44 it shows a slightly higher diradical character than pentacene. However, in the solid-state **DBATT** shows to be kinetically much more stable than pentacene, which tends to undergo [2 + 2] cycloaddition reactions rapidly and requires typically stabilizing groups like e.g., triisopropylsilylacetylene. With a gap of only 1.62 eV, which corresponds to a theoretical transition at 765 nm, **BPc** shows the smallest HLG of the herein discussed NGs and compares well to notoriously unstable heptacene^50^. Even though a significant diradical character of *y* = 0.60 is computed for **BPc**, its persistence during the synthesis, thermal sublimation conditions, and storage in the solid state is remarkable.

## Discussion

The recent progress in nanographene chemistry benefitted to a large extend from synthetic methodologies that allow the design of carbon nanostructures, such as nanographenes (NGs) and graphene nanoribbons, in high structural precision. While the edge-topology displays a strong lever in order to tune the electronic properties of NGs, major achievements towards zzNGs have been achieved only by on-surface synthesis under UHV conditions^14^, or utilizing solubilizing and protective side groups^51,52^. Wet chemically, little synthetic efforts have been made to synthesize pristine zig-zag peripheries. As demonstrated in this study, we developed a facile reaction protocol that allows for the introduction of peripheral zig-zag-methine units into nanographene molecules. The key-step – the dehydrative π-extension (DPEX) – shows to be, due to its mild conditions, highly efficient in the preparation of nanographenes with small HOMO–LUMO gaps and significant diradical character. Furthermore, we show that the dehydrative cycloaromatization of aldehydes can be realized very effectively; utilizing readily available chemicals, we circumvent the usage of precious transition metals, or otherwise uncommon reagents in the final stage of the synthesis. DPEX showed to be expandable in any direction – larger acenes with low bandgaps, and multiple C=C bond formations in one molecule, yielding NGs with up to sixteen annulated benzene rings in close to quantitative yield. Its selectivity towards misplaced aldehydes, or ketones, its toleration of halogens, as well as heterocycles like pyridine, allows for a sophisticated compound design. The tolerance of the reaction towards moisture and air, the scalability to gram-quantities, and the extraordinary performance towards the preparation of highly insoluble NGs, makes this methodology a powerful and versatile instrument in the chemists’ synthetic toolbox. Thus, we truly believe that alongside the recent synthetic development of synthetic methodologies towards NGs^7–12^, DPEX will substantially contribute to the blooming field of carbon-based nano-architectures in physical sciences.

## Methods

### Synthesis of 2,3,8,9-dibenzanthanthrene **DBATT** (typical DPEX procedure)

A 250 mL round bottom flask equipped with a magnetic stir bar was charged under ambient atmosphere with precursor **3** (20.0 mg, 48.7 µmol) and dissolved in CH_2_Cl_2_ (100 mL). While stirring, a solution of SnCl_2_•2H_2_O (500 mg, 2.22 mmol) in *i-*PrOH (2.0 mL) was added, followed by the addition of conc. H_2_SO_4_ (1.0 mL). The mixture was stirred at rt with protection from daylight for 18 h. The dark purple mixture was quenched by vigorous shaking with 1 *M* HCl (2.0 mL). The mixture was diluted with CH_2_Cl_2_ (20 mL) and washed with H_2_O (1 × 50 mL). The aqueous layer was extracted with CH_2_Cl_2_ (3 × 20 mL) including the dark insoluble solids (product). The combined organics were diluted with MeOH (100 mL) and the CH_2_Cl_2_ was removed on the rotary evaporator at atmospheric pressure at 50 °C. The formed precipitate in the MeOH layer was centrifuged, the MeOH layer was decanted and the solid was washed with MeOH. The product was dried in vacuo and obtained in 98% yield (18.0 mg, 47.8 µmol) as dark blue solid. ^1^H NMR (400 MHz, *o*-DCB-D_4_, 373 K): *δ* (ppm) = 9.02–8.88 (6 H, m), 8.84 (2 H, m), 8.38–8.29 (4 H, m), 7.79–7.67 (4 H, m); HRMS (MALDI; DCTB matrix) (*m/z*) calculated for C_30_H_16_ (M^+^) 376.1247, found 376.1764; EA calculated: C 95.72, H 4.28, found: C 94.88, H 4.54; further details are given in the Supplementary Discussion and Methods.

## Electronic supplementary material

Supplementary Information

## Data Availability

The data that support the findings of this study are available from the corresponding author upon reasonable request. CCDC 1835109 (**DBATT**) contains the Supplementary crystallographic data for this paper. These data can be obtained free of charge from The Cambridge Crystallographic Data Centre via www.ccdc.cam.ac.uk/data_request/cif.
