# Supplementary material for: Dehydrative π-extension to nanographenes with zig-zag edges
Source: Nat Commun. 2018 Nov 12;9:4756. doi: 10.1038/s41467-018-07095-z (PMC6232111; doi:10.1038/s41467-018-07095-z)
Supplement: Supplementary file 1 — Supplementary Information [file 41467_2018_7095_MOESM1_ESM.pdf]

Supplementary Information

Dehydrative π-extension to nanographenes with zig-zag edges

Dominik Lungerich, Olena Papaianina, Mikhail Feofanov, Jia Liu, Mirunalini Devarajulu, Sergey I. Troyanov, Sabine Maier, Konstantin Amsharov*

Supplementary Methods

General Information

All chemicals were purchased from Sigma-Aldrich, TCI, or ChemPUR and used without any further purification.

Reactions that require an inert atmosphere were degassed by three cycles of sonication under membrane-pump vacuum, followed by the exchange of the atmosphere with the inert gas (Ar or N_2). All reactions were carried out in darkness.

Thin layer chromatography (TLC) was performed on Merck silica gel 60 F254, and visualized by UV-light (254 nm, 366 nm). Silica gel plug filtration was performed on Macherey–Nagel silica gel 60 M (230–400 mesh, 0.04–0.063 mm).

HPLC analysis was carried out, using an analytical Cosmosil PBr (4.6 x 250 mm) column (Shimadzu, photo-diode array detector SPD-M20A). HPLC solvents were purchased from VWR or Sigma Aldrich.

NMR spectra were recorded on a Bruker Avance 400 or Jeol EX 400, operating at 400 MHz (^{1}H NMR) and 100 MHz (^{13}C NMR) and Bruker Avance 300 MHz (^{1}H NMR) and 75 MHz (^{13}C NMR). Deuterated solvents were purchased from Sigma Aldrich and used as received. Signals were referenced to residual solvent peaks (δ in parts per million (ppm) $CDCl_3$: ^{1}H 7.24 ppm, ^{13}C 77.0 ppm; CD_2Cl_2: ^{1}H 5.32 ppm, ^{13}C 53.5 ppm. *o*-DCB-D_4: ^{1}H 6.93 and 7.19 ppm, DMSO: ^{1}H 2.49 ppm. The resonance multiplicities are indicated as “s“ (singlet), “d” (doublet), “t” (triplet), and “m” (multiplet). Signals referred to as bs (broad singlet) are not clearly resolved or significantly broadened.

APPI mass spectra were recorded on a quadrupole time-of-flight (QqToF) mass spectrometer, the Bruker MAXIS. (MA)LDI-TOF mass spectra were recorded with a Shimadzu Biotech Axima Confidence and a Bruker Reflex III.

UV-vis spectra were recorded on a Varian Cary 5000 UV-vis spectrophotometer at rt.

Fluorescence spectra were recorded on a Shimadzu RF–5301PC spectrofuorophotometer at rt.

Computations were performed with the Gaussian ’09 package.[1]

Supplementary Table 1. Selected reaction conditions for *DPEX* of 3 to DBATT; carried out in CH_2Cl_2 at room temperature under air in darkness; work-up: washing with 1M HCl and extraction with CH_2Cl_2; the extracts were diluted to 100 mL and subjected to HPLC analysis; denoted percentage values for reagents refer to vol%; a) determined by HPLC; b) carried out in *i*-PrOH as solvent, c) HPLC profile of **DBATT** as obtained after reaction (entry 5), (5PBr column, 4:1 toluene/MeOH, 1mL/min, 35 °C, detection 580 nm).

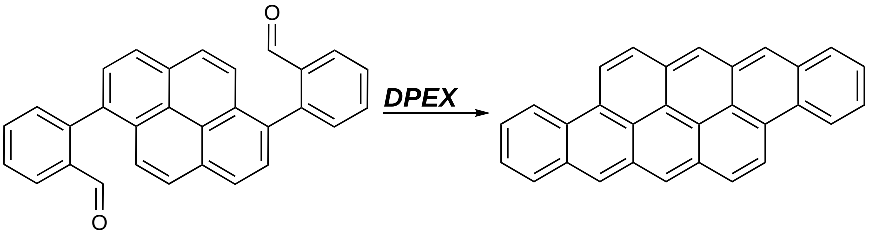

N	*Reducing Agent*	*Acid*	*Time*	*Yield* [a]
1	2.5 % sat. $SnCl_2 \cdot H_2O$/MeOH	5.0 % TfOH	1 h	42 %
2	2.5 % sat. $SnCl_2 \cdot H_2O$/MeOH	5.0 % oleum	1 h	49 %
3	2.5 % sat. $SnCl_2 \cdot H_2O$/MeOH	5.0 % H_2SO_4	1 h	60 %
4	2.5 % sat. $SnCl_2 \cdot H_2O$/MeOH	2.5 % H_2SO_4	12 h	94 %
**5**	**2.0 % sat. $SnCl_2 \cdot H_2O$/*i*-PrOH**	**1.0 % H_2SO_4**	**18 h**	**99 %**
6	--	5.0 % H_2SO_4	1 h	33 %
7	--	neat H_2SO_4	20 min	34 %
8[b]	$SnCl_2 \cdot H_2O$	5.0 % H_2SO_4	18 h	0 %

c)

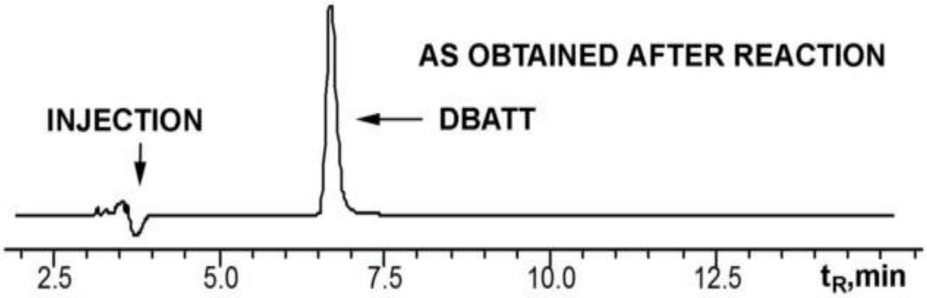

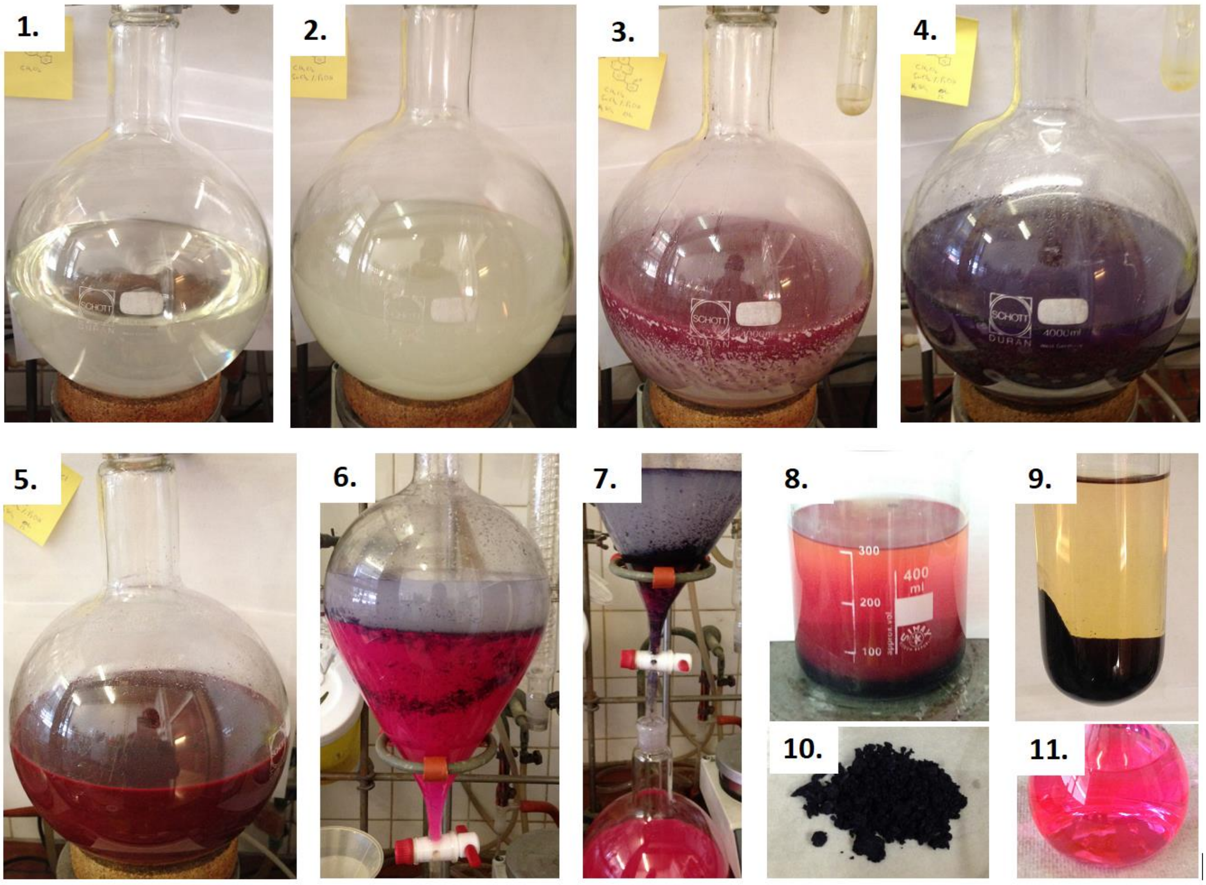

Supplementary Figure 1. Pictured description of the synthesis of **DBATT**: 1. **3** dissolved in CH_2Cl_2; 2. After addition of $SnCl_2{\cdot}2H_2O$/*i*-PrOH solution; 3. After 10 min of addition of H_2SO_4; 4. After stirring for 18 h; 5. After quenching with 1M HCl; 6. Washing/extraction; 7. Collecting of dark product at the interface of the organic and aqueous layer; 8. After precipitation with MeOH; 9. Centrifugation; 10. Dried solid; 11. **DBATT** in solution (10^{-5} M THF).

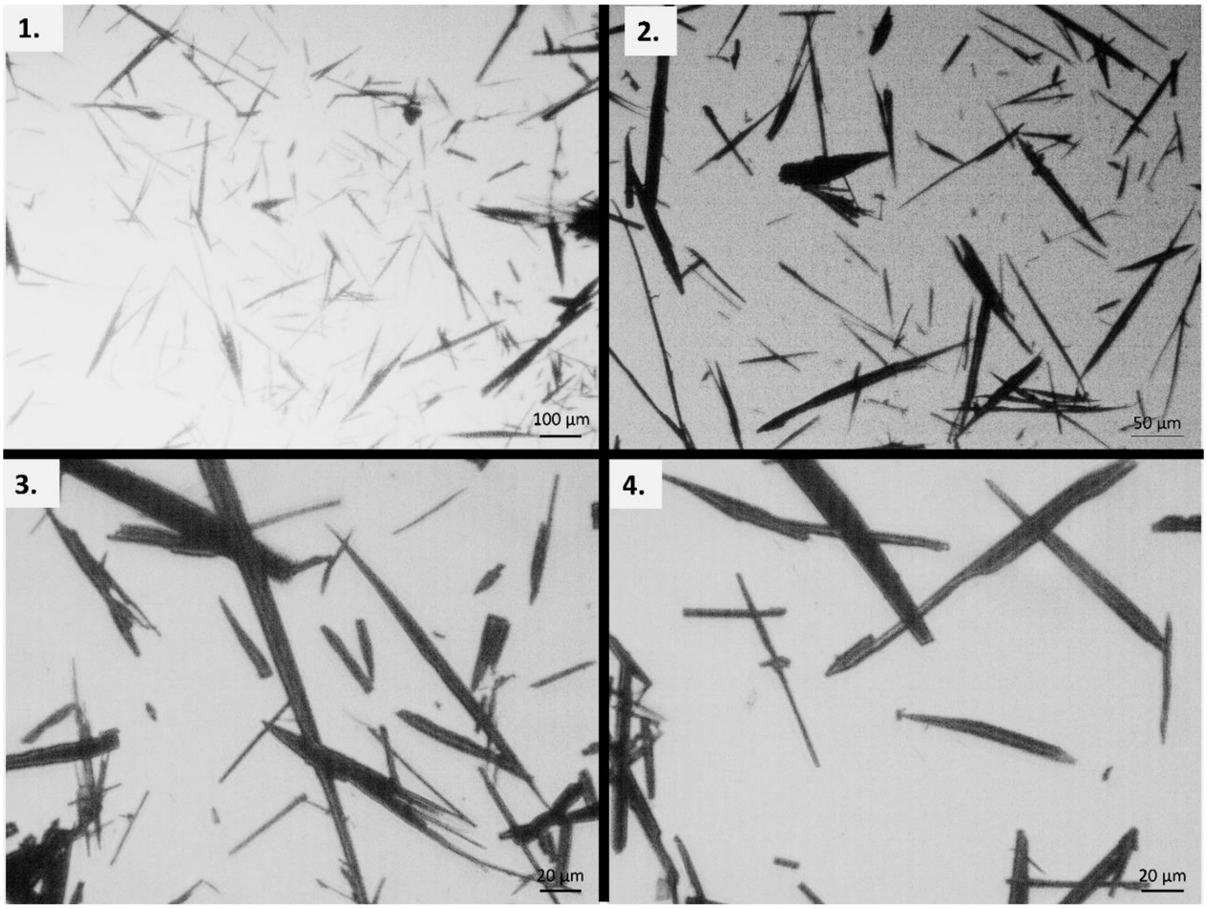

Supplementary Figure S2. Pictures (b/w) of sublimed (310 °C, 10^{-5} mbar) crystals of **DBATT** at different magnifications.

Experimental procedures and characterization data

HMTAB complex

according to the procedure from: M. M. Heravi, N. Abdolhosseini, H. A. Oskooie, *Tettrahedron Lett.* **2005**, *46*, 8959–8963.

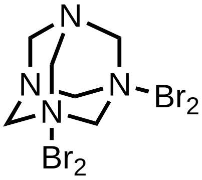

$C_6H_{12}Br_4N_4$
M = 459,81

A 250 mL round bottom flask, was charged with hexamethylenetetramine (8.50 g, 60.0 mmol) and dissolved in $CHCl_3$ (100 mL). A solution of Br_2 (20.0 g, 125 mmol) in $CHCl_3$ (100 mL) was added dropwise at rt. A yellow solid precipitated. The mixture was stirred for an additional 30 min, and the yellow solid was collected by vacuum filtration, yielding the desired complex in 92 % (25.5 g, 55.2 mmol). The complex was used as obtained without further characterization.

1,6-Dibromopyrene (1)

modified procedure from: B. Kim, Park Youngil, Lee J. *J. Mater. Chem C*, **2013**, *1*, 432-440.

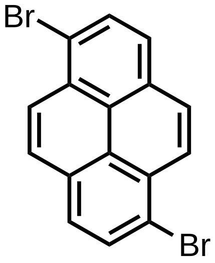

$C_{16}H_8Br_2$
M = 360,05

A 5 L two-neck round bottom flask, equipped with a magnetic stir bar and a dropping funnel was charged with pyrene (30.0 g, 148 mmol) and dissolved in $CHCl_3$ (1.5 L). A solution of Br_2 (15.3 mL, 297 mmol) in $CHCl_3$ (500 mL) was added dropwise at rt over 8 h. The formed precipitate was collected after 16 h, washed with MeOH and purified by recrystallization from hot xylene (mixture of isomers), yielding the product as beige solid in 36 % (19.0 g, 52.8 mmol).

NMR data of pure product were in accordance with literature.

2,3-Dibromonaphthalene (S1)

modified procedure from: A. Link, C. Fischer, C. Sparr, *Angew. Chem. Int. Ed.* **2015**, *54 (11)*, 12163–12166.

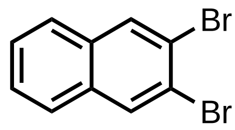

$C_{10}H_6Br_2$
M = 285,97

A dry 250 mL Schlenk round bottom flask, equipped with a magnetic stir bar and a dropping funnel was charged with 1,2,4,5-tetrabromobenzene (3.74 g, 10.0 mmol) and dissolved in toluene (100 mL) and furan (5 mL) under a N_2-atmosphere. The mixture was cooled and a temperature between -20 °C to -30 °C was maintained while adding a mixture of 2.5 M *n*-BuLi/hexanes (4.0 mL; 11.0 mmol) dissolved in 100 mL hexanes over 3 h. The mixture was allowed to warm to rt over a period of 12 h. The white suspension was quenched with MeOH (1.0 mL). The clear solution was washed with H_2O (2x 20 mL) and

the aqueous layer was extracted with Et_2O (1x 10 mL). The combined organics were dried over $MgSO_4$, concentrated to give an oil, which upon precipitation from CH_2Cl_2 and hexanes yielded dibromoepoxynaphthalene as white solid. The intermediate product was used without further characterization in the next step.

A dry 500 mL Schlenk round bottom flask equipped with a magnetic stir bar and a condenser was charged with Zn-powder (6.00 g, 92.0 mmol) and suspended in THF (150 mL) at 0 °C. $TiCl_4$ (6.0 mL, 55.0 mmol) was added slowly via syringe at 0 °C, and the mixture was brought to reflux for 5 min until all yellow solids dissolved. The mixture was cooled down to 0 °C again. A solution of the prepared epoxydibromonaphthalene in THF (60 mL) was added slowly over 10 min to the Zn/Ti mixture. The mixture was heated to 80 °C for 20 h. The black mixture was cooled to 0 °C and quenched slowly by the dropwise addition of ice cold aqueous HCl solution (40 mL conc. HCl, 110 mL ice/H_2O). The dark purple solution was extracted with CH_2Cl_2 (3x 75 mL), the combined organics were washed with H_2O (1x 100 mL) and dried over $MgSO_4$. The crude was purified by flash liquid chromatography (SiO_2; hexanes), yielding the product as white solid in 47 % (1.34 g, 4.68 mmol).

^{1}H NMR (300 MHz, $CDCl_3$, 293 K): δ= 8.11 (2H, s); 7.73-7.67 (2H, m); 7.52-7.45 (2H, m).
^{13}C NMR (75 MHz, $CDCl_3$, 293 K): δ= 133.0; 132.2; 127.2; 126.8; 122.0.

2-Bromo-3-formylnaphthalene (S2)

modified procedure from: L. S. Chen, G. J. Chen, C. Tamborski, *J. Organomet. Chem.* **1980**, *193*, 283–292.

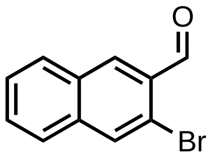

$C_{11}H_7BrO$
M = 235,08

A dry 250 mL Schlenk round bottom flask, equipped with a magnetic stir bar was charged with 2,3-dibromonaphthalene **S1** (1.00 g, 3.50 mmol) and dissolved in 1:1 THF/Et_2O (115 mL) under N_2 atmosphere. The solution was cooled to -110 °C (EtOH/liquid N_2) and 2.5 M *n*-BuLi (1.4 mL, 3.5 mmol) was added slowly over 5 min. The pale yellow solution was stirred at -110 °C for 30 min. DMF (5 mL) was added dropwise over 5 min. The mixture was gradually warmed to -80 °C over a period of 2 h. The mixture was quenched with a 1:3 conc. HCl/EtOH mixture (20 mL) and let warm to -60 °C. The mixture was poured to a 1:5 conc. HCl/EtOH mixture (120 mL) at rt. The layers were separated and the aqueous layer was extracted with Et_2O (3x 50 mL). The combined organics were dried over $MgSO_4$ and purified by plug filtration (SiO_2, 4x6 cm; hexanes to 1:1 hexanes/CH_2Cl_2), yielding the product as white solid in 92 % (760 mg; 3.23 mmol).

R_f (SiO_2): 0.49 (1:1 hexanes/CH_2Cl_2).
^{1}H NMR (300 MHz, $CDCl_3$, 293 K): δ= 10.48 (1H, s); 8.43 (1H, s); 8.09 (1H, s); 7.94 (1H, m); 7.77 (1H, m); 7.62 (1H, ddd, *J*=1.40, 6.84, 8.18 Hz); 7.55 (1H, ddd, *J*=1.34, 6.83, 8.10 Hz).
^{13}C NMR (75 MHz, $CDCl_3$, 293 K): δ= 191.9; 136.8; 132.5; 131.8; 131.5; 130.5; 130.0; 129.8; 127.4; 126.9; 120.5.

3-Formylnaphthalene-2-boronic acid pinacol ester (5)

modified procedure from: Y.Y. Jhang, T. T. Fan-Chiang, J. M. Huang, J. C. Hsieh, *Org. Lett.* **2016**, *18*, 1154–1157.

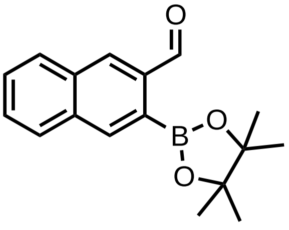

$C_{17}H_{19}BO_3$
M = 282,15

A 20 mL septum vial, equipped with a magnetic stir bar was charged with 2-bromo-3-formylnaphthalene **S2** (500 mg, 2.13 mmol), bis(pinacolato)diboron (650 mg, 2.56 mmol), KOAc (627 mg, 6.39 mmol) $Pd(dppf)Cl_2{\cdot}CH_2Cl_2$ (52 mg, 63.9 µmol) and dissolved in 1,4-dioxane (8 mL). The mixture was degassed and stirred at 80 °C for 24 h under N_2 atmosphere. The mixture was diluted with CH_2Cl_2 (25 mL) and washed with a saturated solution of aqueous $NaHCO_3$ (15 mL). The organic layer was dried over $MgSO_4$ and purified by plug filtration (SiO_2 4x6 cm; 1:1 hexanes/CH_2Cl_2 to CH_2Cl_2), yielding a yellow oil which slowly solidifies to an off-white solid, yielding the product in 43 % (260 mg, 921 µmol).

R_f (SiO_2): 0.26 (1:1 hexanes/CH_2Cl_2).
^{1}H NMR (300 MHz, $CDCl_3$, 293 K): δ = 10.56 (1H, s), 8.41 (1H, s); 8.28 (1H, s), 7.95 (1H, m); 7.89 (1H, m), 7.63–7.53 (2H, m); 1.43 (s, 12H).
^{13}C NMR (75 MHz, $CDCl_3$, 293 K): δ = 194.4; 137.6; 136.6; 135.2; 133.4; 130.8; 129.6; 128.8; 128.3; 127.7; 84.3; 24.9.

1,5-diiodonaphthalene (S3)

modified procedure from J. Gonzalo Rodríguez, J. Luis Tejedor**,** *J. Org. Chem.* **2002**, *67*, 7631–7640.

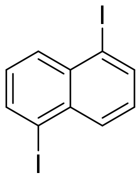

$C_{10}H_6I_2$
M = 379.97

To a solution of sodium nitrite (1.80 g, 44.0 mmol) in concentrated sulfuric acid (15 mL) at 0 °C was added dropwise a solution of 1,5-diaminonaphthalene (1.80 g, 19.0 mmol) in glacial acetic acid (15 mL). The mixture was stirred for 15 min and poured onto ice (50 g), and then a solution of potassium iodide (60.0 g, 0.60 mol) in water (60 mL) was added and stirred overnight. The solid was filtered, dried, and extracted with dichloromethane. The solvent was evaporated under reduced pressure and residue was purified by silica gel column chromatography, eluting with isohexane yielding 1,5-diiodonaphtalene as yellow solid in 47% (2.00 g, 5.26 mmol).

R_f (SiO_2): 0.70 (isohexane).

^{1}H NMR (400 MHz, $CDCl_3$, 293 K): δ 8.14 (d, *J* = 2.6 Hz, 2H), 8.12 (s, 2H), 7.25 (t, *J* = 7.9 Hz, 2H).

^{13}C NMR (101 MHz, $CDCl_3$, 293 K) δ 138.5, 134.6, 133.6, 128.4, 99.7.

Phenyl(2-(4,4,5,5-tetramethyl-1,3,2-dioxaborolan-2-yl)phenyl)methanone (S4)

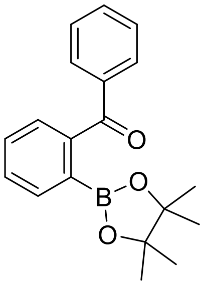

$C_{19}H_{21}BO_3$
M = 308.18

A 100 mL round bottom flask, equipped with a magnetic stir bar was charged with (2-bromophenyl)(phenyl)methanone (1.00 g, 3.78 mmol), bis(pinacolato)diboron (1.16 g, 4.57 mmol), KOAc (1.10 g, 11.21 mmol) $Pd(dppf)Cl_2$ (140 mg, 191 µmol) and dissolved in 1,4-dioxane (25 mL). The mixture was degassed and stirred at 80 °C for 24 h under N_2 atmosphere. The mixture was diluted with CH_2Cl_2 (50 mL) and washed with water (30 mL). The organic layer was dried over $MgSO_4$ and purified by by silica gel column chromatography, eluting with isohexane- isohexane:EtOAc (4:1), yielding Phenyl(2-(4,4,5,5-tetramethyl-1,3,2-dioxaborolan-2-yl)phenyl)methanone as yellow oil which slowly solidifies to an off-white solid in 51 % (510 mg, 1.65 mmol)

R_f (SiO_2): 0.24 (4:1 isohexanes/EtOAc).

^{1}H NMR (400 MHz, $CDCl_3$, 293 K): δ 7.80 – 7.76 (m, 1H), 7.74 (dd, *J* = 7.6, 1.2 Hz, 1H), 7.57 – 7.41 (m, 6H), 1.18 (s, 12H)

^{13}C NMR (101 MHz, $CDCl_3$, 293 K): δ 198.1, 143.7, 138.1, 133.8, 132.3, 130.3, 130.0, 129.7, 128.8, 128.1, 83.9, 24.5.

Synthesis of *DPEX*-precursors

1,6-Bis(2-formylphenyl)pyrene (3)

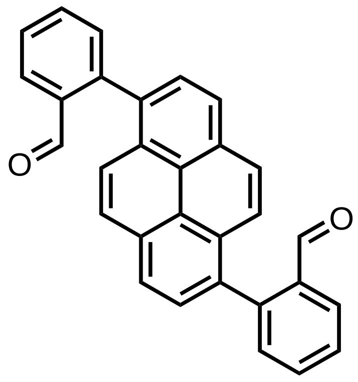

$C_{30}H_{18}O_2$
M = 410,47

A 100 mL round bottom flask equipped with a magnetic stir bar and a condenser was charged with 1,6-dibromopyrene **1** (1.00 g, 2.78 mmol), 2-formylphenylboronic acid **2** (1.00 g, 6.67 mmol), K_2CO_3 (2.00 g, 14.5 mmol) and $Pd(PPh_3)_4$ (80.0 mg, 69.0 µmol). The solids were suspended in 2:1 toluene/MeOH (30 mL), degassed and the atmosphere was exchanged by argon. The mixture was brought to reflux for 16 h. The mixture was diluted with toluene (100 mL) and washed with H_2O (2x 50 mL). The aqueous layer was extracted with toluene (1x 50 mL) and the combined organics were dried over $MgSO_4$. The solvent was evaporated and the product was precipitated from CH_2Cl_2/hexanes. The solids were filtered, washed with hexanes and dried in vacuo, yielding the product as pale yellow solid in 61 % (700 mg, 1.71 mmol).

^{1}H NMR (400 MHz, $CDCl_3$, 293 K): δ = 9.66 (1H, s); 9.65 (1H, s), 8.24 (2H, d, *J*=7.80 Hz); 8.18 (2H, d, *J*=7.84 Hz), 8.07 (2H, d, *J*=9.24 Hz), 7.97 (2H, d, *J*=7.76 Hz), 7.82 (2H, dd, *J*=2.04, 9.20 Hz), 7.77 (2H, m), 7.65 (2H, dd, *J*=7.56, 7.56 Hz), 7.57 (2H, d, *J*=7.52 Hz).

^{13}C NMR (75 MHz, $CDCl_3$, 293 K): δ = 191.83; 191.79; 144.52; 144.48; 135.10; 135.06; 133.7; 133.6; 133.5; 132.20; 132.16; 131.0; 130.3; 128.7; 128.4; 128.3; 127.5; 127.4; 125.3; 124.7; 124.60; 124.58.

HRMS (APPI; toluene): Chemical Formula: $C_{30}H_{18}O_2$ calc. 410.1301, found 410.1308.

1,6-bis(2-formylphenyl)-3,8-diphenylpyrene (3a)

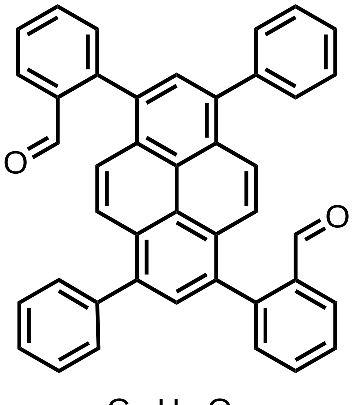

$C_{42}H_{26}O_2$
M = 562,67

A 5 mL septum vial equipped with a magnetic stir bar was charged with 1,6-dibromo-3,8-bis(2-formylphenyl)pyrene **6** (100 mg, 176 µmol), phenylboronic acid (54 mg, 440 µmol), K_2CO_3 (73.0 mg, 528 µmol) and $Pd(PPh_3)_4$ (10.0 mg, 8.80 µmol). The solids were suspended in 2:1 toluene/MeOH (3.0 mL), degassed and the atmosphere was exchanged by argon. The mixture was heated to 80 °C for 18 h. The mixture was diluted with CH_2Cl_2 (25 mL) and washed with H_2O (2x 10 mL). The aqueous layer was extracted with CH_2Cl_2 (1x 10 mL) and the combined organics were dried over $MgSO_4$. The mixture was purified by plug filtration (SiO_2, 2x4 cm; CH_2Cl_2) and the product

was precipitated from CH_2Cl_2/hexanes. The solids were filtered, washed with hexanes and dried in vacuo, yielding the product as yellow solid in 66 % (66 mg, 116 µmol).

^{1}H NMR (400 MHz, $CDCl_3$, 293 K): δ = 9.74 (2H, s); 8.15 (2H, d, *J*=7.80 Hz); 8.19 (2H, d, *J*=9.48 Hz); 7.98 (2H, s); 7.79 (1H, dd, *J*=2.84, 9.48 Hz); 7.77-7.71 (2H, m); 7.66-7.58 (8H, m); 7.54-7.48 (4H, m); 7.47-7.42 (2H, m).

^{13}C NMR (100 MHz, $CDCl_3$, 293 K): δ = 192.0; 191.9; 144.40; 144.35; 140.3; 137.67; 137.66; 135.02; 134.98; 133.74; 133.65; 132.9; 132.23; 132.21; 130.6; 130.29; 130.27; 129.51; 129.49; 128.7; 128.5; 128.4; 127.6; 127.39; 127.35; 126.3; 125.30; 125.27; 125.26.

HRMS (APPI; toluene): Chemical Formula: $C_{42}H_{26}O_2$ calc. 562.1927, found 562.1926.

1,6-Bis(3-formylnaphthyl)pyrene (4)

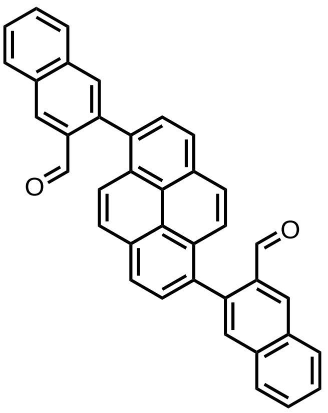

$C_{38}H_{22}O_2$
M = 510,59

A 5 mL septum vial equipped with a magnetic stir bar was charged with 1,6-dibromopyrene **1** (70.0 mg, 193 µmol), 2-formylnaphthyl-3-boronic acid pinacol ester **5** (120 mg, 425 µmol), K_2CO_3 (176 mg, 1.28 mmol) and $Pd(PPh_3)_4$ (11.0 mg, 9.7 µmol). The solids were suspended in 2:1 toluene/MeOH (3.0 mL), degassed and the atmosphere was exchanged by argon. The mixture was heated to 110 °C for 20 h. The mixture was diluted with CH_2Cl_2 (20 mL) and washed with H_2O (2x 10 mL). The aqueous layer was extracted with CH_2Cl_2 (1x 10 mL) and the combined organics were dried over $MgSO_4$. The solvent was evaporated and the product was precipitated from CH_2Cl_2/hexanes. The solids were filtered, washed with hexanes and dried in vacuo, yielding the product as yellow solid in 76 % (75 mg, 147 µmol).

^{1}H NMR (400 MHz, DMSO, 293 K): δ = 9.77 (2H, s); 8.79 (2H, s); 8.34 (2H, d, *J*=7.88 Hz); 8.41 (2H, d, *J*=7.84 Hz); 8.24-8.13 (8H, m); 7.83-7.72 (6H, m).

^{13}C NMR (125 MHz, CD_2Cl_2, 293 K): δ = 192.5; 192.4; 139.6; 136.3; 135.7; 135.6; 134.8; 133.74; 133.70; 132.9; 132.04; 131.98; 131.5; 131.0; 130.6; 130.5; 130.04; 130.02; 129.4; 128.8; 128.59; 128.57; 127.9; 125.9; 125.4; 125.1.

HRMS (APPI; MeCN, toluene): Chemical Formula: $C_{38}H_{22}O_2$ calc. 510.1614, found 510.1614.

1,6-dibromo-3,8-bis(2-formylphenyl)pyrene (6)

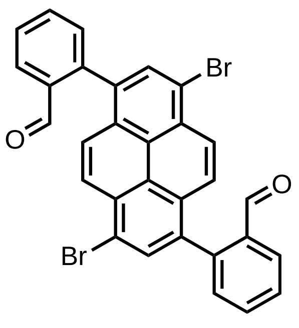

$C_{30}H_{16}Br_2O_2$
M = 568,26

A 100 mL round bottom flask equipped with a magnetic stir bar was charged with 1,6-bis(2-formylphenyl)pyrene **3** (250 mg, 610 µmol) and dissolved in CH_2Cl_2 (50 mL). 1:2 Hexamethylenetetramine-bromine complex (459 mg, 1.00 mmol) was added as solid and the mixture was stirred in darkness at rt for 48 h. The yellow suspension was washed with a 3 % aqueous $NaHCO_3$ solution (1x 150 mL), H_2O (1x 50 mL) and dried over $MgSO_4$. The pale yellow solution was concentrated and precipitated by addition of MeOH, yielding the product as pale yellow solid in 87 % (302 mg ,531 µmol).

^{1}H NMR (400 MHz, CD_2Cl_2, 293 K): δ = 9.66 (1H, s), 9.65 (1H, s); 8.48 (2H, d, *J*=9.48 Hz); 8.32 (2H, s); 8.17 (2H, d, *J*=7.60 Hz), 7.92-7.79 (4H, m), 7.73 (2H, t, *J*=7.34 Hz); 7.60 (2H, d, *J*=7.91 Hz).

^{13}C NMR (100 MHz, CD_2Cl_2, 293 K): δ = 191.20; 191.17; 142.4; 142.3; 135.2; 135.04; 135.00; 134.0; 133.9; 133.0; 132.21; 132.15; 129.8; 129.6; 129.1; 128.1; 127.0; 126.8; 125.3; 120.6.

HRMS (APPI; toluene): Chemical Formula: $C_{30}H_{16}Br_2O_2$ calc. 565.9512, found 565.9509.

1,3,6,8-Tetrakis(2-formylphenyl)pyrene (8)

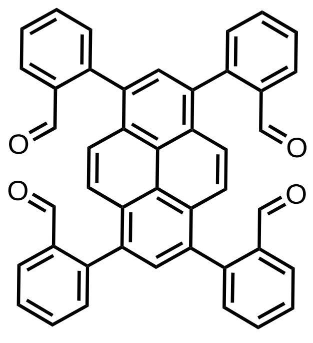

$C_{44}H_{26}O_4$
M = 618,69

A 100 mL round bottom flask equipped with a magnetic stir bar and a condenser was charged with 1,3,6,8-tetrabromopyrene **7** (1.00 g, 1.95 mmol), 2-formylphenylboronic acid **2** (1.40 g, 9.34 mmol), K_2CO_3 (2.58 g, 18.7 mmol) and $Pd(PPh_3)_4$ (90 mg, 78.0 µmol). The solids were suspended in 2:1 toluene/MeOH (40 mL), degassed and the atmosphere was exchanged by argon. The mixture was brought to reflux for 18 h. The mixture was diluted with CH_2Cl_2 (50 mL) and washed with H_2O (2x 50 mL). The aqueous layer was extracted with toluene (1x 50 mL) and the combined organics were dried over $MgSO_4$. The solvent was evaporated and the product was precipitated from CH_2Cl_2/hexanes. The solids were filtered, washed with hexanes and dried in vacuo, yielding the product as bright yellow solid in 81 % (980 mg, 1.58 mmol).

^{1}H NMR (400 MHz, $CDCl_3$, 293 K): δ = 9.78-9.72 (4H, m); 8.15-8.10 (4H, m); 7.99-7.95 (2H, m); 7.86-7.78 (4H, m); 7.77-7.70 (4H, m); 7.65-7.55 (8H, m).

^{13}C NMR (100 MHz, $CDCl_3$, 293 K): δ = 191.6; 191.5; 191.4; 191.34; 191.26; 191.2; 191.1; 143.34; 143.26; 143.2; 135.00; 134.98; 134.94; 134.92; 134.88; 134.85; 134.8; 134.0; 133.9; 133.8; 133.7; 133.6; 133.53; 133.46; 133.4; 133.32; 133.28; 132.31; 132.28; 132.2; 132.1;

130.9; 130.8; 130.0; 129.9; 128.7; 128.2; 128.1; 128.0; 127.9; 127.8; 127.74; 127.71; 126.13; 126.08; 124.7.

HRMS (APPI; MeCN, toluene): Chemical Formula: $C_{44}H_{26}O_4$ calc. 618.1826, found 618.1833.

1,3,6,8-Tetrakis(3-formylnaphthyl)pyrene (9)

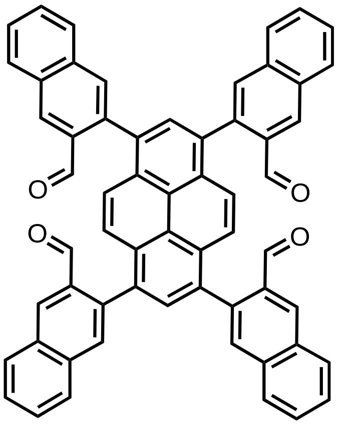

$C_{60}H_{34}O_4$
M = 818,93

A 5 mL septum vial equipped with a magnetic stir bar was charged with 1,3,6,8-tetrabromopyrene **7** (51.0 mg, 101 µmol), 2-formylnaphthyl-3-boronic acid pinacol ester **5** (125 mg, 443 µmol), K_2CO_3 (184 mg, 1.33 mmol) and $Pd(PPh_3)_4$ (12.0 mg, 10.1 µmol). The solids were suspended in 2:1 toluene/MeOH (3.0 mL), degassed and the atmosphere was exchanged by argon. The mixture was heated to 110 °C for 20 h. The mixture was diluted with CH_2Cl_2 (20 mL) and washed with H_2O (2x 10 mL). The aqueous layer was extracted with CH_2Cl_2 (1x 10 mL) and the combined organics were dried over $MgSO_4$. The solvent was evaporated and the product was precipitated from CH_2Cl_2/hexanes. The solids were filtered, washed with hexanes and dried in vacuo, yielding the product as bright yellow solid in 91 % (75 mg, 91.6 µmol).

^{1}H NMR (400 MHz, CD_2Cl_2, 293 K): δ = 9.99-9.82 (4H, m); 8.71-8.59 (4H, m); 8.19-8.07 (10H, m); 7.97 (4H, t, J=8.7 Hz); 7.89-7.83 (4H, m); 7.74-7.60 (8H, m).

^{13}C NMR (151 MHz, $CDCl_3$) δ 191.9, 191.8, 191.69, 191.65, 191.6, 191.53, 191.45, 191.4, 138.2, 138.09, 138.06, 138.05, 138.01, 138.00, 135.68, 135.65, 135.55, 135.54, 135.52, 135.51, 135.41, 135.39, 133.01, 132.95, 132.94, 132.87, 132.83, 132.77, 132.75, 132.7, 132.23, 132.21, 132.19, 131.7, 131.63, 131.57, 131.47, 131.45, 131.43, 131.38, 131.20, 131.16, 131.11, 131.05, 131.0, 130.9, 130.83, 130.78, 130.6, 130.5, 130.42, 130.38, 130.23, 130.20, 130.16, 130.13, 130.11, 130.04, 129.97, 129.93, 129.91, 129.88, 129.86, 129.8, 129.64, 129.60, 129.56, 129.52, 129.50, 129.4, 129.0, 128.2, 128.1, 128.03, 127.99, 127.97, 127.93, 127.90, 127.86, 127.8, 127.38, 127.36, 127.33, 127.30, 126.2, 126.12, 126.08, 126.06, 126.0, 125.3, 124.9, 124.8, 124.7. (mixture of rotamers)

HRMS (APPI; toluene): Chemical Formula: $C_{60}H_{34}O_4$ calc. 818.2452, found 818.2457.

2,2'-(naphthalene-1,4-diyl)dibenzaldehyde (10)

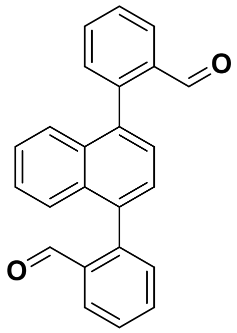

$C_{24}H_{16}O_2$
M = 336.39

A 100 mL round bottom flask equipped with a magnetic stir bar and a condenser was charged with 1,4-dibromonaphtalene (858 mg, 3 mmol), 2-formylphenylboronic acid **2** (1,125 g, 7.5 mmol), K_2CO_3 (2,07 g, 15 mmol) and $Pd(PPh_3)_4$ (69.0 mg, 60 µmol). The solids were suspended in 2:1 toluene/MeOH (30 mL), degassed and the atmosphere was exchanged by argon. The mixture was brought to reflux for 16 h. The mixture was diluted with DCM (60 mL) and washed with H_2O (2x 50 mL). The aqueous layer was extracted with DCM (1x 100 mL) and the combined organics were dried over $MgSO_4$. The solvent was evaporated and the product was purified by silica gel column chromatography, eluting with isohexane- isohexane:EtOAc (4:1) yielding 2,2'-(naphthalene-1,5-diyl)dibenzaldehyde as pale yellow solid in 95.6% (965 mg, 2,87 mmol)

^{1}H NMR (400 MHz, $CDCl_3$, 293 K) δ 9.73 (d, J = 0.8 Hz, 1H), 9.72 (d, J = 0.7 Hz, 0.8H), 8.16 (dt, J = 7.9, 1.6 Hz, 2H), 7.75 (tdd, J = 7.5, 4.3, 1.4 Hz, 2H), 7.64 (t, J = 7.6 Hz, 2H), 7.61 – 7.57 (m, 2H), 7.54 (dd, J = 7.0, 0.7 Hz, 2H), 7.52 – 7.49 (m, 2H), 7.48 – 7.42 (m, 2H).

^{13}C NMR (101 MHz, $CDCl_3$, 293 K) δ 191.9, 191.6, 143.8, 143.7, 136.17, 136.16, 134.8, 133.8, 133.7, 132.7, 131.8, 131.6, 128.4, 127.4, 127.30, 127.27, 127.26, 127.1, 127.0, 126.3.

HRMS (APPI; Toluene): Chemical Formula: $C_{24}H_{16}O_2$, calc. 336.1145, found 336.1156

2,2'-(naphthalene-1,5-diyl)dibenzaldehyde (12).

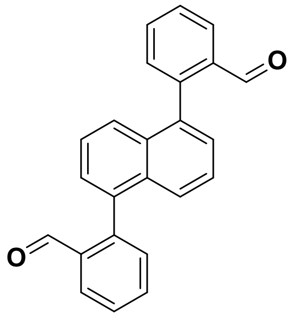

$C_{24}H_{16}O_2$
M = 336.39

A 100 mL round bottom flask equipped with a magnetic stir bar and a condenser was charged with 1,5-diiodonaphtalene (380 mg, 1 mmol), 2-formylphenylboronic acid **2** (375 mg, 2.5 mmol), K_2CO_3 (690 mg, 4.85 mmol) and $Pd(PPh_3)_4$ (23.0 mg, 20 µmol). The solids were suspended in 2:1 toluene/MeOH (9 mL), degassed and the atmosphere was exchanged by argon. The mixture was brought to reflux for 16 h. The mixture was diluted with DCM (30 mL) and washed with H_2O (2x 50 mL). The aqueous layer was extracted with DCM (1x 50 mL) and the combined organics were dried over $MgSO_4$. The solvent was evaporated and the product was purified by silica gel column chromatography, eluting with isohexane- isohexane:EtOAc (4:1) yielding 2,2'-(naphthalene-1,5-diyl)dibenzaldehyde as pale yellow solid in 38% (130 mg, 0.38 mmol)

R_f (SiO_2): 0.25 (4:1 isohexanes/EtOAc).

^{1}H NMR (400 MHz, $CDCl_3$, 293 K): δ 9.69 (d, J = 0.5 Hz, 1H), 9.67 (d, J = 0.4 Hz, 1H), 8.14 (ddd, J = 7.8, 4.4, 1.2 Hz, 2H), 7.79 – 7.70 (m, 2H), 7.67 – 7.56 (m, 4H), 7.54 – 7.42 (m, 6H).

^{13}C NMR (101 MHz, $CDCl_3$, 293 K): δ 191.9, 191.8, 144.04, 143.98, 136.0, 134.84, 134.82, 133.8, 133.7, 132.72, 132.70, 131.74, 131.71, 128.5, 128.4, 127.28, 127.25, 126.50, 126.48, 125.84, 125.82.

HRMS (APPI; Toluene): Chemical Formula: $C_{24}H_{16}O_2$ calc. 336.1145, found 336.1150.

[1,1':4',1''-terphenyl]-2,2''-dicarbaldehyde (14)

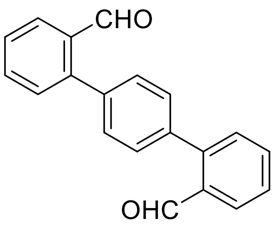

$C_{20}H_{14}O_2$
M = 286.33

A 100 mL round bottom flask equipped with a magnetic stir bar and a condenser was charged with 1,4-diodobenzene (989mg, 3 mmol), 2-formylphenylboronic acid **2** (1.125 g, 7.5 mmol), K_2CO_3 (2.07 g, 14.5 mmol) and $Pd(PPh_3)_4$ (69.0 mg, 59 µmol). The solids were suspended in 2:1 toluene/MeOH (30 mL), degassed and the atmosphere was exchanged by argon. The mixture was brought to reflux for 16 h. The mixture was diluted with DCM (100 mL) and washed with H_2O (2x 50 mL). The aqueous layer was extracted with DCM (2x 50 mL) and the combined organics were dried over $MgSO_4$. The solvent was evaporated and the product was purified by silica gel column chromatography, eluting with isohexane- isohexane:EtOAc (4:1) yielding [1,1':4',1''-terphenyl]-2,2''-dicarbaldehyde as pale yellow solid in 46% (400 mg, 1.4 mmol)

R_f (SiO_2): 0.25 (4:1 isohexanes/EtOAc).

^{1}H NMR (400 MHz, CD_2Cl_2, 293 K): δ 10.10 (s, 2H), 8.07 (dd, *J* = 7.7, 1.0 Hz, 2H), 7.74 (td, *J* = 7.5, 1.4 Hz, 2H), 7.64 – 7.51 (m, 8H).

^{13}C NMR (101 MHz, $CDCl_3$, 293 K): δ 192.2, 145.0, 137.7, 133.71, 133.69, 130.8, 130.1, 128.1, 127.9.

4,4'-(3,8-bis(2-formylphenyl)pyrene-1,6-diyl)dibenzaldehyde (16)

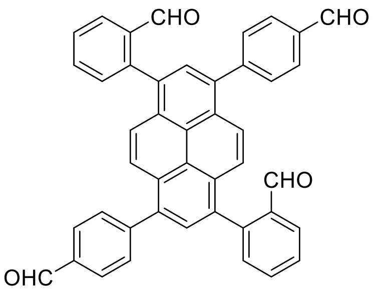

$C_{44}H_{26}O_4$
M = 618.68

A 25 mL round bottom flask equipped with a magnetic stir bar was charged with 1,6-dibromo-3,8-bis(2-formylphenyl)pyrene **6** (20 mg, 35 µmol), 4-formylboronic acid (20 mg, 133 µmol), K_2CO_3 (21 mg, 152 µmol), $Pd(PPh_3)_4$ (1 mg, 0.85 µmol) The solids were suspended in 2:1 toluene/MeOH (1.5 mL), degassed and the atmosphere was exchanged by argon. The mixture was heated to 80 °C for 18 h. The mixture was diluted with CH_2Cl_2 (25 mL) and washed with H_2O (30 mL). The aqueous layer was extracted with CH_2Cl_2 (1x 30 mL) and the combined organics were dried over $MgSO_4$. was purified by silica gel column chromatography, eluting with isohexane- isohexane:EtOAc (3:1) yielding 4,4'-(3,8-bis(2-formylphenyl)pyrene-1,6-diyl)dibenzaldehyde as pale yellow solid in 69% (15mg, 24.2 mmol)

R_f (SiO_2): 0.18 (3:1 isohexanes/EtOAc).

^{1}H NMR (400 MHz, CD_2Cl_2, 293 K) δ 10.13 (s, 2H), 9.74 (dd, *J* = 6.5, 0.6 Hz, 2H), 8.19 (d, *J* = 9.5 Hz, 2H), 8.15 (dd, *J* = 7.8, 1.2 Hz, 2H), 8.09 – 8.03 (m, 6H), 7.85 (dd, *J* = 8.8, 4.0 Hz, 5H), 7.81 (td, *J* = 7.5, 1.4 Hz, 3H), 7.66 (ddd, *J* = 9.9, 7.6, 4.2 Hz, 4H).

^{13}C NMR (101 MHz, $CDCl_3$, 293 K): δ 191.9, 191.71, 191.6, 146.5, 143.72, 143.68, 136.4, 135.5, 135.0, 134.9, 133.9, 133.8, 133.5, 132.3, 132.2, 132.1, 131.4, 130.00, 129.99, 129.9, 128.7, 128.5, 127.8, 126.1, 125.8, 125.2, 125.20.

1,6-bis(2-formylphenyl)-3,8-bis(4-pyridyl)pyrene (S5)

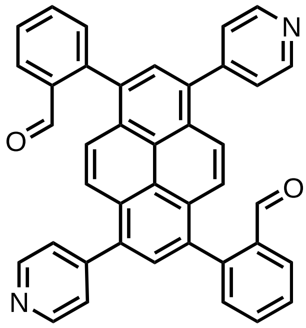

$C_{40}H_{24}N_2O_2$
M = 564,64

A 5 mL septum vial equipped with a magnetic stir bar was charged with 1,6-dibromo-3,8-bis(2-formylphenyl)pyrene **6** (130 mg, 229 µmol), 4-pyridinylboronic acid pinacol ester (103 mg, 503 µmol), K_2CO_3 (138 mg, 1.00 mmol), $Pd(PPh_3)_2Cl_2$ (4.0 mg, 5.70 µmol) and PPh_3 (3 mg, 11.5 µmol). The solids were suspended in 2:1 toluene/MeOH (3.0 mL), degassed and the atmosphere was exchanged by argon. The mixture was heated to 80 °C for 18 h. The mixture was diluted with CH_2Cl_2 (25 mL) and washed with H_2O (2x 10 mL). The aqueous layer was extracted with CH_2Cl_2 (1x 10 mL) and the combined organics were dried over $MgSO_4$. The product was precipitated from CH_2Cl_2/hexanes, yielding the product as pale yellow solid in 91 % (117 mg, 207 µmol).

^{1}H NMR (400 MHz, CD_2Cl_2, 293 K): δ = 9.73 (1H, s); 9.72 (1H, s); 8.76 (4H, d, *J*=4.84 Hz); 8.19 (2H, d, *J*=9.52 Hz); 8.15 (2H, d, *J*=7.60 Hz); 8.02 (2H, s); 7.86 (2H, d, *J*=9.52 Hz); 7.81 (2H, t, *J*=7.10 Hz); 7.69 (2H, d, *J*=15.16 Hz); 7.66-7.57 (6H, m).

^{13}C NMR (100 MHz, CD_2Cl_2, 293 K): δ = 191.52; 191.48; 150.1; 148.1; 143.60; 143.56; 135.13; 135.09; 135.05; 133.92; 133.90; 133.85; 132.30; 132.25; 132.0; 131.9; 130.2; 129.9; 128.8; 128.6; 128.5; 128.3; 127.8; 127.7; 125.9; 125.8; 125.5; 125.2.

HRMS (APPI; toluene): Chemical Formula: $C_{40}H_{24}N_2O_2$ calc. 564.1832, found 564.1845

(pyrene-1,6-diylbis(2,1-phenylene))bis(phenylmethanone) (S6).

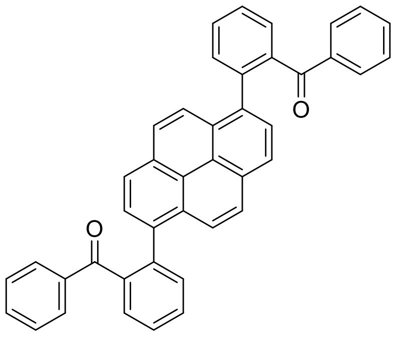

$C_{42}H_{26}O_2$
M = 562.6680

A 100 mL round bottom flask equipped with a magnetic stir bar and a condenser was charged with 1,6-dibromopyrene **1** (120 mg, 0.336 mmol), phenyl(2-(4,4,5,5-tetramethyl-1,3,2-dioxaborolan-2-yl)phenyl)methanone **S4** (250 mg, 0.84 mmol), K_2CO_3 (230 mg, 1.61 mmol) and $Pd(PPh_3)_4$ (8 mg, 6.9 µmol). The solids were suspended in 2:1 toluene/MeOH (15 mL), degassed and the atmosphere was exchanged by argon. After refluxing for 16 h, the mixture was diluted with DCM (60 mL) and washed with H_2O (50 mL). The aqueous layer was extracted with DCM (50 mL) and dried over $MgSO_4$. The solvent was evaporated and the product was purified by HPLC (PBr column, DCM:MeOH 3:7, 1 mL/min, 40°C), yielding (pyrene-1,6-diylbis(2,1-phenylene))bis(phenylmethanone) as pale yellow solid in 35% (60 mg, 0.106 mmol).

The precursor has shown no formation of diphenyl-DBATT under DPEX condition.

^{1}H NMR (400 MHz, CD_2Cl_2, 293 K) δ 8.00 – 7.85 (m, 6H), 7.76 (dd, *J* = 7.8, 1.8 Hz, 2H), 7.70 (dddd, *J* = 5.6, 4.9, 2.8, 1.3 Hz, 4H), 7.66 – 7.58 (m, 4H), 7.53 – 7.44 (m, 4H), 7.23 – 7.13 (m, 2H), 7.11 – 7.00 (m, 4H).

^{13}C NMR (101 MHz, CD_2Cl_2, 293 K) δ 198.2, 198.1, 140.9, 140.8, 140.3, 137.74, 137.73, 136.15, 136.11, 132.79, 132.75, 132.59, 132.55, 130.7, 130.50, 130.46, 129.8, 129.7, 129.44, 129.43, 129.32, 129.28, 128.65, 128.61, 128.11, 128.08, 127.8, 127.7, 125.64, 125.63, 124.8, 124.51, 124.49.

HRMS (APPI; Toluene): Chemical Formula: $C_{42}H_{26}O_2$, calc. 562.1927, found 562.1927

Preparation of zig-zag nanographenes

General procedure

A 250 mL round bottom flask equipped with a magnetic stir bar is charged with the precursor (**3**, **4**, **6**, **S3**, **8**, or **9**) (20 mg) and dissolved in CH_2Cl_2 (100 mL). While stirring, a solution of $SnCl_2 \cdot 2H_2O$ (500 mg, 2.22 mmol) in *i*-PrOH (2.0 mL) is added at rt. At this point a slight color change to yellowish is typically observed. Then, conc. H_2SO_4 (1.0 mL) is added and the mixture is allowed to stir at rt with protection from daylight for 18 h. A dark purple coloration and the formation of a white precipitate is typically observed. The dark purple mixture is quenched with 1 M HCl (2.0 mL). Vigorous mixing is necessary at this point. The mixture is diluted with CH_2Cl_2 (20 mL) and washed with H_2O (1x 50 mL). The aqueous layer is extracted with CH_2Cl_2 (3x 20 mL) including the dark insoluble solids (product). The combined CH_2Cl_2 layers are diluted with MeOH (100 mL) and the CH_2Cl_2 is removed on the rotavap at atmospheric pressure at 50 °C. The formed precipitate in the MeOH layer is centrifuged, the MeOH layer is decanted and the solid is washed again with MeOH. The product is dried in vacuo and obtained in quantitative yield.

2.3,8.9-Dibenzanthanthrene (DBATT)

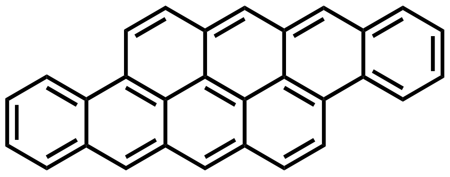

$C_{30}H_{16}$
M = 376,46

3 (20.0 mg; 48.7 µmol); yield: 98 % (18.0 mg, 47.8 µmol).

^{1}H NMR (400 MHz, *o*-DCB-D$_4$, 373 K): δ = 9.02-8.88 (6H, m); 8.84 (2H, m); 8.38-8.29 (4H, m); 7.79-7.67 (4H, m).

HRMS (MALDI): Chemical Formula: $C_{30}H_{16}$ calc. 376.1247; without matrix 376.1798; 377.1836; DHB matrix 376.1790; 377.1826; DCTB matrix 376.1764; 377.1817.

UV/Vis (CH_2Cl_2, 293 K): λ [nm] (ε [$M^{-1}cm^{-1}$]) = 295 (24700), 327 (sh), 342 (112100), 358 (sh), 467 (7100), 506 (8920), 542 (19300), 586 (40500).

Fluo (CH_2Cl_2, 293 K): λ [nm] (rel. int. [%]) = 596 (100), 640 (59).

EA Calculated: C, 95.72; H, 4.28, Found: C, 94.88; H, 4.54.

6,14-Bisphenyl-2.3,8.9-dibenzanthanthrene (bPh-DBATT)

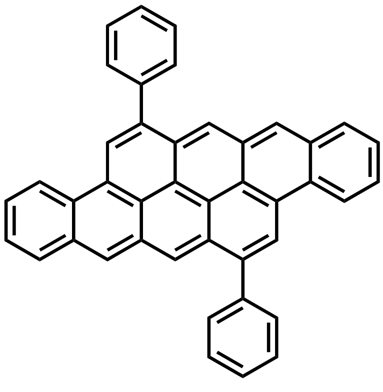

$C_{42}H_{24}$
M = 528,65

3a (20.0 mg; 35.5 µmol); yield: 97 % (18.2 mg, 34.4 µmol).

^{1}H NMR (400 MHz, *o*-DCB-D$_4$, 373 K): δ = 8.99 (2H, s); 8.94-8.84 (4H, m); 8.68 (2H, s); 8.19 (2H, d, *J*=6.28 Hz); 7.96 (4H, d, *J*=5.60 Hz); 7.71-7.54 (10H, m).

MS (LDI): *m/z* (*rel.* int.) = 528.20 [M]$^+$ (*100*).

HRMS (APPI; CH_2Cl_2, toluene): Chemical Formula: $C_{42}H_{24}$ calc. 528.1873, found 528.1881.

6,14-Bisbromo-2.3,8.9-dibenzanthanthrene (bBr-DBATT)

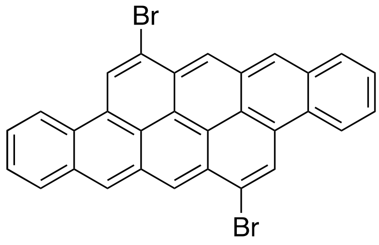

$C_{30}H_{14}Br_2$
M = 534,25

6 (20.0 mg; 35.2 µmol); yield: 95 % (17.5 mg, 33.4 µmol).

MS (LDI): *m/z* (*rel.* int.) 533.94 [M]$^+$ (*100*).

HRMS (APPI; dichlorobenzene): Chemical Formula:

UV/Vis (Toluene, 293 K): λ [nm]) = 333, 346, 510, 548, 592

6,14-Bis(*para*-formylphenyl)-2.3,8.9-dibenzanthanthrene (17)

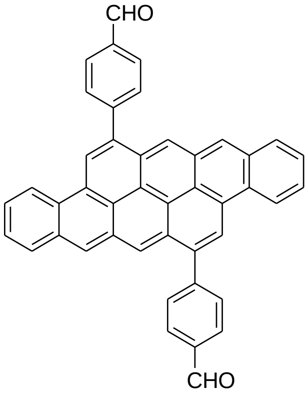

$C_{44}H_{24}O_2$
M = 584.67

16 (15.0 mg; 24.2 µmol); yield: 92 % (13 mg, 22.2 µmol).

Compound is insufficiently soluble for NMR measurements

MS (LDI): *m/z* (*rel.* int.) 584.08 [M]$^+$ (*100*).

HRMS (APPI; dichlorobenzene): Chemical Formula: $C_{44}H_{24}O_2$, calc. 584.1771, found 584.1758.

UV/Vis (Toluene, 293 K): λ [nm]) = 333, 347, 518, 552, 597.

6,14-Bis(4-pyridinyl)-2.3,8.9-dibenzanthanthrene (S7)

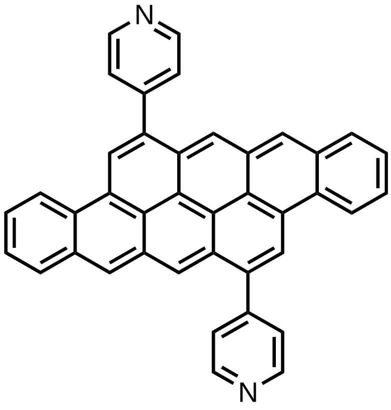

$C_{40}H_{22}N_2$
M = 530,63

S5 (20.0 mg; 35.4 µmol); yield: 95 % (17.8 mg, 33.5 µmol).

^{1}H NMR (500 MHz, $C_6D_4Cl_2$, 120 °C) δ 8.99 (d, *J* = 5.7 Hz, 2H), 8.92 (d, *J* = 7.7 Hz, 2H), 8.78 (s, 1H), 8.32 (d, *J* = 9.2 Hz, 2H), 8.23 (d, *J* = 8.2 Hz, 2H), 8.10 – 8.04 (m, 2H), 7.90 (d, *J* = 9.2 Hz, 2H), 7.86 (d, *J* = 7.6 Hz, 2H), 7.83 – 7.78 (m, 2H), 7.74 – 7.64 (m, 5H).

MS (LDI): *m/z* (*rel. int.*) = 530.24 $[M]^+$ (*100*).

HRMS (APPI; MeCN, toluene): Chemical Formula: $C_{40}H_{22}N_2$ calc. 530.1778, found 530.1778.

UV/Vis (Toluene, 293 K): λ [nm]) = 332, 347, 518, 552, 597

Tetrakis-tetracene (TTc)

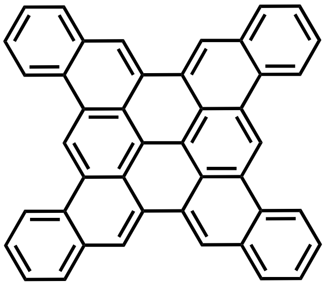

$C_{44}H_{22}$
M = 550,66

8 (20.0 mg; 32.3 µmol); yield: 96 % (17.1 mg, 31.1 µmol).

MS (LDI): *m/z* (*rel. int.*) = 549.95 $[M]^+$ (*100*).

HRMS (APPI; toluene): Chemical Formula: $C_{44}H_{22}$ calc. 550.1716, found 550.1713.

EA Calculated: C, 95.62; H, 4.38, Found: C, 94.93; H, 4.12.

Bis-pentacene (BPc)

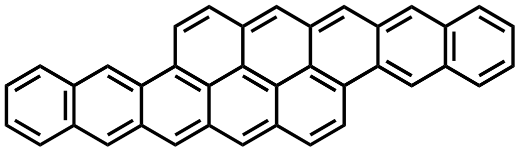

$C_{38}H_{20}$
M = 476,58

4 (20.0 mg; 39.2 µmol); yield: 97 % (18.2 mg, 38.1 µmol).

MS (LDI): *m/z* (*rel. int.*) = 476.23 $[M]^+$ (*100*).

HRMS (APPI): Chemical Formula: $C_{38}H_{20}$ calc. 476.1558, found 476.550.

Tetrakis-pentacene (TPc)

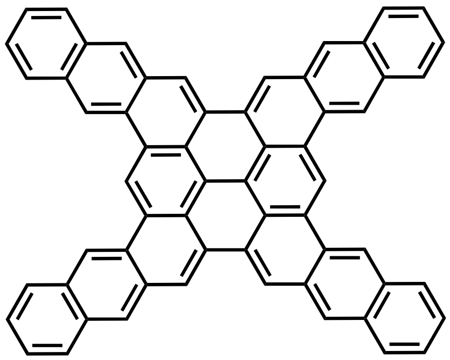

$C_{60}H_{30}$
M = 750,90

9 (20.0 mg; 24.4 µmol); yield: 96 % (17.6 mg, 23.4 µmol).

MS (LDI): *m/z* (*rel. int.*) = 750.33 $[M]^+$ (*100*).

HRMS (APPI): Chemical Formula: $C_{60}H_{30}$ calc. 750.2342, found 750.2351.

Synthesis of dibenzopyrenes

A 250 mL round bottom flask equipped with a magnetic stir bar was charged with the precursor (20 mg) dissolved in CH_2Cl_2 (100 mL). While stirring, a solution of $SnCl_2 \cdot 2H_2O$ (500 mg, 2.22 mmol) in *i*-PrOH (2.0 mL) was added. Then 1.0 mL of conc. H_2SO_4 was added and the mixture was allowed to stir at rt with protection from daylight for 18 h. After quenching with 1 M HCl (2.0 mL), the mixture was diluted with CH_2Cl_2 (20 mL) and washed with H_2O (1x 50 mL). The aqueous layer was extracted with CH_2Cl_2 (3x 20 mL). The organic layers were combined and evaporated under reduced pressure. The residue was purified by silicagel column chromatography, eluting with isohexane-DCM 10:1, yielding dibenzopyrene as a yellow solid.

Benzo[*rst*]pentaphene (11)

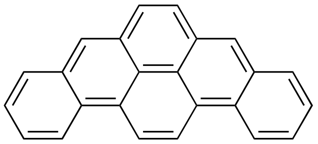

$C_{24}H_{14}$
M = 302,38

2,2'-(naphthalene-1,4-diyl)dibenzaldehyde **10** (20.0 mg; 59.5 µmol); yield: 61 % (11 mg, 36.4 µmol).

^{1}H NMR (400 MHz, CD_2Cl_2, 293 K) δ 9.26 (s, 2H), 9.07 (d, *J* = 8.2 Hz, 2H), 8.36 (s, 2H), 8.25 (d, *J* = 7.8 Hz, 2H), 7.88 – 7.76 (m, 6H).

^{13}C NMR (101 MHz, CD_2Cl_2, 293 K) δ 132.0, 130.7, 129.2, 129.0, 128.9, 128.5, 127.0, 126.7, 125.3, 125.0, 123.4, 122.8.

MS (LDI): *m/z* (*rel. int.*) = 302.30 $[M]^+$ (*100*).

HRMS (APPI; Toluene): Chemical Formula: $C_{24}H_{14}$, calc. 302.1090, found 302.1096

UV/Vis (CH_2Cl_2, 293 K): λ [nm]) = 285, 296, 315, 327, 331, 354, 373, 395

Dibenzo[*b,def*]chrysene (13)

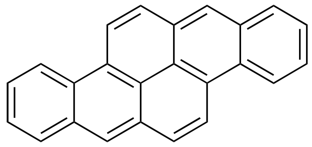

$C_{24}H_{14}$
M = 302,38

2,2'-(naphthalene-1,5-diyl)dibenzaldehyde **12** (20.0 mg; 59.5 µmol); yield: 66 % (12 mg, 39.6 µmol).

^{1}H NMR (400 MHz, CD_2Cl_2, 293 K): δ 9.04 (dd, *J* = 19.1, 8.9 Hz, 4H), 8.76 (s, 2H), 8.37 (dd, *J* = 13.9, 8.5 Hz, 4H), 7.83 (dddd, *J* = 22.6, 7.9, 6.7, 1.3 Hz, 4H).

^{13}C NMR (101 MHz, CD_2Cl_2, 293 K): δ 131.9, 130.3, 129.3, 128.8, 128.4, 127.7, 126.43, 126.41, 125.5, 124.5, 123.5, 122.9.

MS (LDI): *m/z* (*rel. int.*) = 302.34 $[M]^+$ (*100*).

HRMS (APPI; Toluene): Chemical Formula: $C_{24}H_{14}$, calc. 302.1090, found 302.1092

UV/Vis (CH_2Cl_2, 293 K): λ [nm]) = 297, 398, 442, 448.

Bulk synthesis of 2.3,8.9-Dibenzanthanthrene (DBATT)

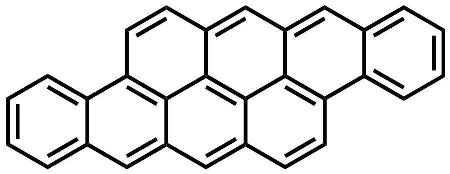

$C_{30}H_{16}$
M = 376,46

A 4 L round bottom flask equipped with a magnetic stir bar was charged with 1,6-bis(2-formylphenyl)pyrene **3** (0.50 g, 1.22 mmol) and dissolved in 2.5 L CH_2Cl_2. A solution of $SnCl_2 \cdot 2H_2O$ (12.5 g, 55.4 mmol) dissolved in *i*-PrOH (50.0 mL) was added, followed by the addition of conc. H_2SO_4 (25.0 mL). The mixture was stirred for 18 h. 1 M HCl (50.0 mL) was added and the solution was mixed thoroughly. H_2O (1.0 L) was added and the mixture was extracted with CH_2Cl_2 (5x 200 mL). The organic layer was diluted with MeOH (500 mL) and the CH_2Cl_2 layer was evaporated on the rotavap. The remaining MeOH layer was centrifuged, the supernatant was decanted and the solid was washed again with MeOH. The dark blue solid was dried in vacuo, giving **DBATT** in 96 % (0.44 g, 1.17 mmol).

DBATT could be sublimed (310 °C, 10^{-5} mbar) yielding dark blue crystalline needles of several micrometer-length (figure S2). Sublimation caused some decomposition to black insoluble solids, leading to an isolated yield of 79 % (0.36 g, 0.96 mmol).

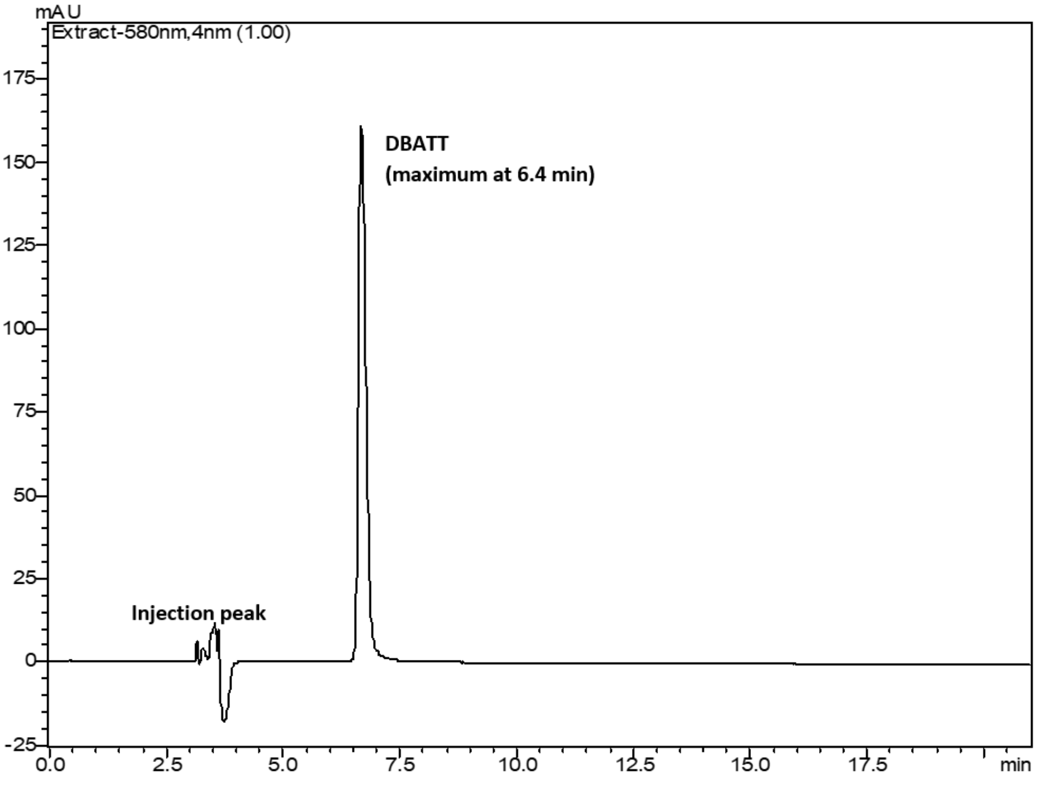

Supplementary Figure 3. HPLC profile of **DBATT** (bulk reaction) after aqueous work-up of reaction; detected at 580 nm (PBr column, toluene:MeOH:4:1 as eluent, 1 mL/min, 35 °C).

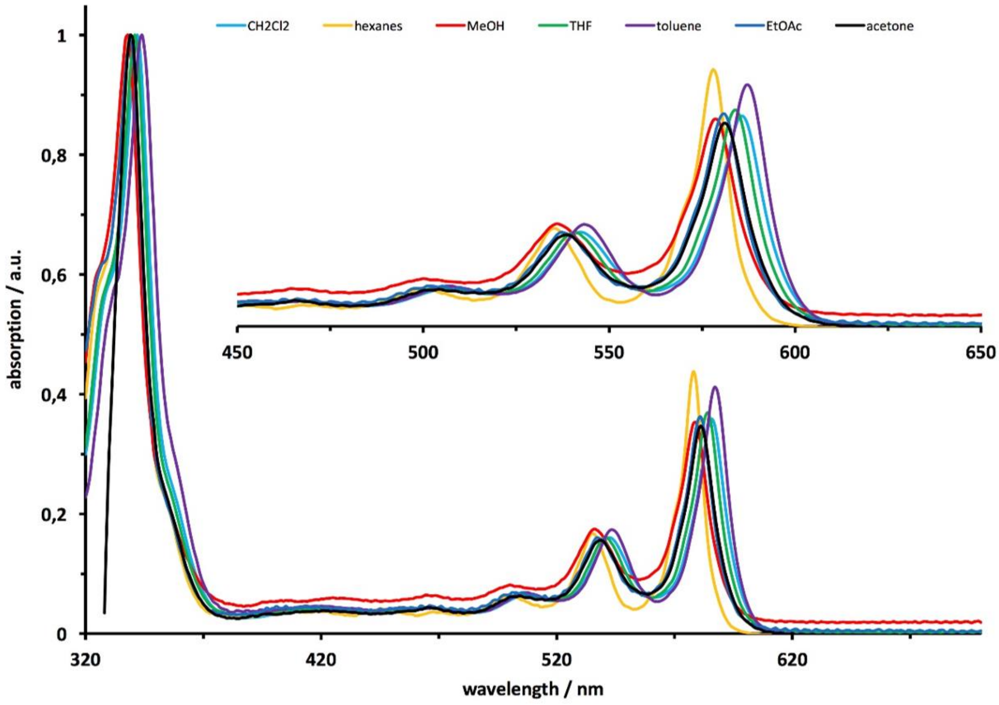

Supplementary Figure 4. Absorption spectra of **DBATT** at rt in various solvents.

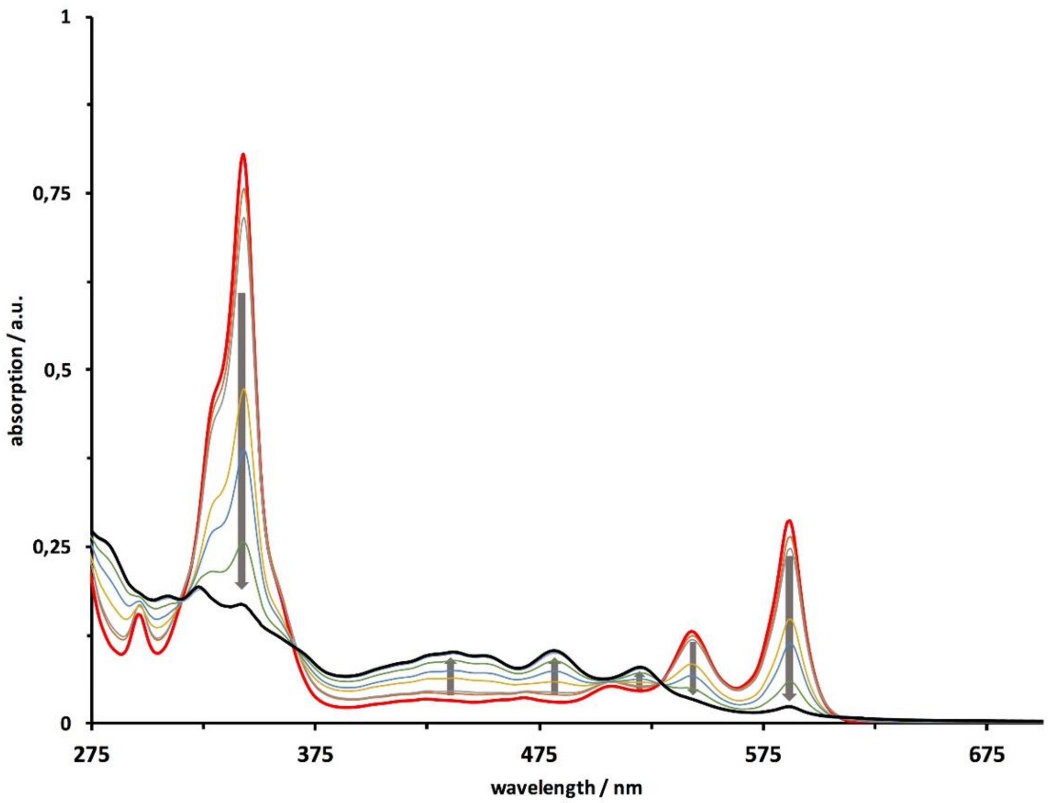

Supplementary Figure 5. Time-dependent photo-oxidative decomposition study of **DBATT** in CH_2Cl_2 at rt, after exposure to air and daylight in a timeframe of 24 h.

Crystallographic Data of DBATT

Synchrotron X-ray data were collected at the BESSY storage ring (BL14.2, PFS, Berlin, Germany) using a Pilatus2M pixel detector, λ = 0.8266 Å. $C_{30}H_{16}$: monoclinic, $P2_1/n$, a = 9.540(1) Å, b = 3.8311(4) Å, c =23.483(2) Å, β= 98.553(9)°, V = 848.73(15) Å^3, Z = 2, R_1(F)/ wR_2(F2) = 0.124/0.319 for 2346/1223 reflections and 136 parameters.

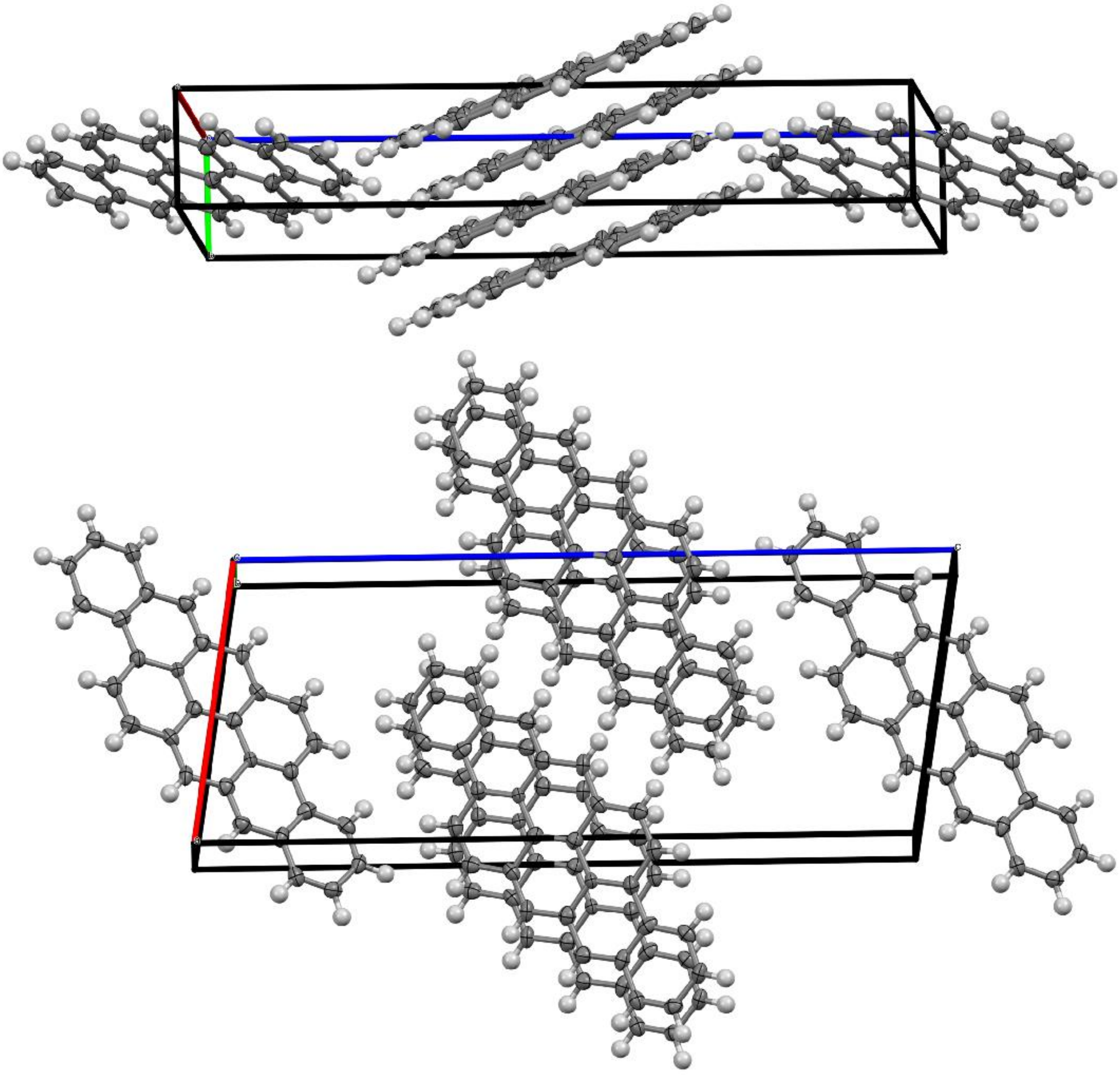

Supplementary Figure 6. Crystal structure of **DBATT**. Thermal ellipsoids are set at 50% probability level.

Scanning Tunneling Microscopy

STM Measurements

All low-temperature STM experiments were performed in a two-chamber ultra-high vacuum (UHV) system from Scienta-Omicron GmbH that is operated at a base pressure below 1×10^{-10} mbar. The STM measurements were conducted in the constant current mode at a sample temperature of around 4.7 K or 77 K, respectively. The bias voltages mentioned here refer to the sample, which was grounded in the experiment. The STM topography images were analyzed with the WSxM software.[2]

Sample Preparation

The Ag(111) and Au(111) (from MaTecK) single crystals were cleaned by subsequent cycles of Ar^+ ion sputtering (1 keV) and annealing at 750 K, respectively. The **TTc**, **BPc**, and **TPc** were thermally evaporated from a commercial Knudsen cell (Kentax GmbH) with the quartz crucible held at 350°C, 300°C, and 395°C, respectively. The molecules were thoroughly degassed before the deposition on the clean metal surface. The evaporation rates were determined by a quartz crystal microbalance. The metal substrates were kept at room temperature during the evaporation of molecules.

Self-assembly of TTc on Au(111)

Figure S7a shows that the deposition of nearly a monolayer of **TTc** on Au(111) held at room temperature results in the formation of a close-packed self-assembly. The **TTc** monomers within the islands can be identified by the x-shape of the carbon backbone. Along the direction of the short axis of the x-shaped carbon backbone, the molecules form perfectly ordered columns. Weak intermolecular van der Waals forces are presumably responsible for the formation of the well-ordered columns. We observe two rotational domains of the assembly, which are distinguished by a rotation of the end-to-end connection between the branches of two neighboring molecules (highlighted by the green lines). The corresponding unit cells are shown in figure S7b, where the molecules are connected horizontally in the unit cell highlighted in red and the other domain (blue unit cell) has a vertical connection line. The rhombic unit cell measures $a = 1.44 \pm 0.11$ nm, $b = 1.64 \pm 0.13$ nm and $\Theta = 77° \pm 3°$ for both domains. The domain boundary line is indicated by a white line in figure S7b. The Au(111) herringbone reconstruction,[3] is clearly visible through the self-assembled molecular overlayer and seems not affected by adsorption of the molecules. The intact herringbone reconstruction of the Au(111) surface underneath the adsorbed molecules is generally considered as a sign for a weak molecule-substrate interaction suggesting a physisorption-type interaction.[4]

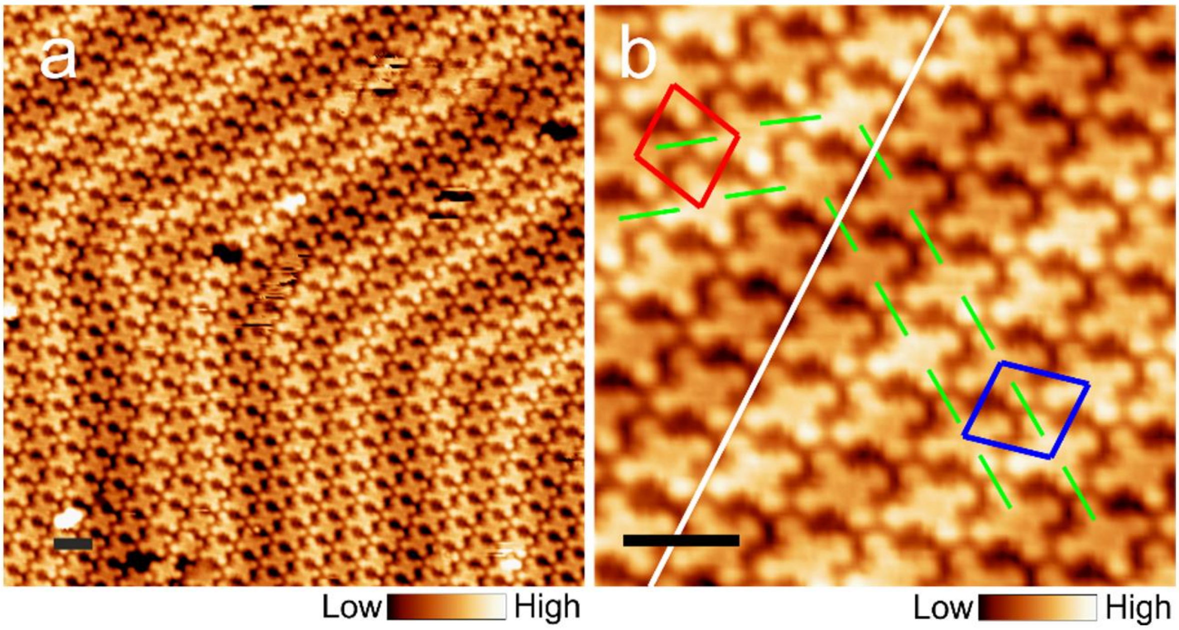

Supplementary Figure 7. Low-temperature STM images (77 K) of self-assembled **TTc** on Au(111). (a) Overview STM image of the **TTc** self-assembly. (b) Detailed STM image showing the two rotational domains (red and blue unit cell) of the **TTc** self-assembly. The two domains are distinguished by a rotation of α = 120° ± 4.2° of the end-to-end connection between the branches of two neighboring molecules (highlighted by the green lines). Scale bars: 2 nm. Tunneling conditions: a) 100 pA/1 V; b) 100 pA/1 V.

Adsorption of TPc on Ag(111)

The overview STM image in figure S8a shows **TPc** molecules adsorbed on Ag(111) after deposition at room temperature. Two kinds of molecules are observed. The majority is x-shaped, while there are also a few y-shaped molecules co-adsorbed. The y-shaped species is considered as a **TPc** with one naphthyl fragment missing (three-fold coupled precursor instead of four-fold), most probably being a minor byproduct during the synthesis process. The y-shaped molecule is expected to sublime at lower temperature than the x-shaped molecule and therefore observable in room temperature depositions. The size of the x-shaped molecules from the STM image is consistent with the size of the **TPc**. Besides the two kinds of molecules, bright protrusions are observed on Ag(111). Figure S8b shows a detailed STM image of the bright protrusions, which might arise from halogen atoms that could be residues from the synthesis. The protrusions form chains as well as networks (highlighted by the white circles in figure S8b). The networks have a $\sqrt{\mathbf{3}} \times \sqrt{\mathbf{3}}$ structure with 0.46 ± 0.2 nm spacing of neighboring atoms, which is consistent with the previously observed halogen-halogen distances on Ag(111).[5,6] The amount of halogen residue was significantly reduced by degassing the compound under UHV conditions. To avoid lager amounts of dissociated halogens on the surface, we propose experiments with surfaces preheated above the desorption temperature of the halogens (>250 °C) a strategy that was successfully used for growing graphene from hexabromobenzene on Cu(111).[7] Under these conditions, perturbing halogens are supposed to desorb, while the **TPc** molecules are expected to stick to the surface owing to their larger mass. However, chemical reactions within and among the molecules cannot be excluded by the deposition at elevated sample temperatures and trace amounts of byproducts from the synthesis are less likely to be seen. Therefore, we preferred here to use room temperature

evaporations for the structural identification of the molecules and the analysis of possible byproducts. We note that the overall trace concentration of halogen is small as confirmed by mass spectrometry but is pronounced in this preparation technique.

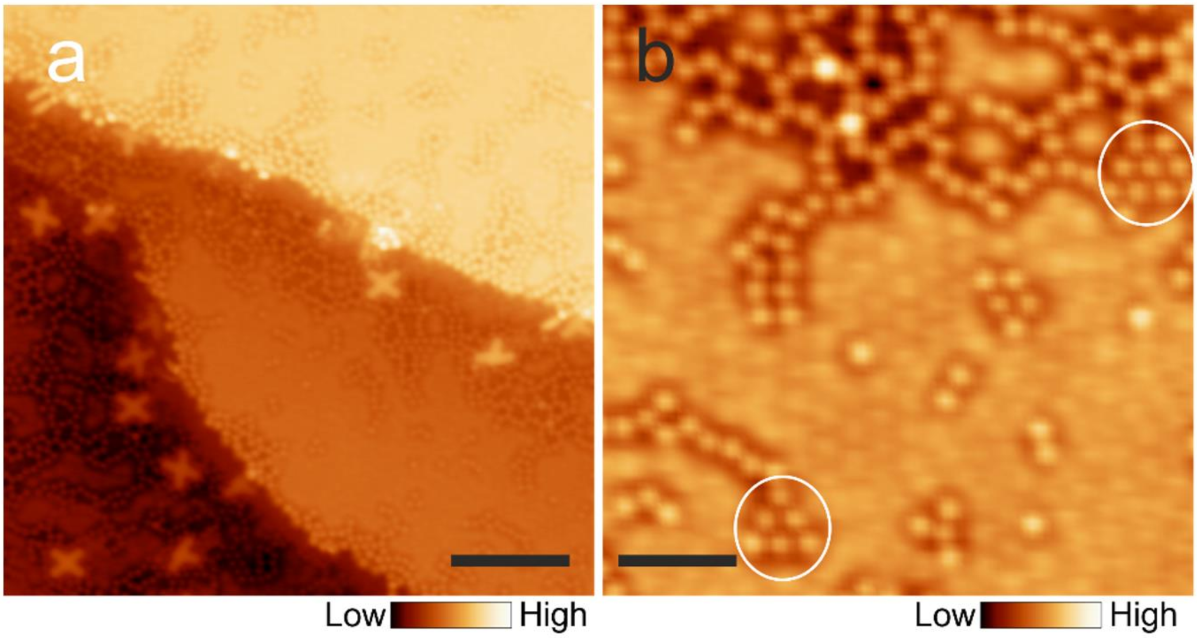

Supplementary Figure 8. Low-temperature STM (4.7 K) images of **TPc** on Ag(111). (a) Large-scale STM image of **TPc**. (b) Detailed STM image of the coadsorbed halogens. Scale bar: (a) 6 nm and (b) 2nm. Tunneling conditions: (a) 100 pA/-0.5 V and (b) 100 pA/-0.5 V.

Computations

Geometries were optimized at the DFT R(U)B3LYP 6-311+G(d,p) level of theory (RB3LYP for closed shell structure, UB3LYP for open shell structure) using the Gaussian '09 work package.[1]

Closed shell	`%chk=DBATT RDFT6311G+dp.chk`<br>`# opt rb3lyp/6-311+g(d,p) geom=connectivity`
Open shell	`%chk=DBATT UDFT6311G+dp.chk`<br>`# opt ub3lyp/6-311+g(d,p) guess=mix geom=connectivity pop=no`

Tetracene, **pentacene**, **DBATT**, **TTc** and **TPc** revealed a closed shell singlet ground, whereas **hexacene** and **BPc** favored an open shell singlet ground state (bold energies). The occupation number of the HONO and LUNO were obtained from the DFT-optimized minimum structures and evaluated at the UHF 6-31+G(d,p) level of theory.[8] Diradical character y_0 was obtained from equation S1 and the Eigenvalues of the respective HONOs and LUNOs.[9]

Occupation number	`%chk=DBATT popnoHFgeoRDFT.chk`<br>`# uhf/6-31+g(d,p) guess=mix geom=connectivity pop=no`

Supplementary Equation 1: $y = 1 - \frac{4(\sigma_{HOMO} - \sigma_{LUMO})}{4 + (\sigma_{HOMO} - \sigma_{LUMO})^2}$

Supplementary Table 2. Summary on computational evaluation of the nanographenes.

	**Geometry Opt (DFT B3LYP 6-311+G(d,p))**		**Occupation Number (HF 6-31+G(d,p))**		**Diradical character**
	**Total Energy [Hartree]**		**Eigenvalue [Hartree]**		
Compound	***Singlet CS***	***Singlet OS***	***(HONO)***	***(LUNO)***	y_0
**Tetracene**	**-693.32918487**	-693.32918487	1.43486 (60)	0.56514 (61)	0.27
**Pentacene**	**-846.99687572**	-846.99687572	1.32936 (73)	0.67064 (74)	0.41
**Hexacene**	-1000.6640097	**-1000.66404451**	1.24197 (86)	0.75803 (87)	0.54
**DBATT**	**-1153.17691852**	-1153.17691852	1.30722 (98)	0.69278 (99)	0.44
**BPc**	-1451.01730924	**-1460.51725308**	1.20605 (124)	0.79395 (125)	0.60
**TTc**	**-1690.4566136**	-1690.4566136	2.00000 (143)	0.00000 (144)	0.00
**TPc**	**-2305.15381843**	-2305.14287094	2.00000 (195)	0.00000 (196)	0.00

The ^{1}H NMR spectrum of **DBATT** was calculated using the optimized structure at the DFT RB3LYP 6-311+G(d,p) level of theory.

NMR	%chk=DBATT GSRs NMR.chk<br># nmr=giao b3lyp/6-311+g(d,p) geom=connectivity

Summary of NMR spectra (SCF GIAO Magnetic shielding)
Values for element H only
Reference: TMS B3LYP/6-311+G(2d,p) GIAO
Reference shielding: 31.8821 ppm
Degenerate peaks are condensed together (Degeneracy Tolerance 0.05)

**Shift (ppm)**	**Degeneracy**	**Atoms**
9.5031921242	4.0000	38,46,40,31
9.4096116915	2.0000	35,39
9.3075335409	2.0000	36,44
8.6857793051	4.0000	37,45,32,43
8.0115664372	4.0000	41,33,42,34

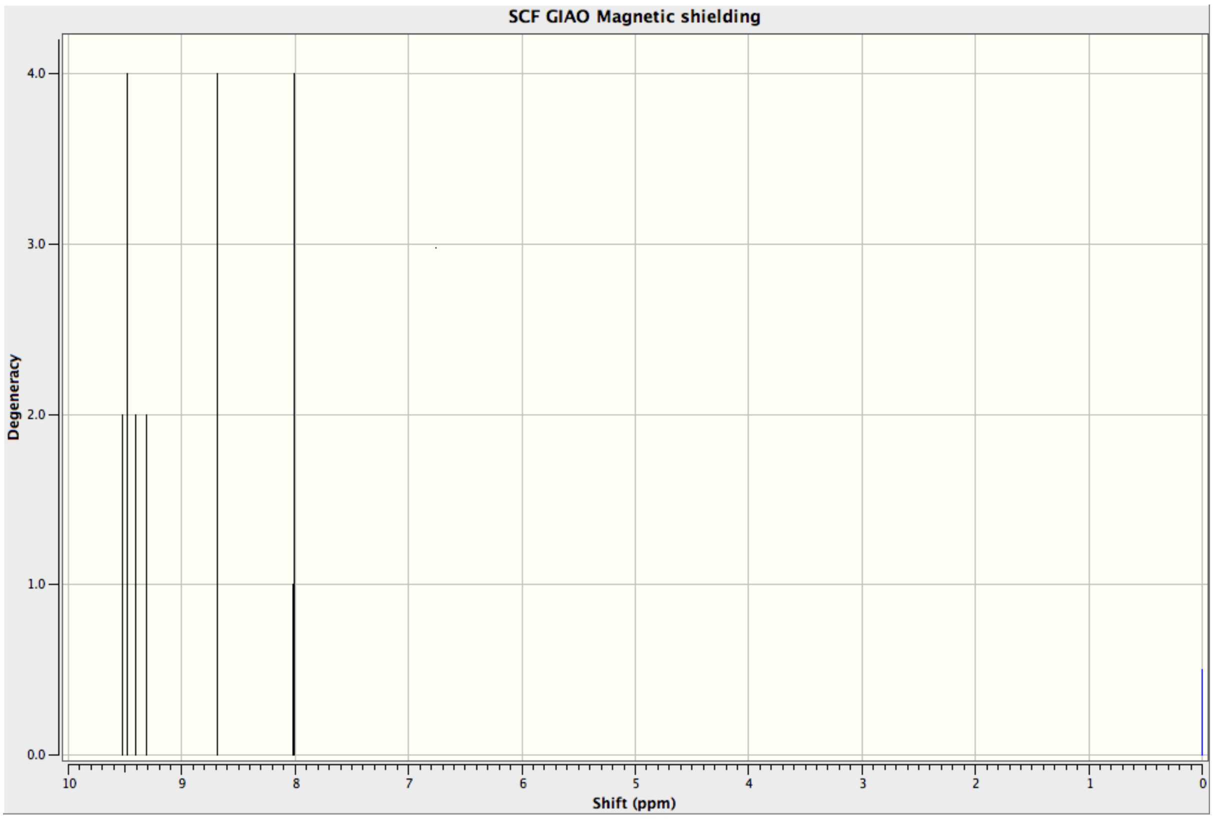

Supplementary Figure 9. Calculated 1H NMR spectrum of **DBATT**.

Spectral appendix (^{1}H & ^{13}C NMR)

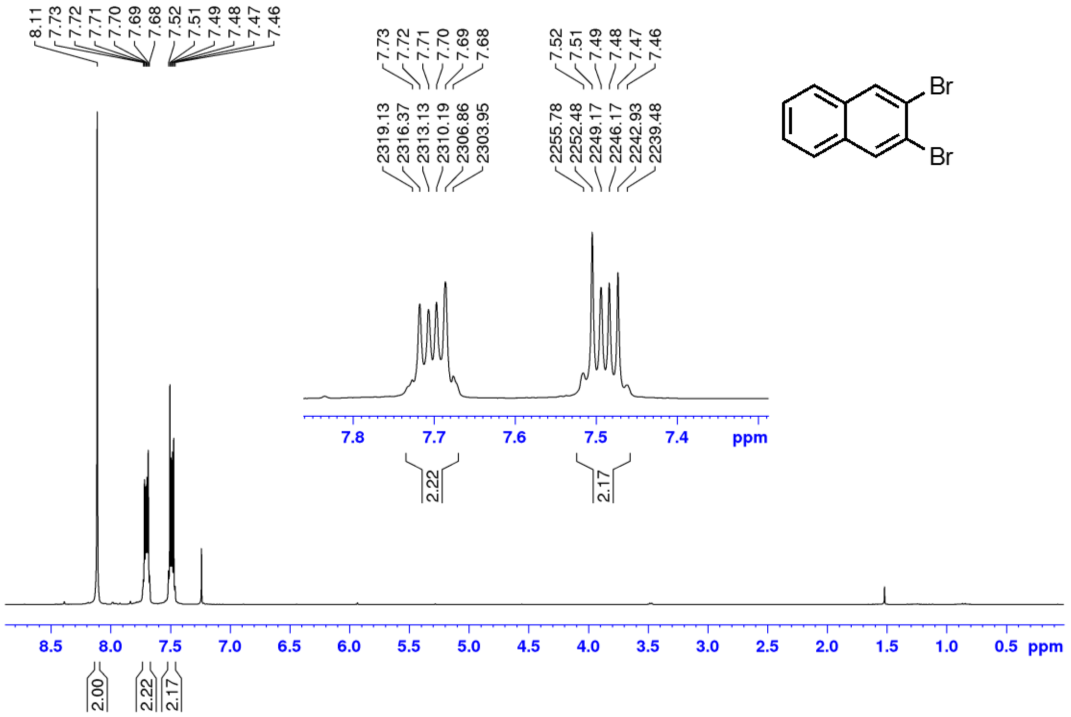

Supplementary Figure 10. ^{1}H NMR (300 MHz, $CDCl_3$, 293 K) spectrum of 2,3-dibromonaphthalene (**S1**).

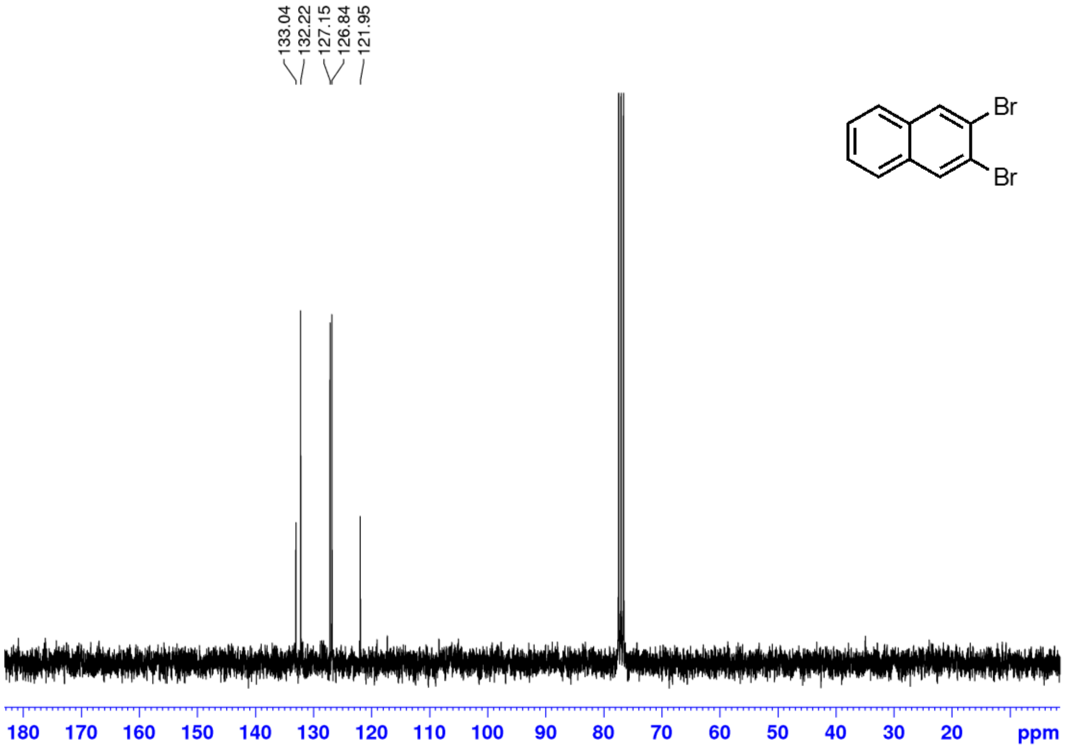

Supplementary Figure 11. ^{13}C NMR (75 MHz, $CDCl_3$, 293 K) spectrum of 2,3-dibromonaphthalene (**S1**).

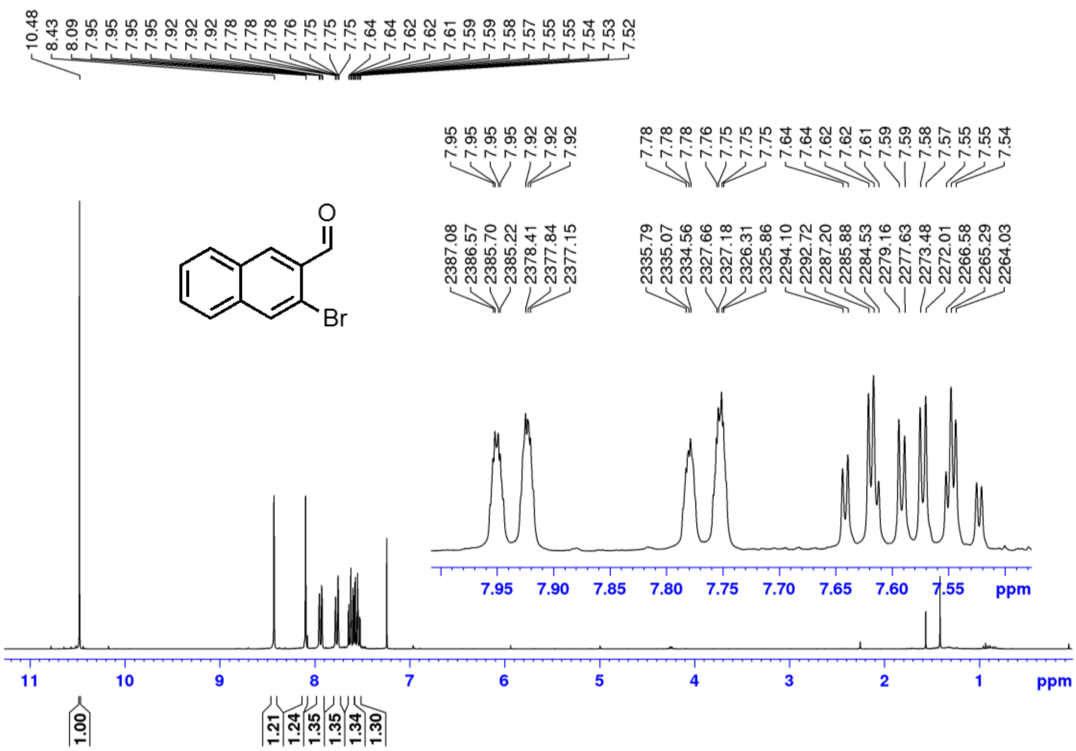

Supplementary Figure 12. ^{1}H NMR (300 MHz, $CDCl_3$, 293 K) spectrum of 2-bromo-3-formylnaphthalene (**S2**).

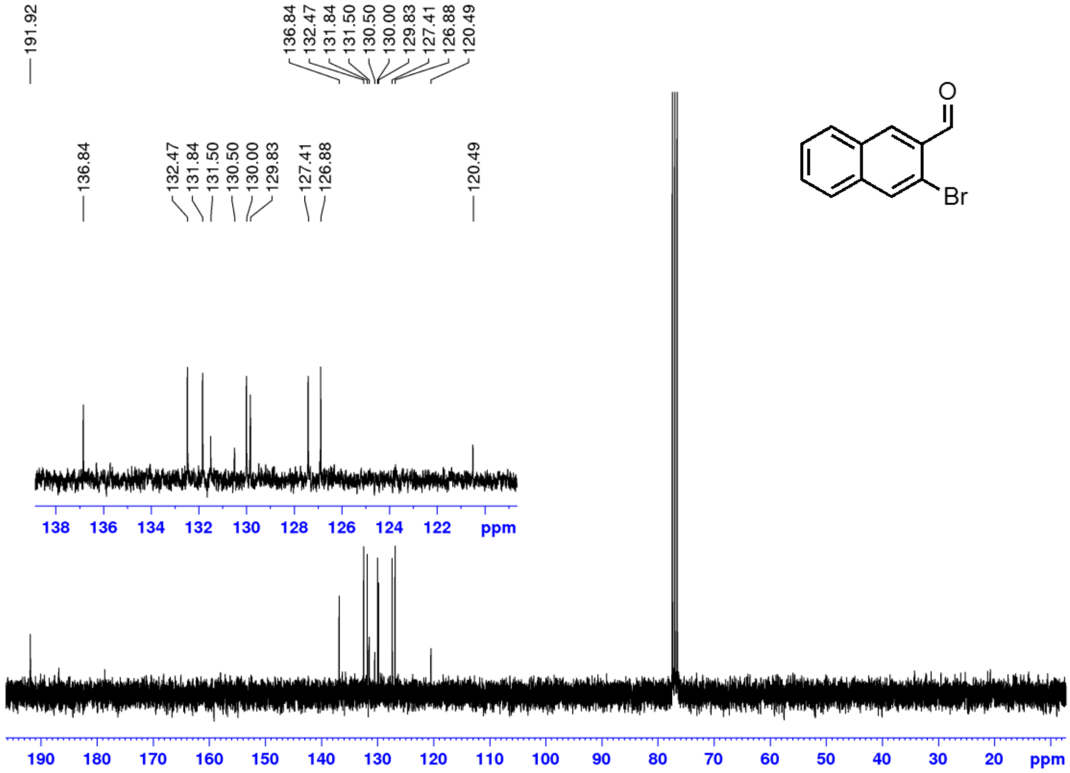

Supplementary Figure 13. ^{13}C NMR (75 MHz, $CDCl_3$, 293 K) spectrum of 2-bromo-3-formylnaphthalene (**S2**).

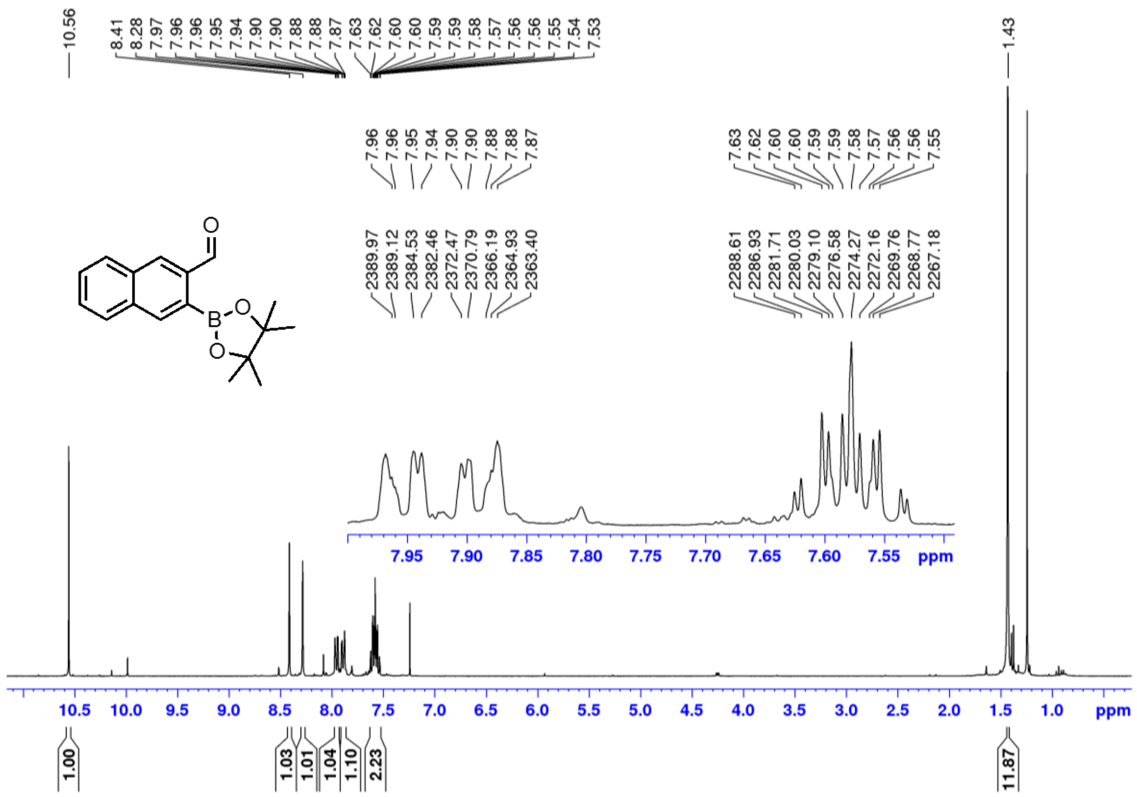

Supplementary Figure 14. ^{1}H NMR (300 MHz, $CDCl_3$, 293 K) spectrum of 3-formylnaphthalene-2-boronic acid pinacol ester (**5**).

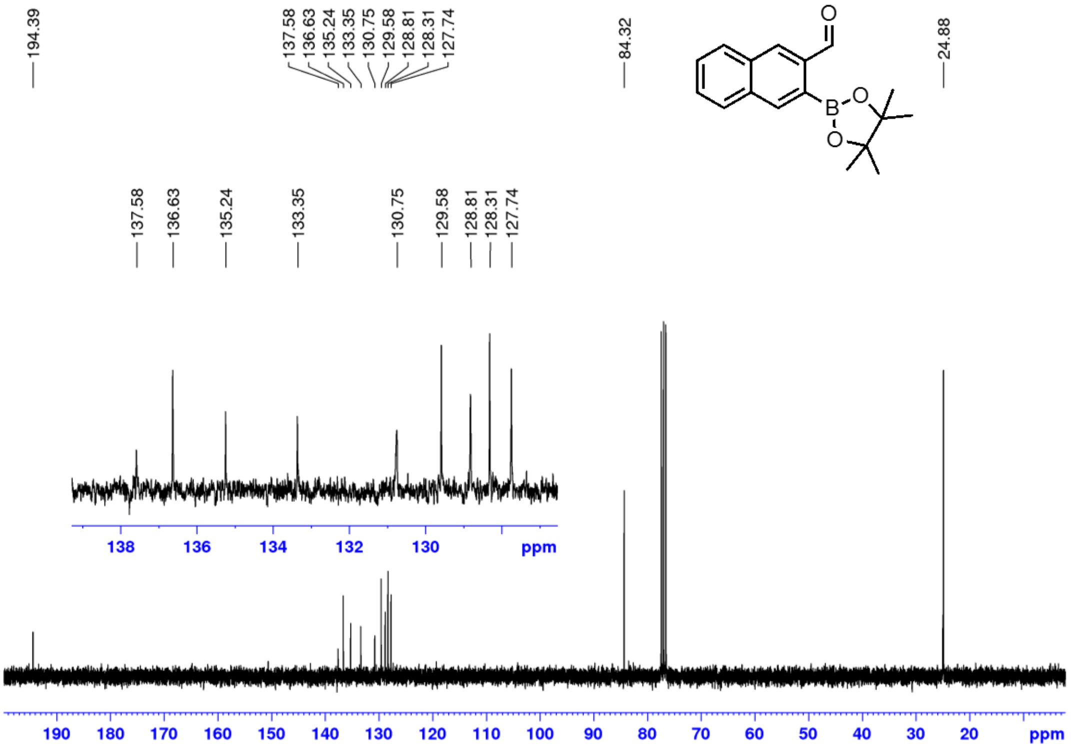

Supplementary Figure 15. ^{13}C NMR (75 MHz, $CDCl_3$, 293 K) spectrum of 3-formylnaphthalene-2-boronic acid pinacol ester (**5**).

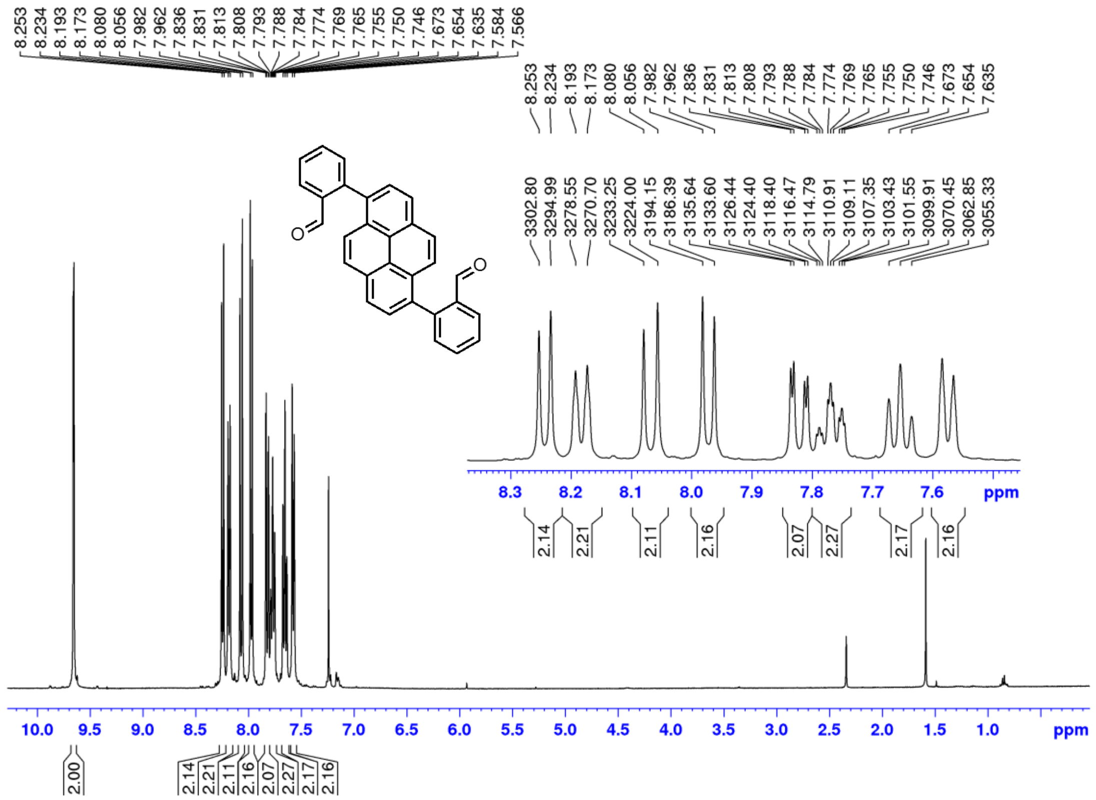

Supplementary Figure 16. ^{1}H NMR (400 MHz, $CDCl_3$, 293 K) spectrum of 1,6-bis(2-formylphenyl)pyrene (**3**).

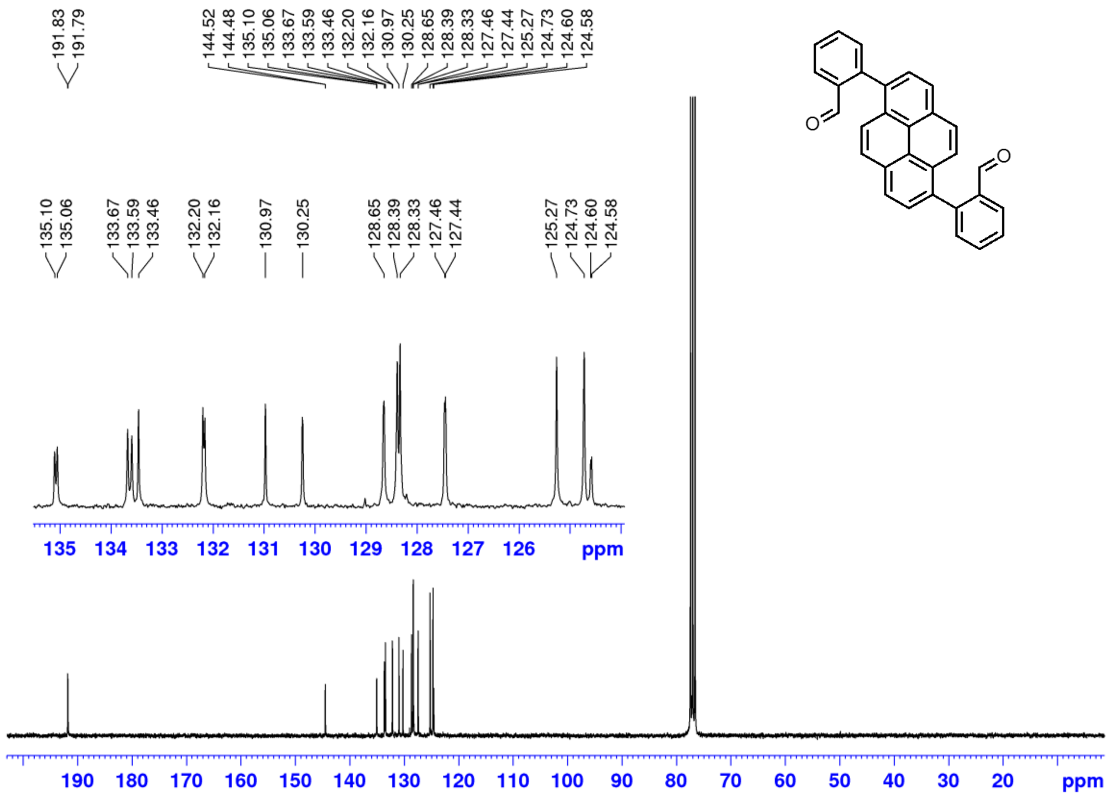

Supplementary Figure 17. ^{13}C NMR (75 MHz, $CDCl_3$, 293 K) of 1,6-bis(2-formylphenyl)pyrene (**3**).

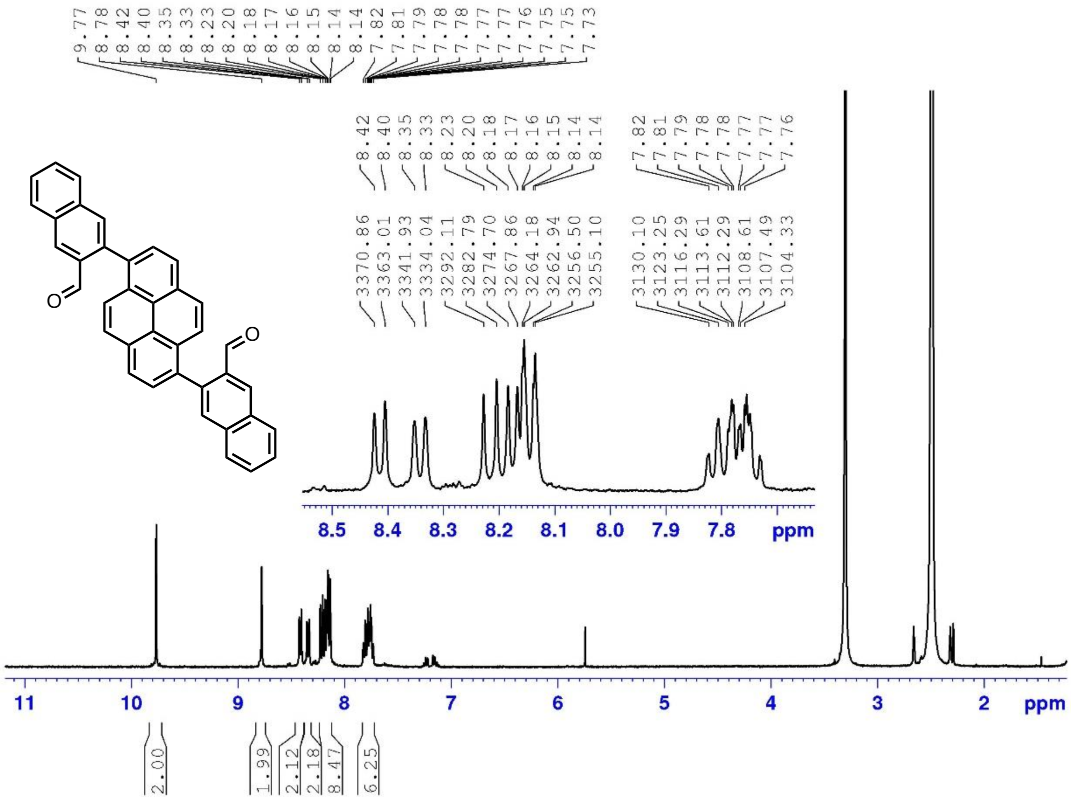

Supplementary Figure 18. ^{1}H NMR (400 MHz, DMSO, 293 K) of 1,6-bis(3-formylnaphthyl)pyrene **(4)**.

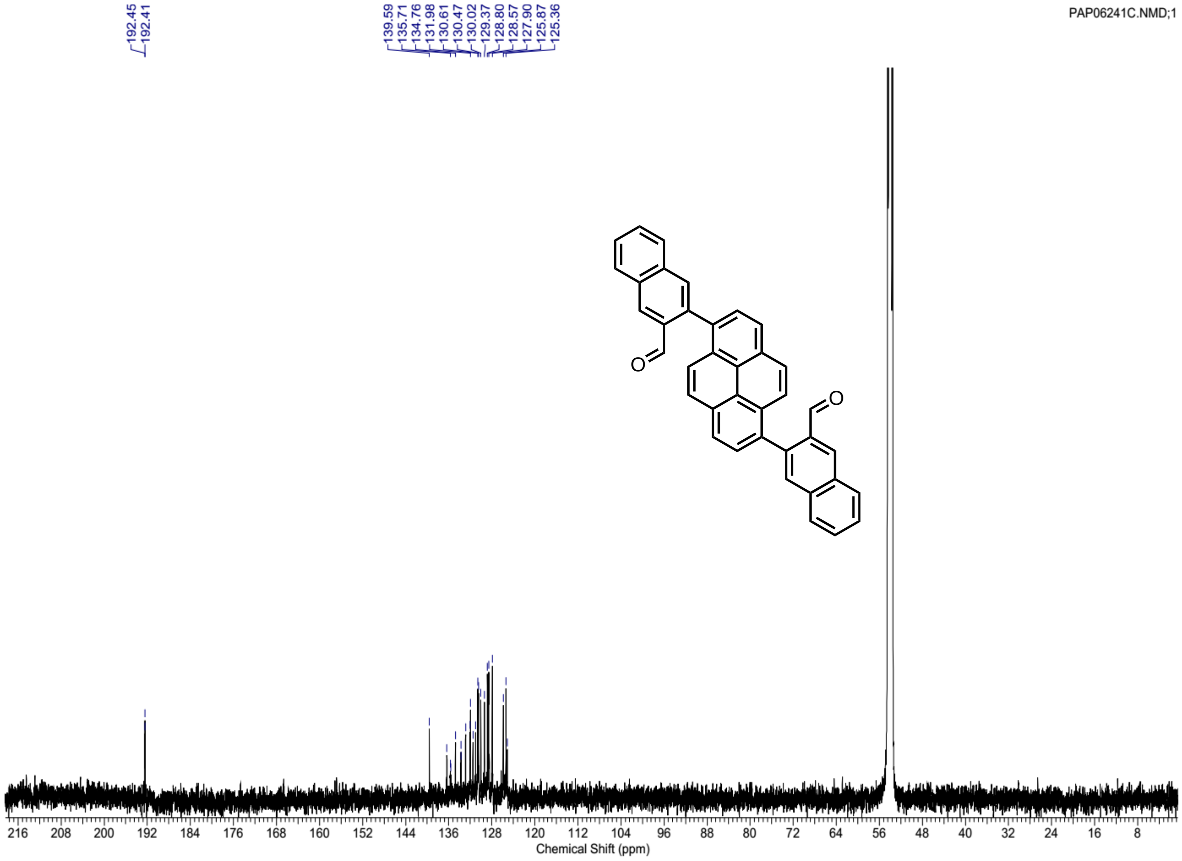

Supplementary Figure 19. ^{13}C NMR (125 MHz, CD_2Cl_2, 293 K) of 1,6-bis(3-formylnaphthyl)pyrene **(4)**

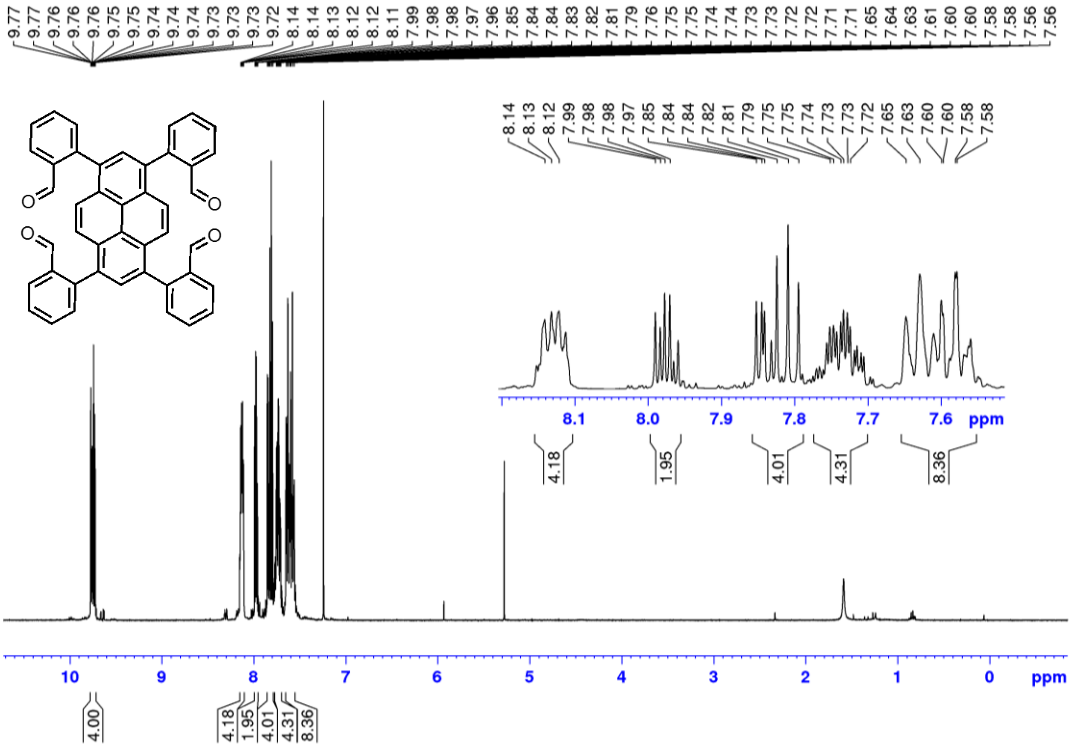

Supplementary Figure 20. ^{1}H NMR (400 MHz, $CDCl_3$, 293 K) 1,3,6,8-tetrakis(2-formylphenyl)pyrene (**8**).

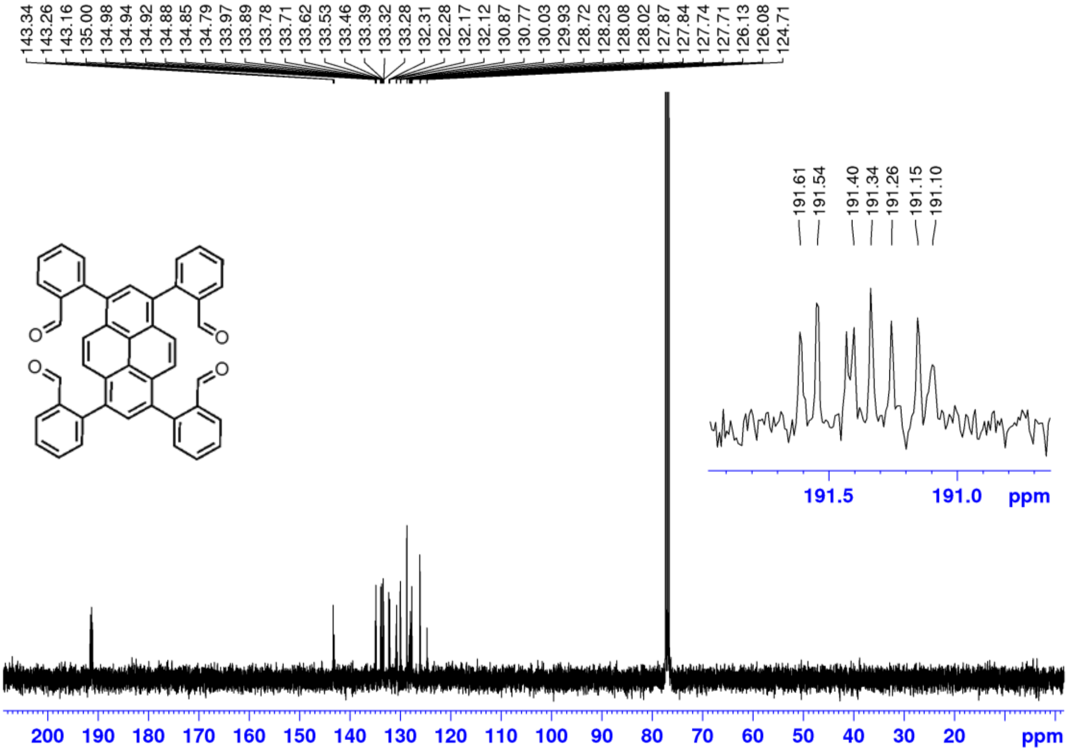

Supplementary Figure 21. ^{13}C NMR (100 MHz, $CDCl_3$, 293 K) 1,3,6,8-tetrakis(2-formylphenyl)pyrene (**8**).

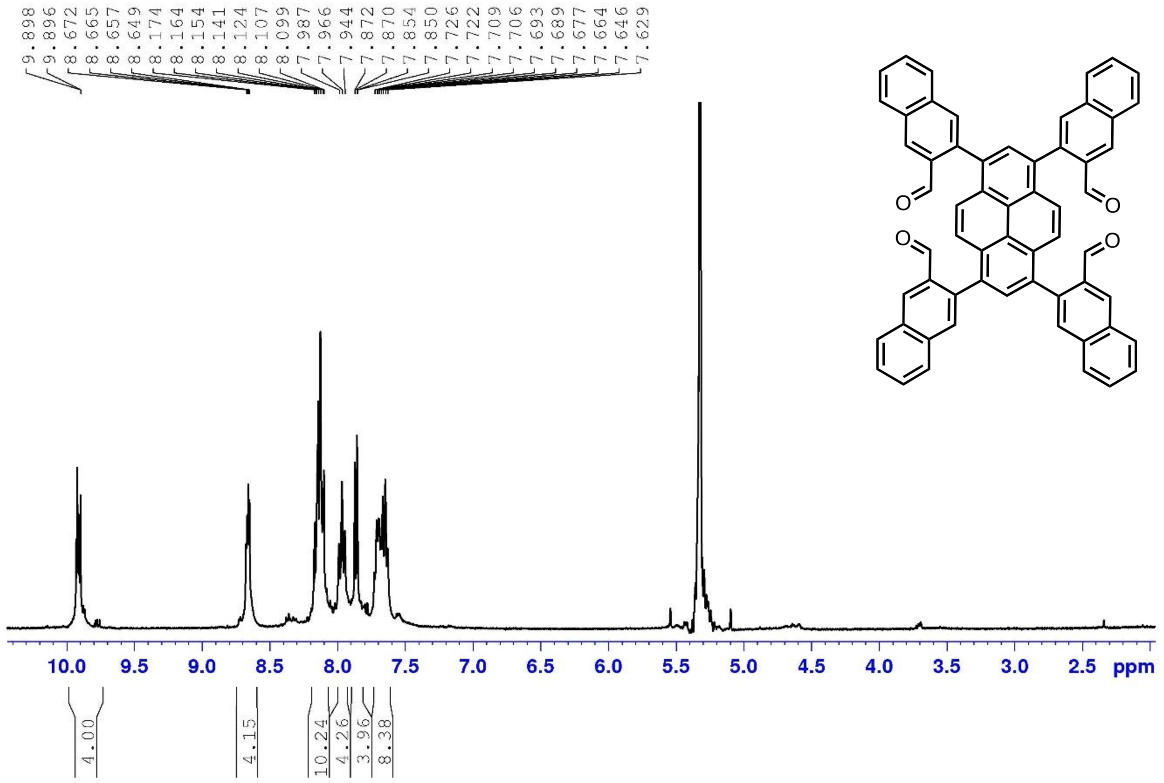

Supplementary Figure 22. ^{1}H NMR (400 MHz, CD_2Cl_2, 293 K) of 1,3,6,8-tetrakis(3-formylnaphthyl)pyrene (**9**).

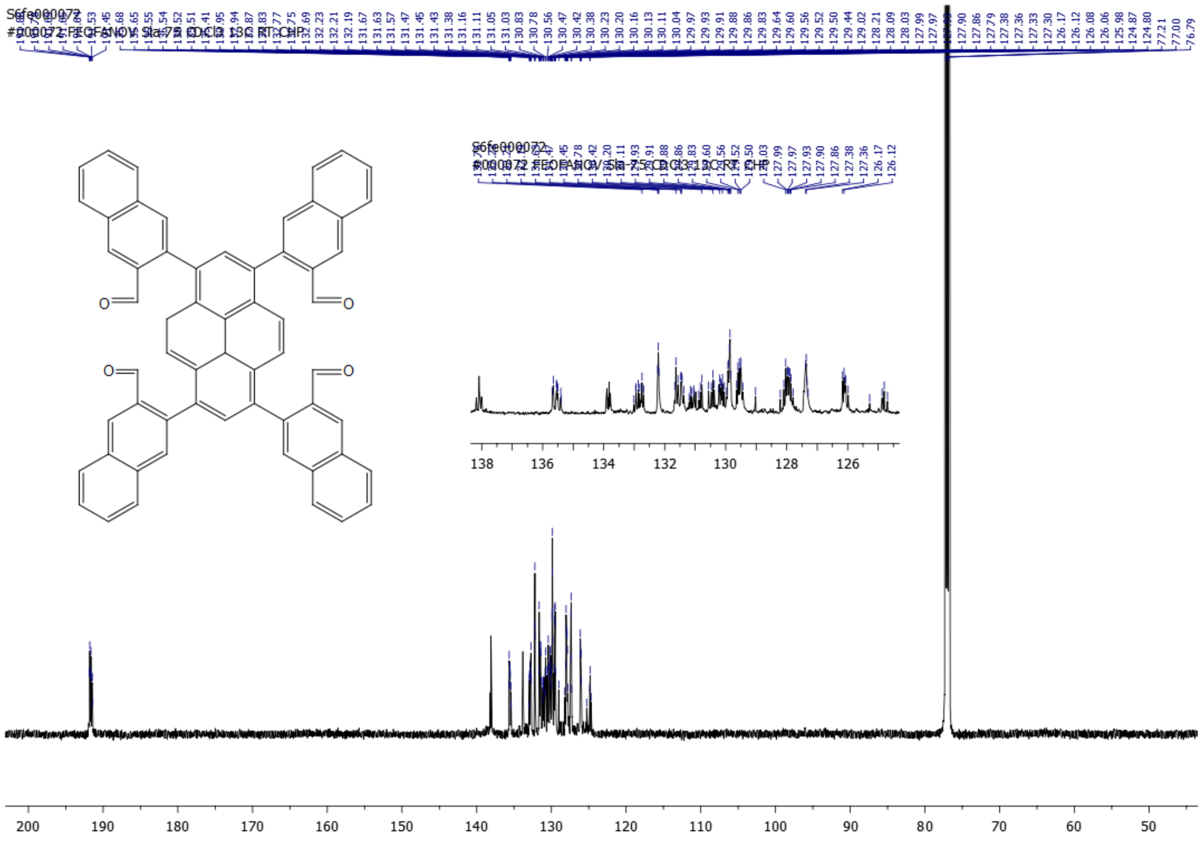

Supplementary Figure 23. ^{13}C NMR (151 MHz, $CDCl_3$, 293 K) of 1,3,6,8-tetrakis(3-formylnaphthyl)pyrene (**9**).

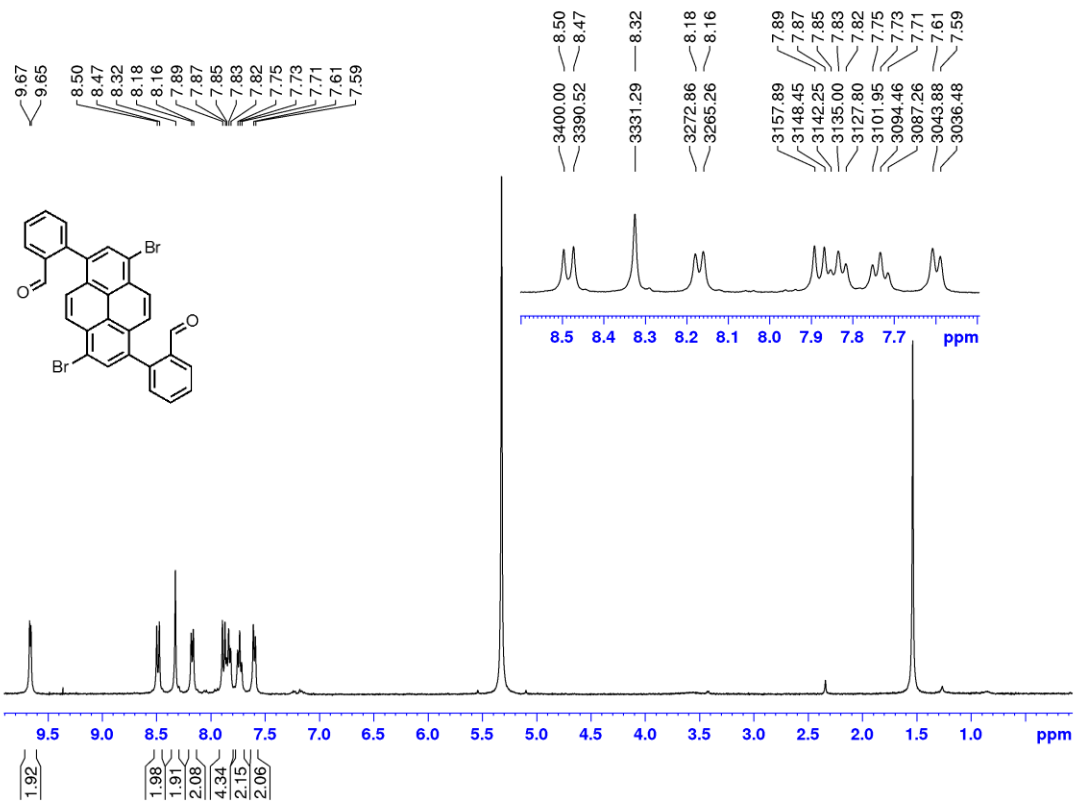

Supplementary Figure 24. ^{1}H NMR (400 MHz, CD_2Cl_2, 293 K) of 1,6-dibromo-3,8-bis(2-formylphenyl)pyrene (**6**).

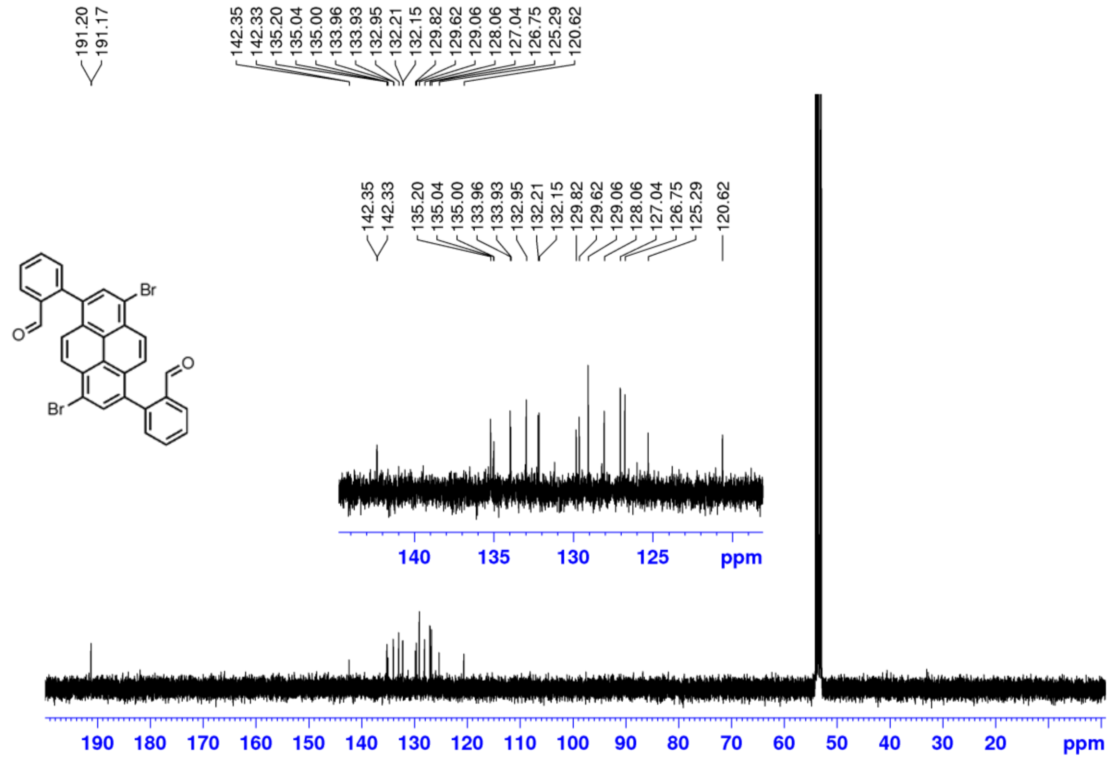

Supplementary Figure 25. ^{13}C NMR (100 MHz, CD_2Cl_2, 293K) of 1,6-dibromo-3,8-bis(2-formylphenyl)pyrene (**6**).

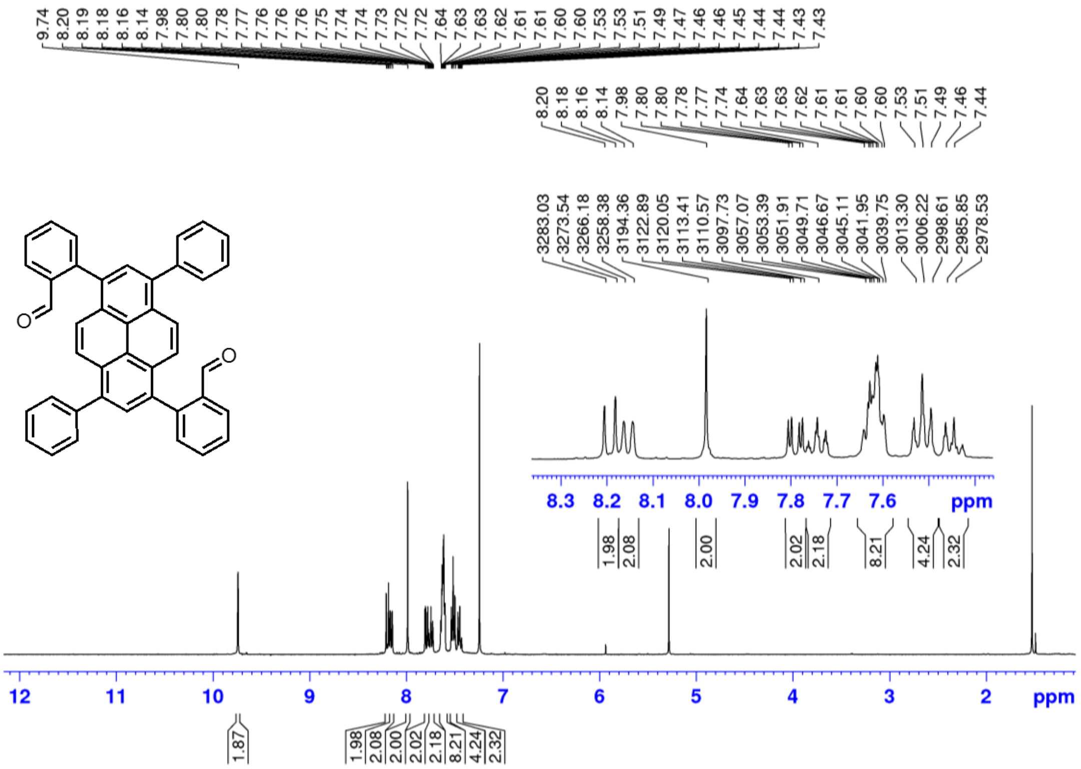

Supplementary Figure 26. ^{1}H NMR (400 MHz, CD_2Cl_2, 293 K) of 1,6-bis(2-formylphenyl)-3,8-diphenylpyrene (**3a**).

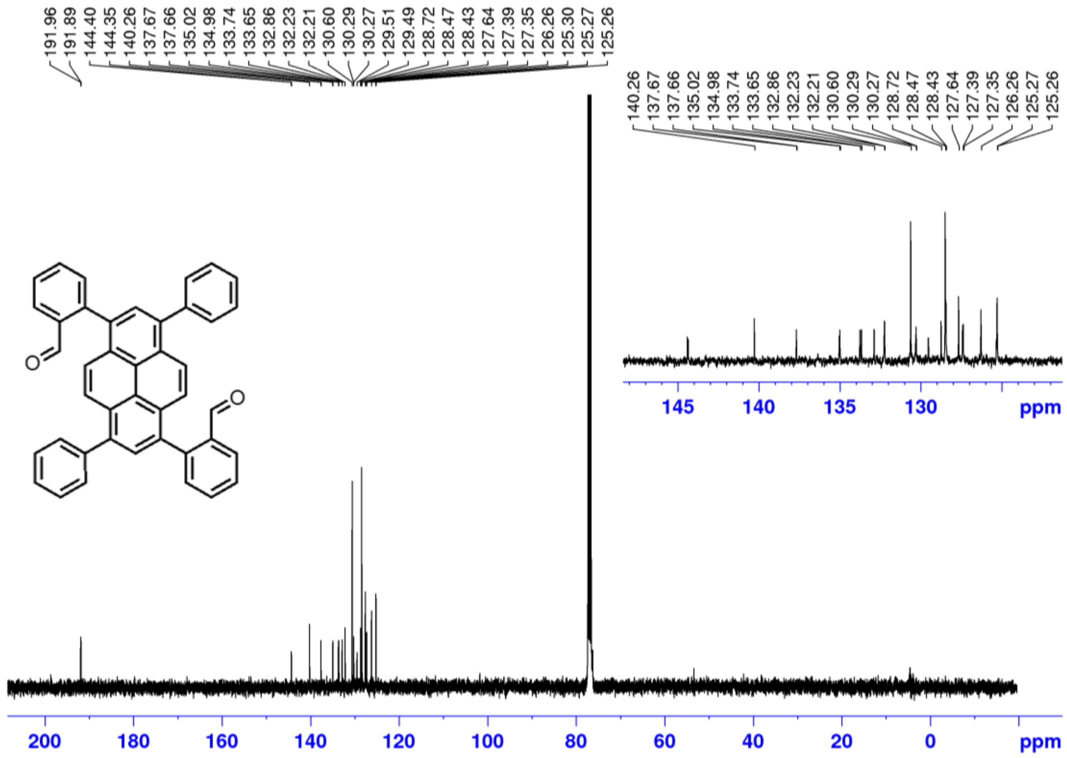

Supplementary Figure 27. ^{13}C NMR (100 MHz, CD_2Cl_2, 293 K) of 1,6-bis(2-formylphenyl)-3,8-diphenylpyrene (**3a**).

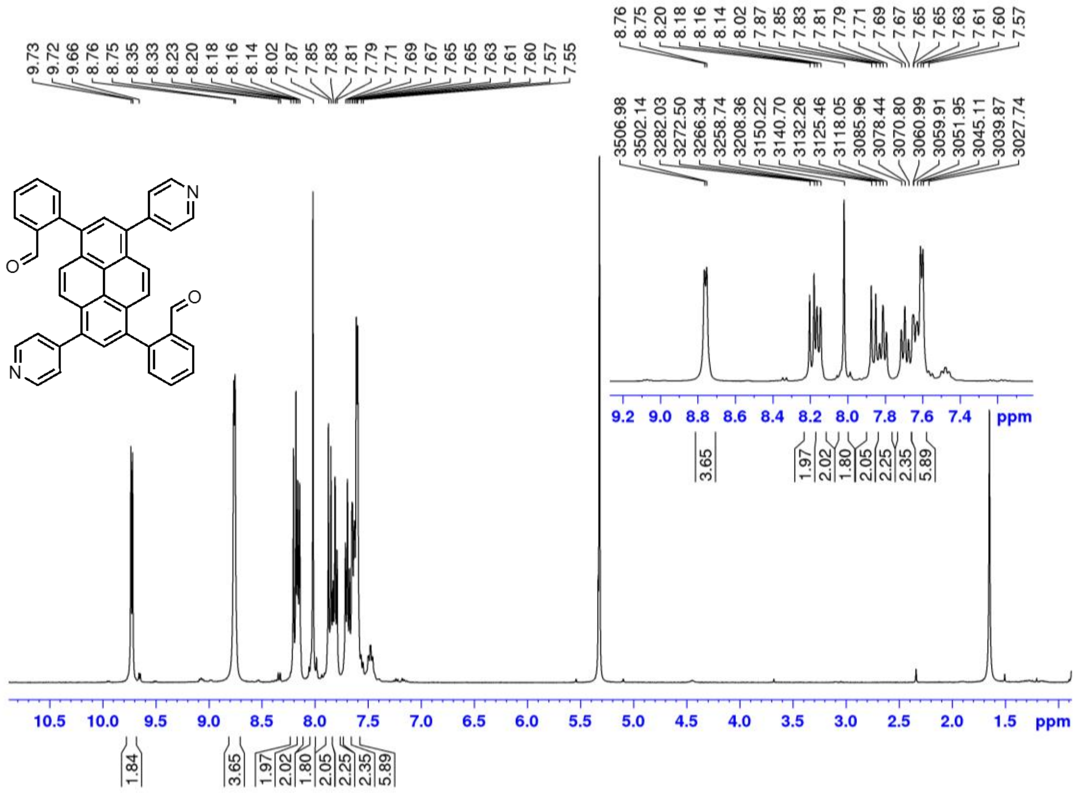

Supplementary Figure 28. ^{1}H NMR (400 MHz, CD_2Cl_2, 293 K) of 1,6-bis(2-formylphenyl)-3,8-bis(4-pyridyl)pyrene (**S5**).

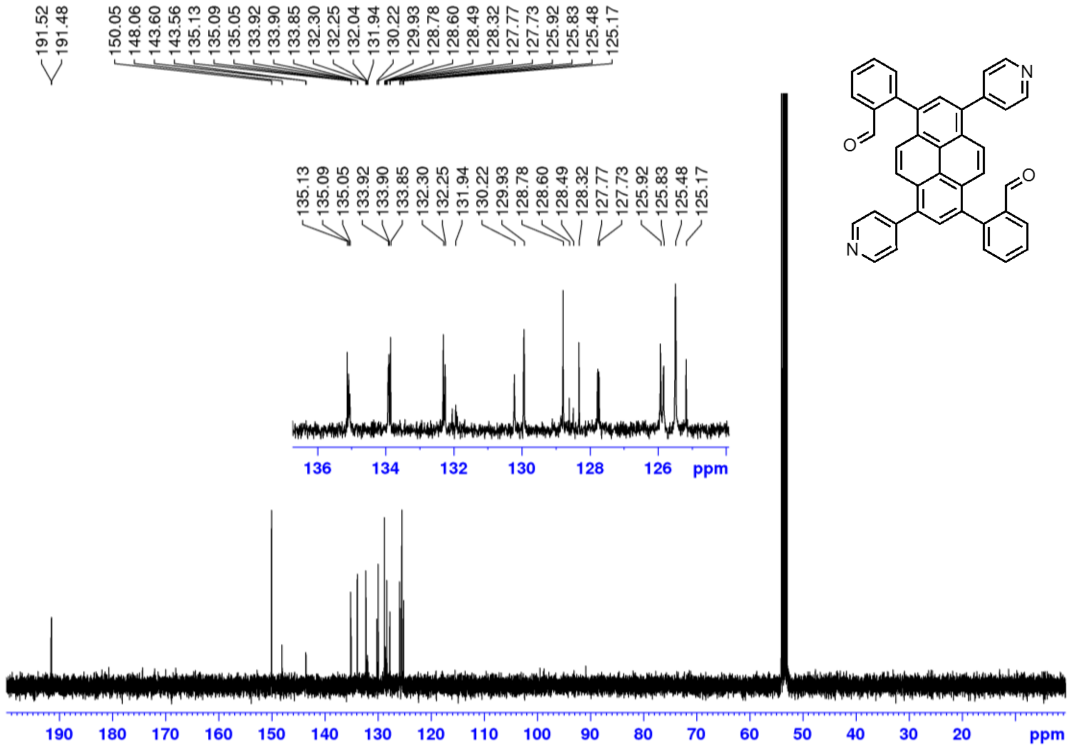

Supplementary Figure 29. ^{13}C NMR (100 MHz, CD_2Cl_2, 293 K) of 1,6-bis(2-formylphenyl)-3,8-bis(4-pyridyl)pyrene (**S5**).

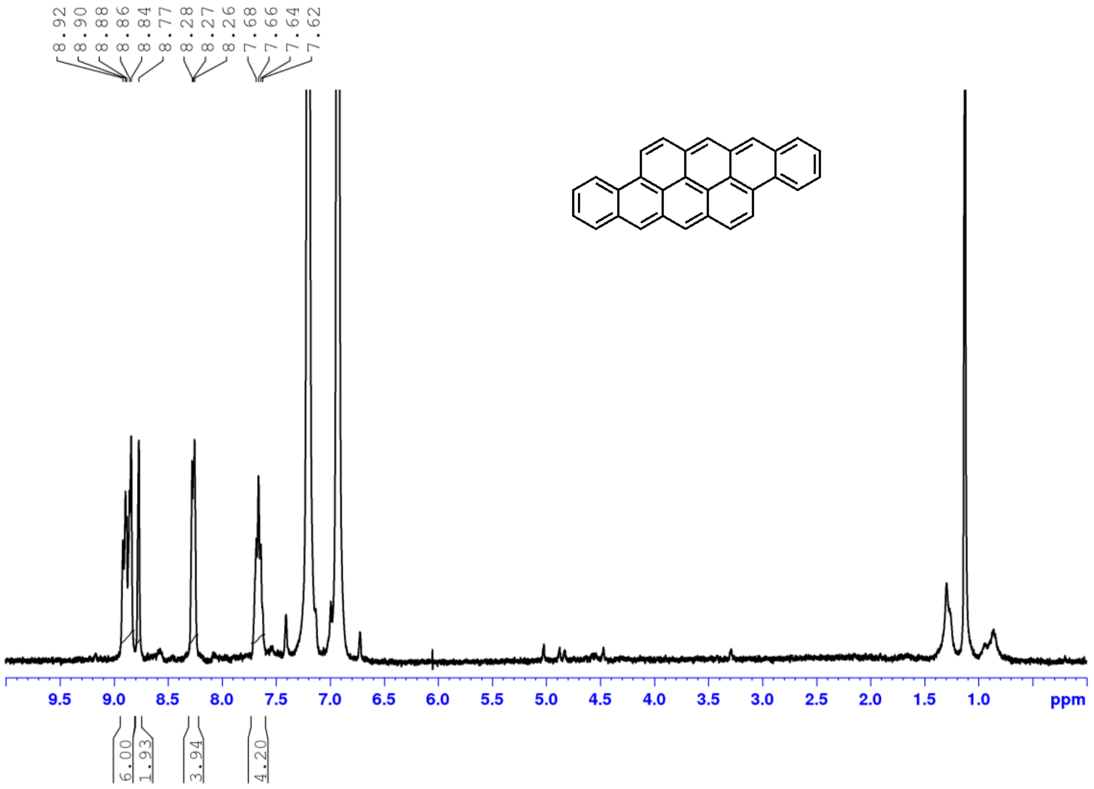

Supplementary Figure 30. ^{1}H NMR (400 MHz, *o*-DCB-D$_4$, 373 K) of **DBATT**.

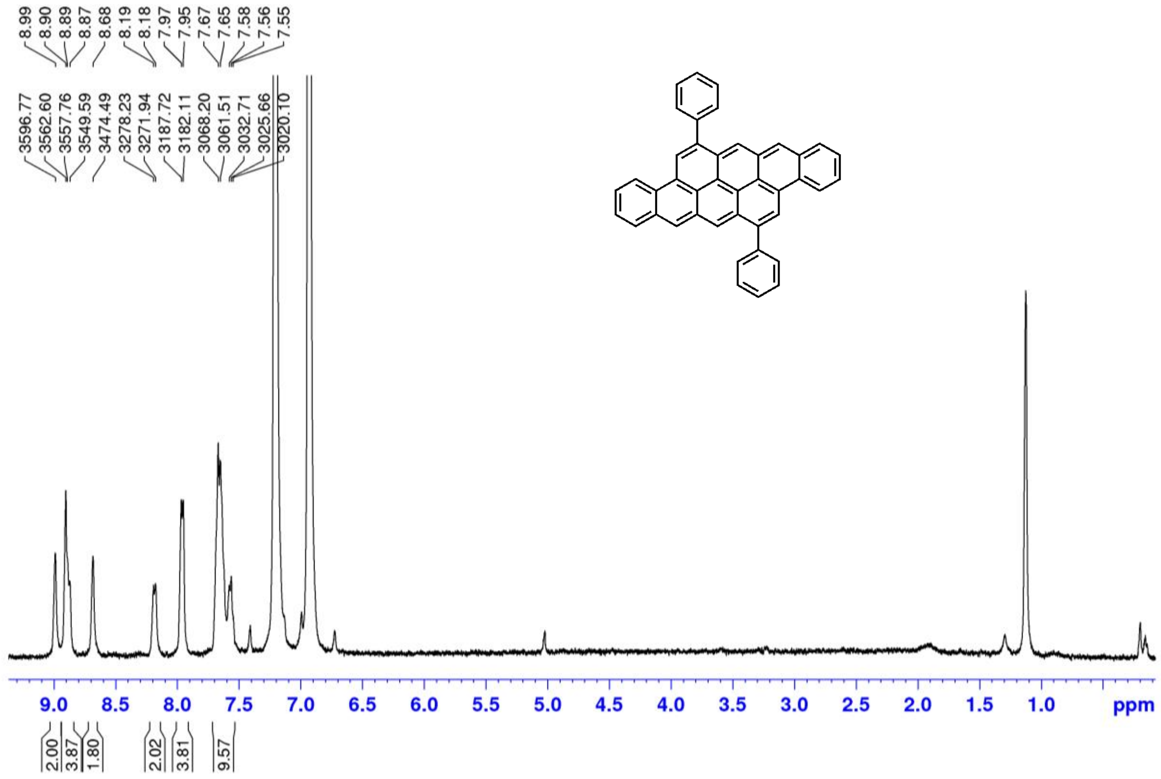

Supplementary Figure 31. ^{1}H NMR (400 MHz, *o*-DCB-D$_4$, 373 K) of 6,14-bisphenyl-2.3,8.9-dibenzanthanthrene (**bPh-DBATT**).

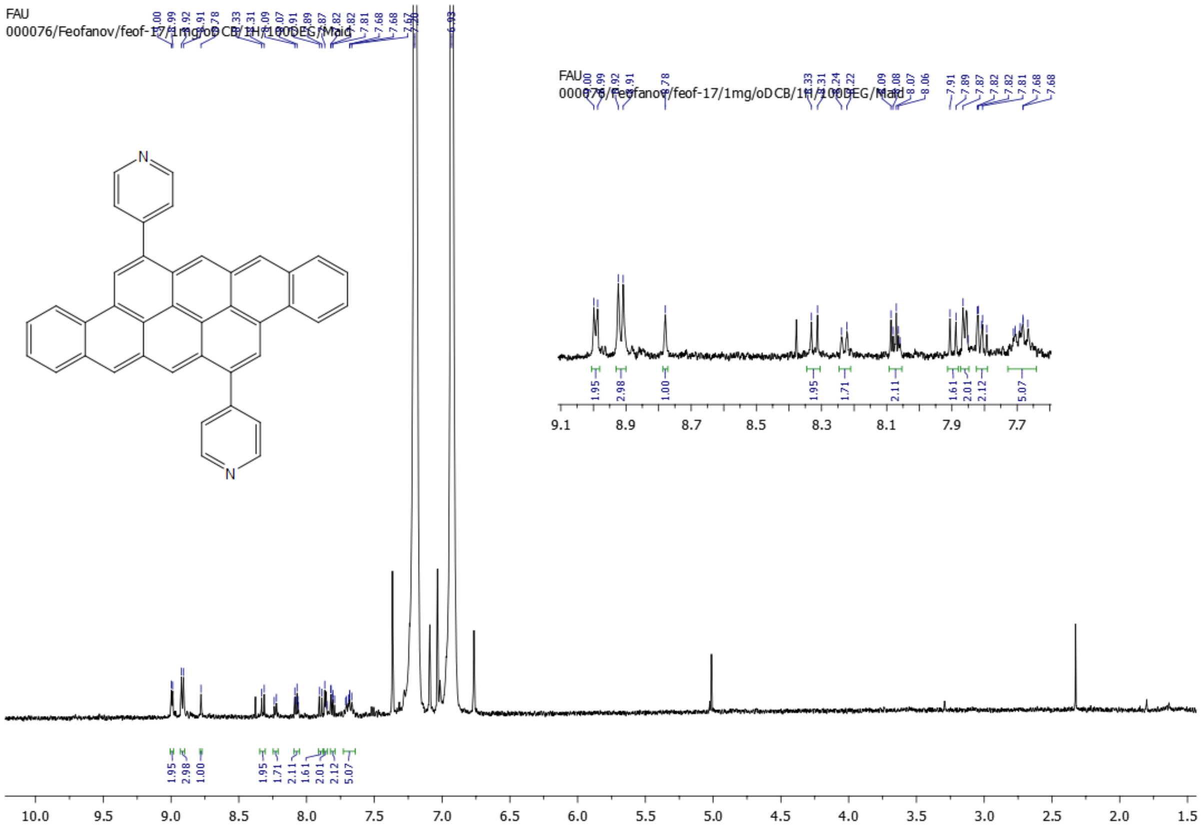

Supplementary Figure 32. ^{1}H NMR (500 MHz, *o*-DCB-D_4, 393 K) of 6,14-bis(4-pyridinyl)-2.3,8.9-dibenzanthanthrene (**S7**).

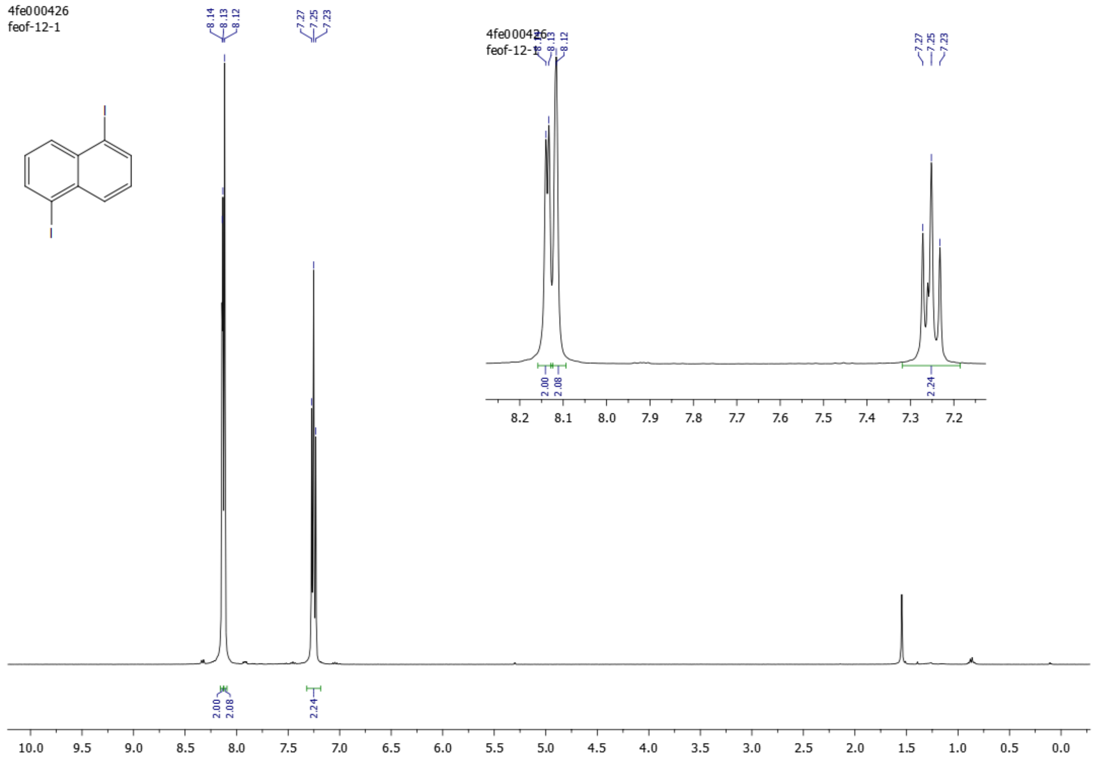

Supplementary Figure 33. ^{1}H NMR (400 MHz, $CDCl_3$, 293 K) of 1,5-diiodonaphthalene (**S3**).

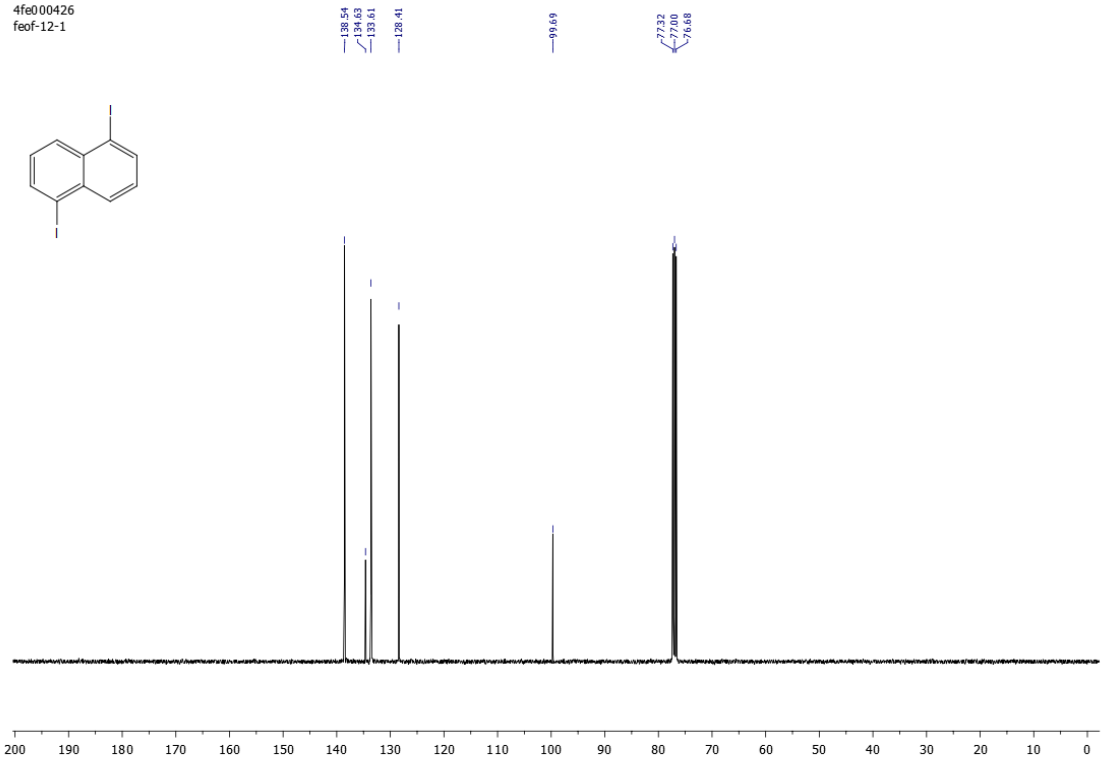

Supplementary Figure 34. ^{13}C NMR (100 MHz, $CDCl_3$, 293 K) of 1,5-diiodonaphthalene (**S3**).

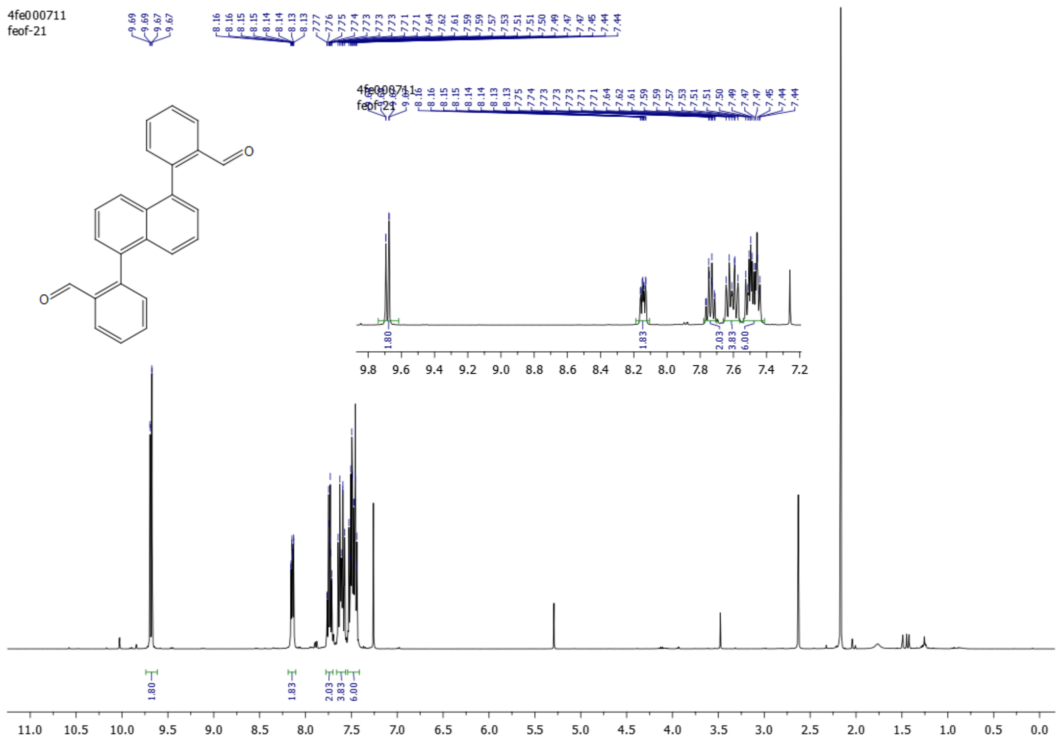

Supplementary Figure 35. ^{1}H NMR (400 MHz, $CDCl_3$, 293 K) of 2,2'-(naphthalene-1,5-diyl)dibenzaldehyde (**12**).

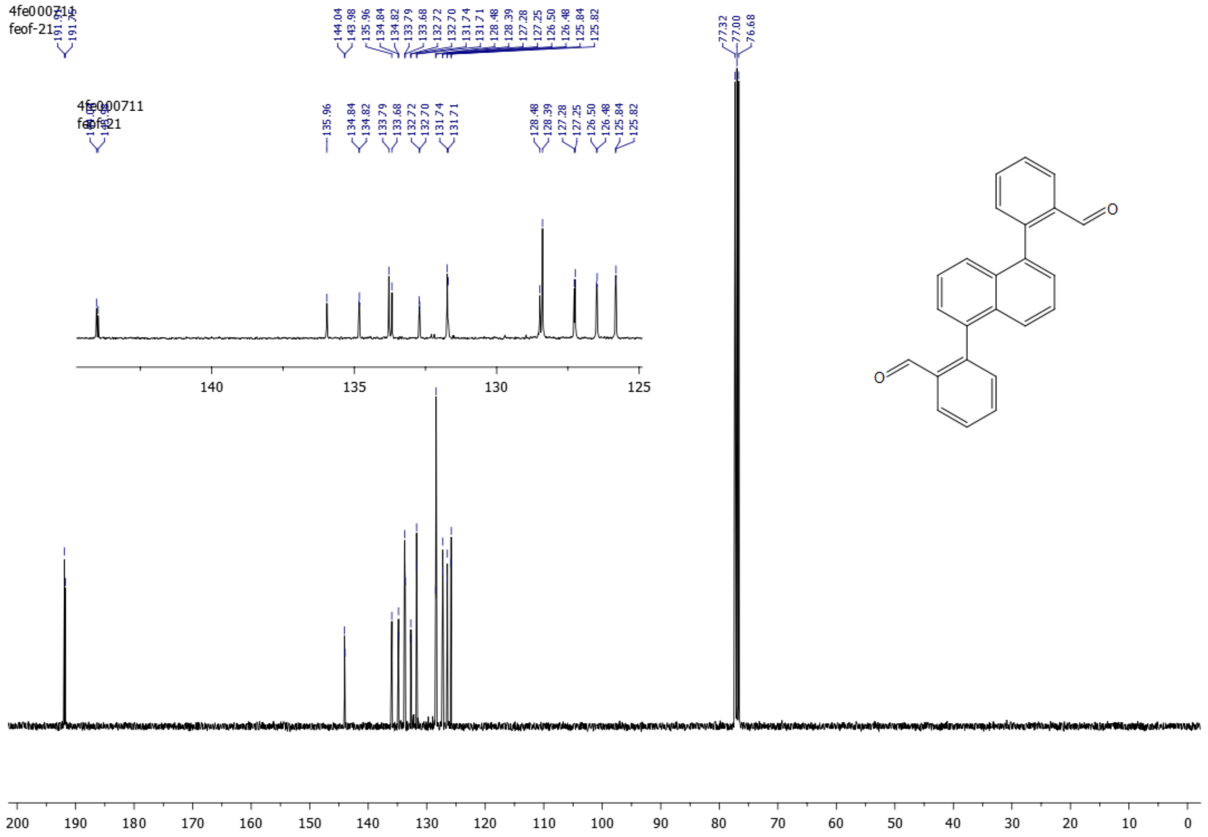

Supplementary Figure 36. ^{13}C NMR (101 MHz, $CDCl_3$, 293 K) of 2,2'-(naphthalene-1,5-diyl)dibenzaldehyde (**12**).

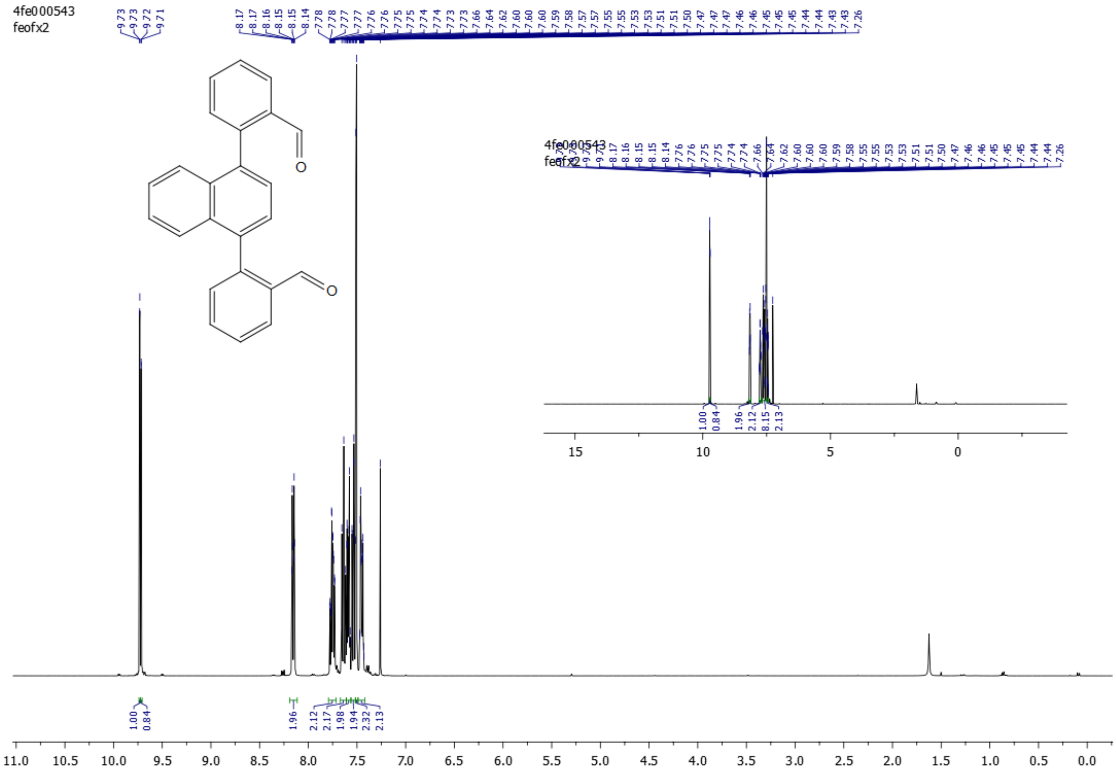

Supplementary Figure 37. ^{1}H NMR (400 MHz, $CDCl_3$, 293 K) of 2,2'-(naphthalene-1,4-diyl)dibenzaldehyde (**10**).

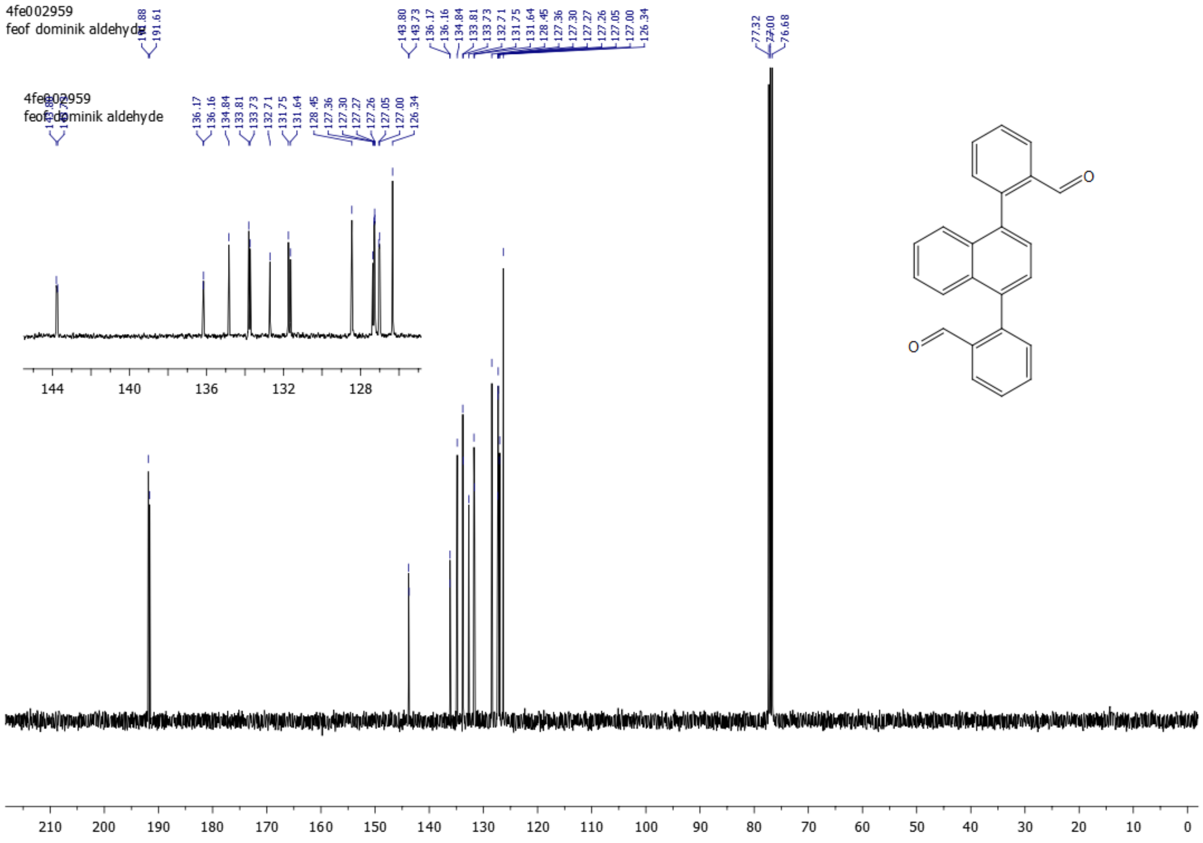

Supplementary Figure 38. ^{13}C NMR (101 MHz, $CDCl_3$, 293 K) of 2,2'-(naphthalene-1,4-diyl)dibenzaldehyde (**10**).

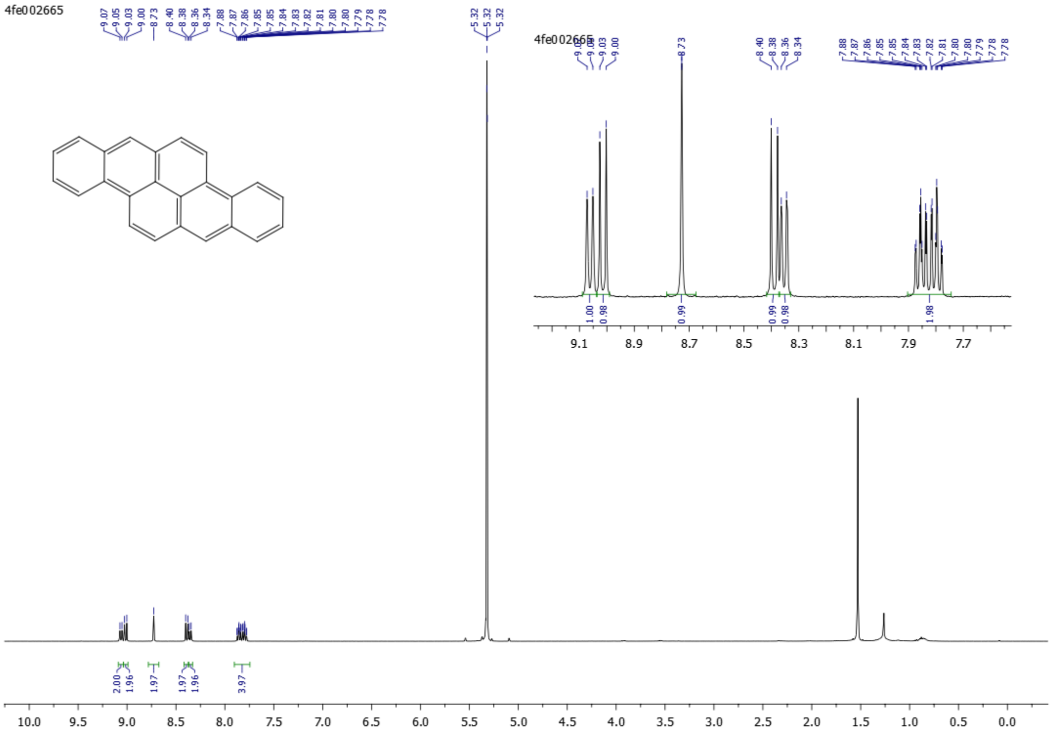

Supplementary Figure 39. ^{1}H NMR (400 MHz, $CDCl_3$, 293 K) of dibenzo[*b,def*]chrysene (**13**).

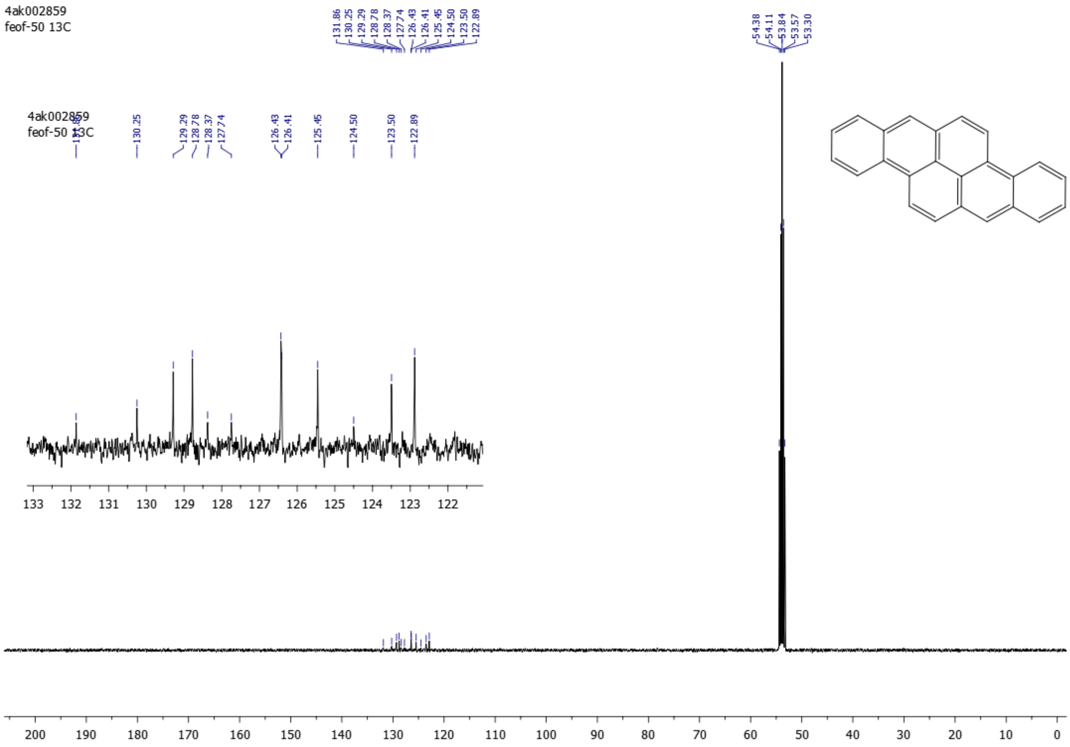

Supplementary Figure 40. ^{13}C NMR (101 MHz, $CDCl_3$, 293 K) of dibenzo[*b,def*]chrysene (**13**).

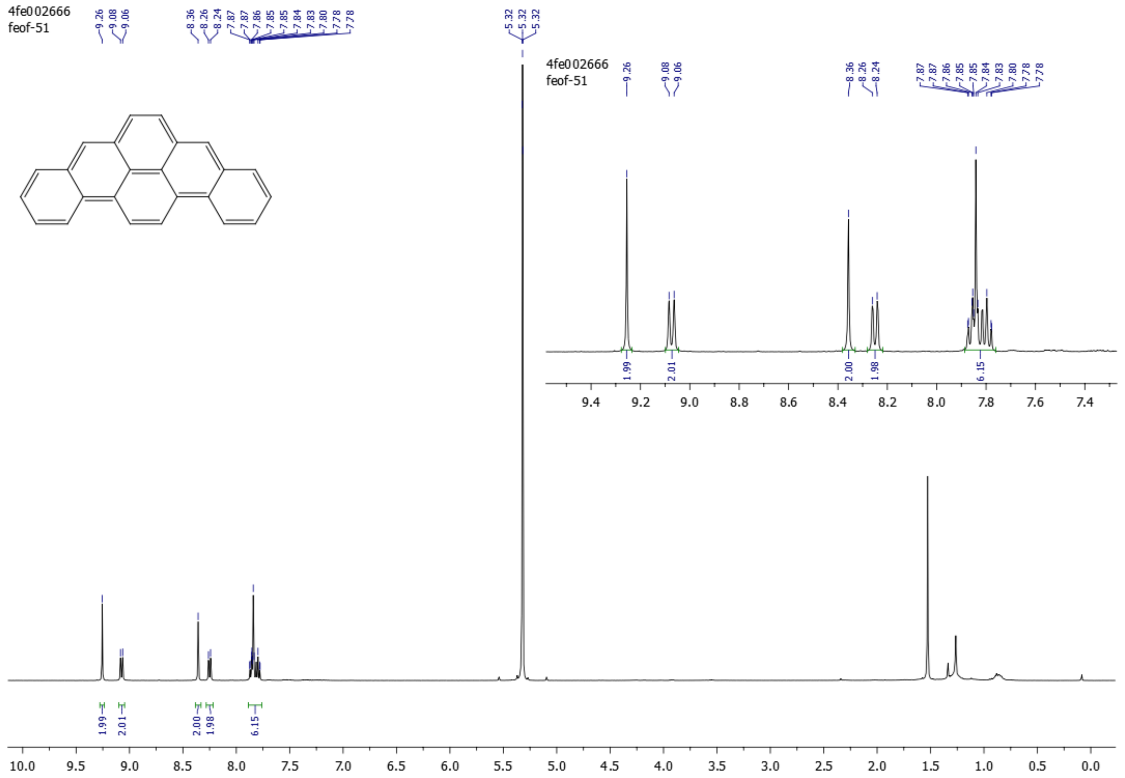

Supplementary Figure 41. ^{1}H NMR (400 MHz, CD_2Cl_2, 293 K) of benzo[*rst*]pentaphene (**11**).

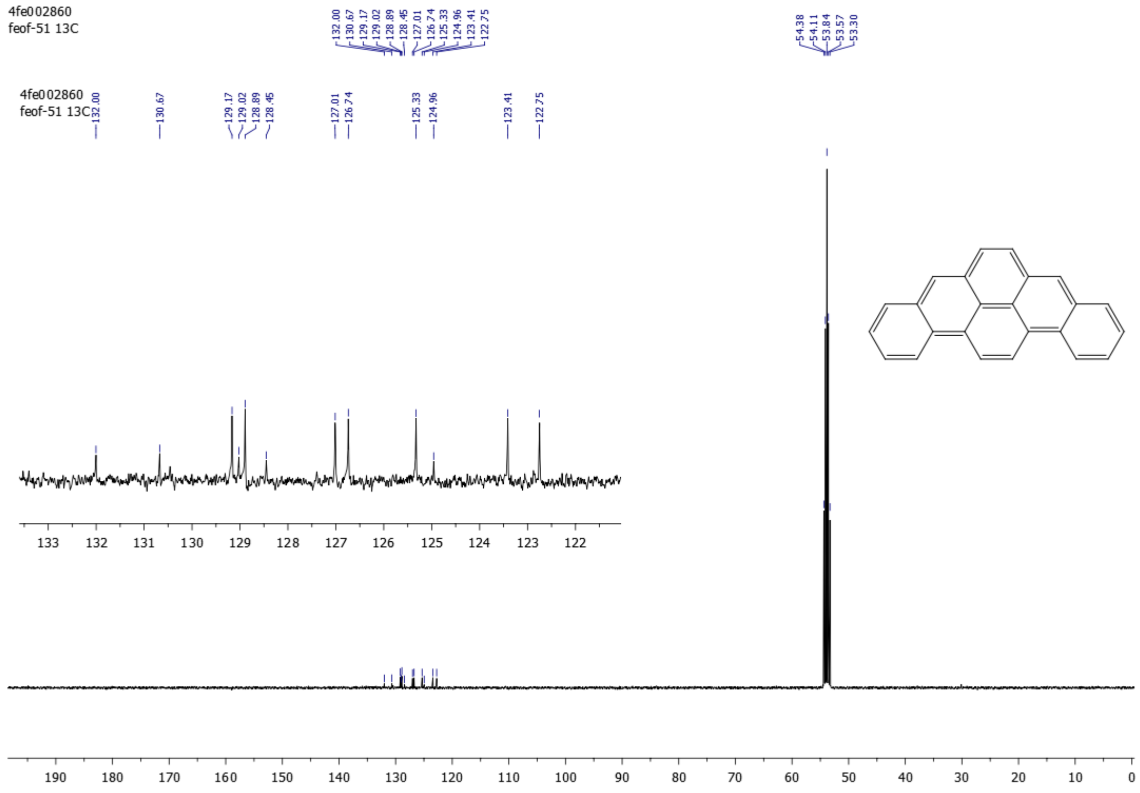

Supplementary Figure 42. ^{13}C NMR (101 MHz, CD_2Cl_2, 293 K) of benzo[*rst*]pentaphene (**11**).

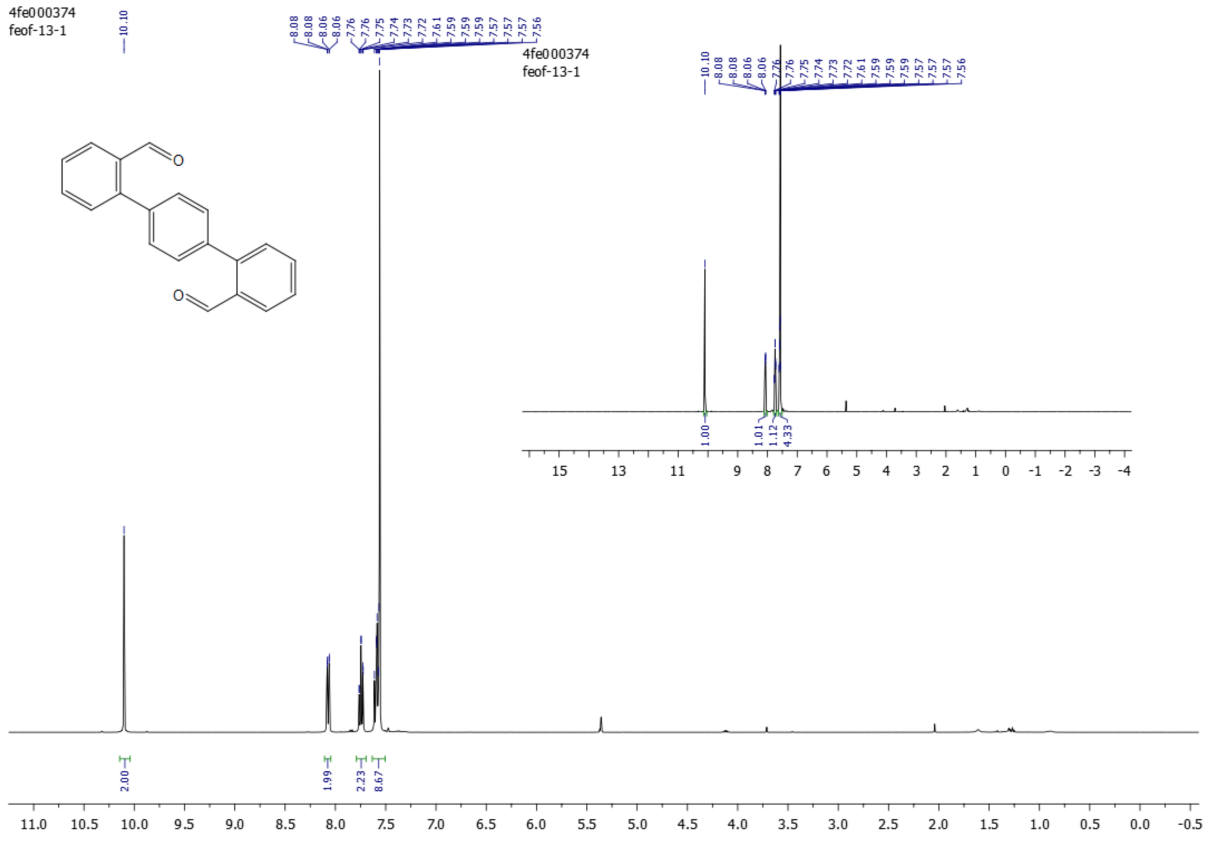

Supplementary Figure 43. ^{1}H NMR (400 MHz, CD_2Cl_2, 293 K) of [1,1':4',1''-terphenyl]-2,2''-dicarbaldehyde (**14**).

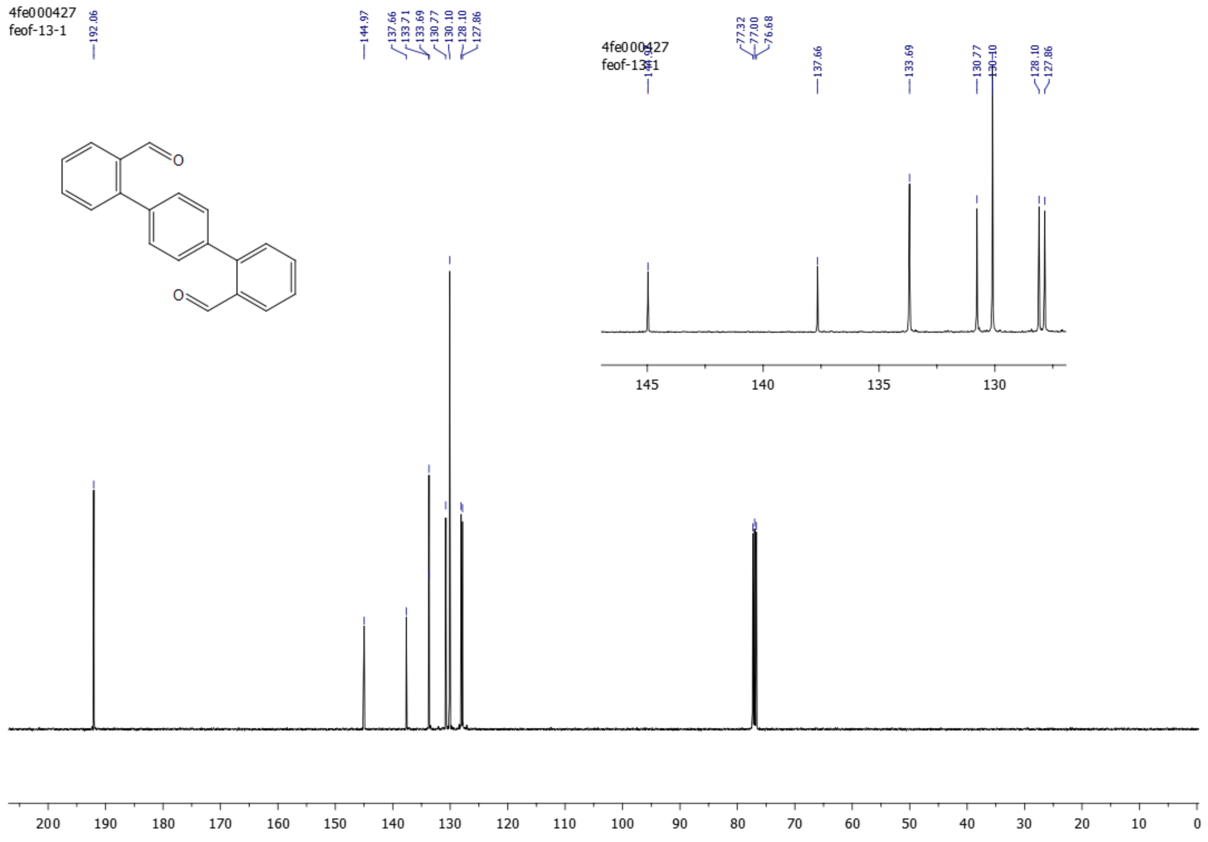

Supplementary Figure 44. ^{13}C NMR (101 MHz, CD_2Cl_2, 293 K) of [1,1':4',1''-terphenyl]-2,2''-dicarbaldehyde (**14**).

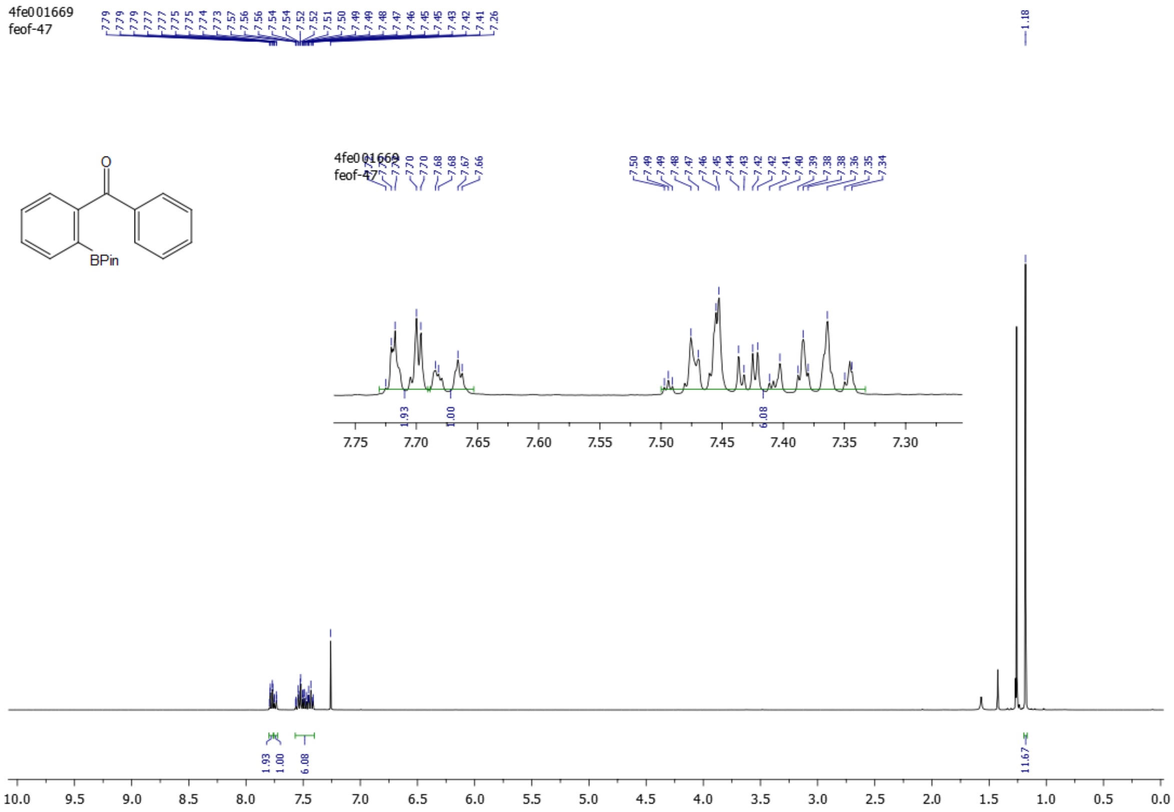

Supplementary Figure 45. ^{1}H NMR (400 MHz, $CDCl_3$, 293 K) of phenyl(2-(4,4,5,5-tetramethyl-1,3,2-dioxaborolan-2-yl)phenyl)methanone (**S4**).

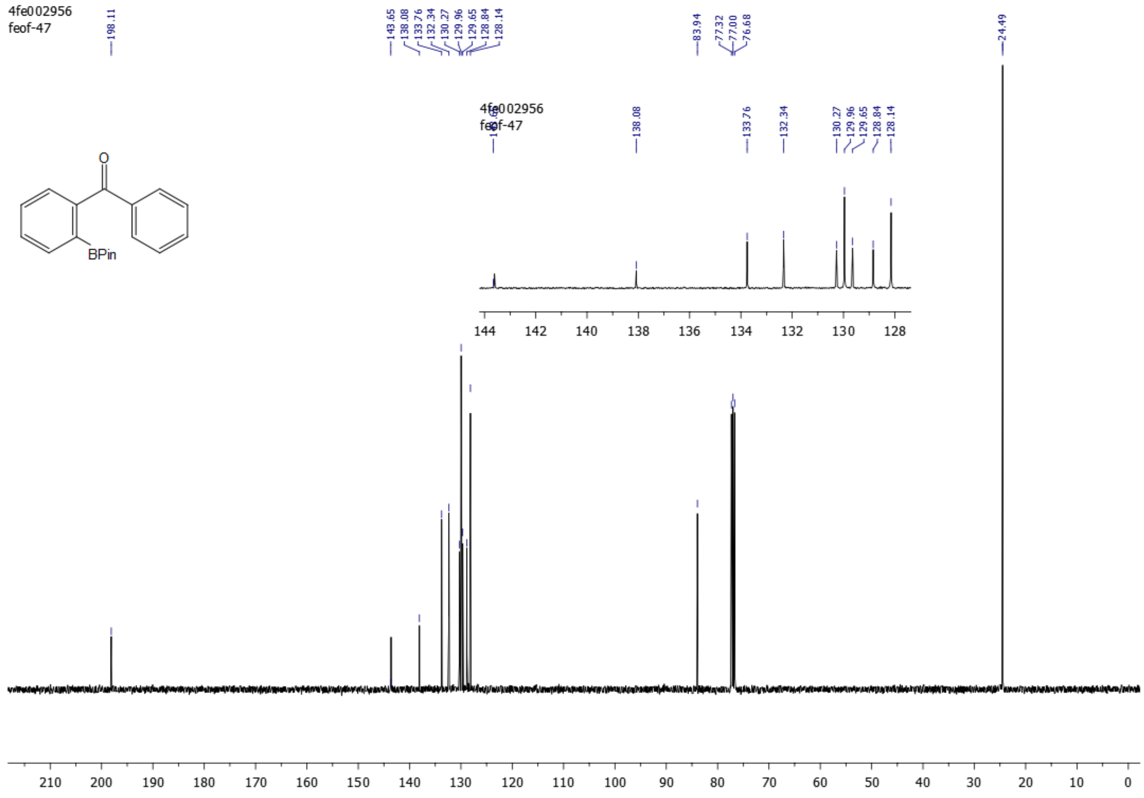

Supplementary Figure 46. ^{13}C NMR (101 MHz, $CDCl_3$, 293 K) of phenyl(2-(4,4,5,5-tetramethyl-1,3,2-dioxaborolan-2-yl)phenyl)methanone (**S4**).

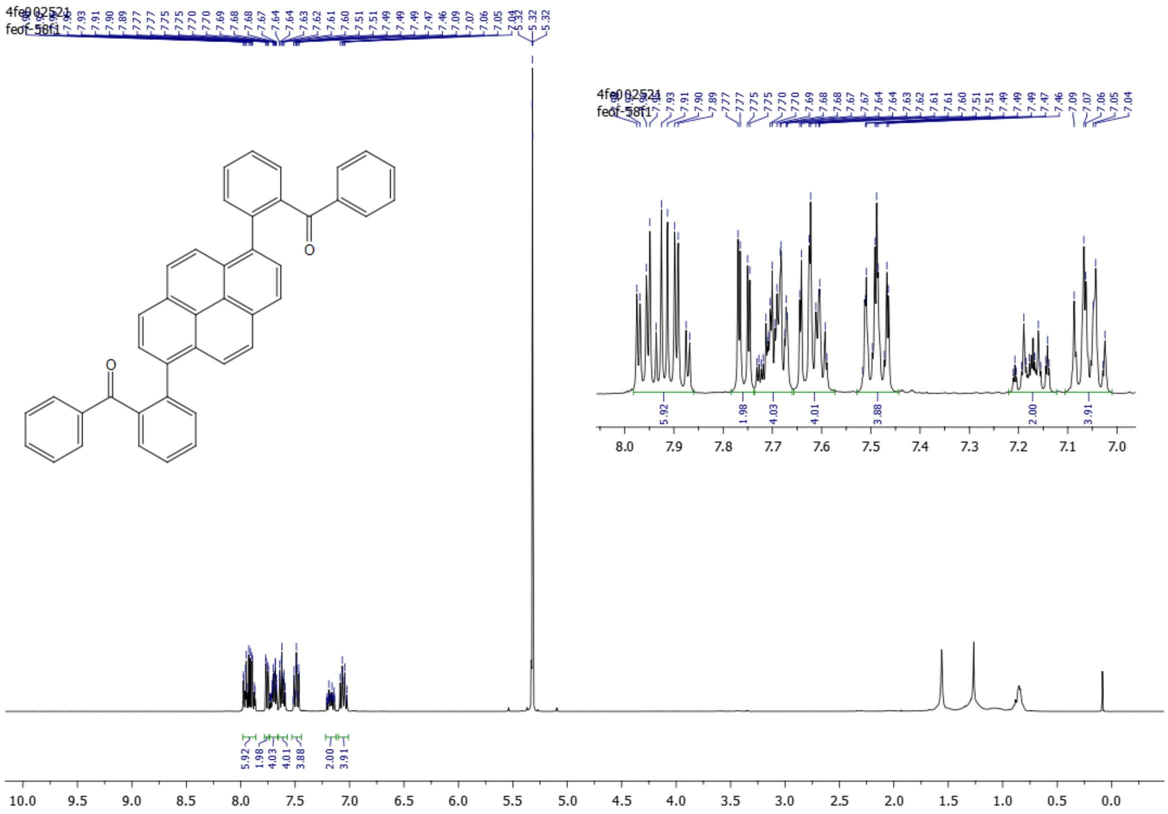

Supplementary Figure 47. ^{1}H NMR (400 MHz, CD_2Cl_2, 293 K) of (pyrene-1,6-diylbis(2,1-phenylene))bis(phenylmethanone) (**S6**).

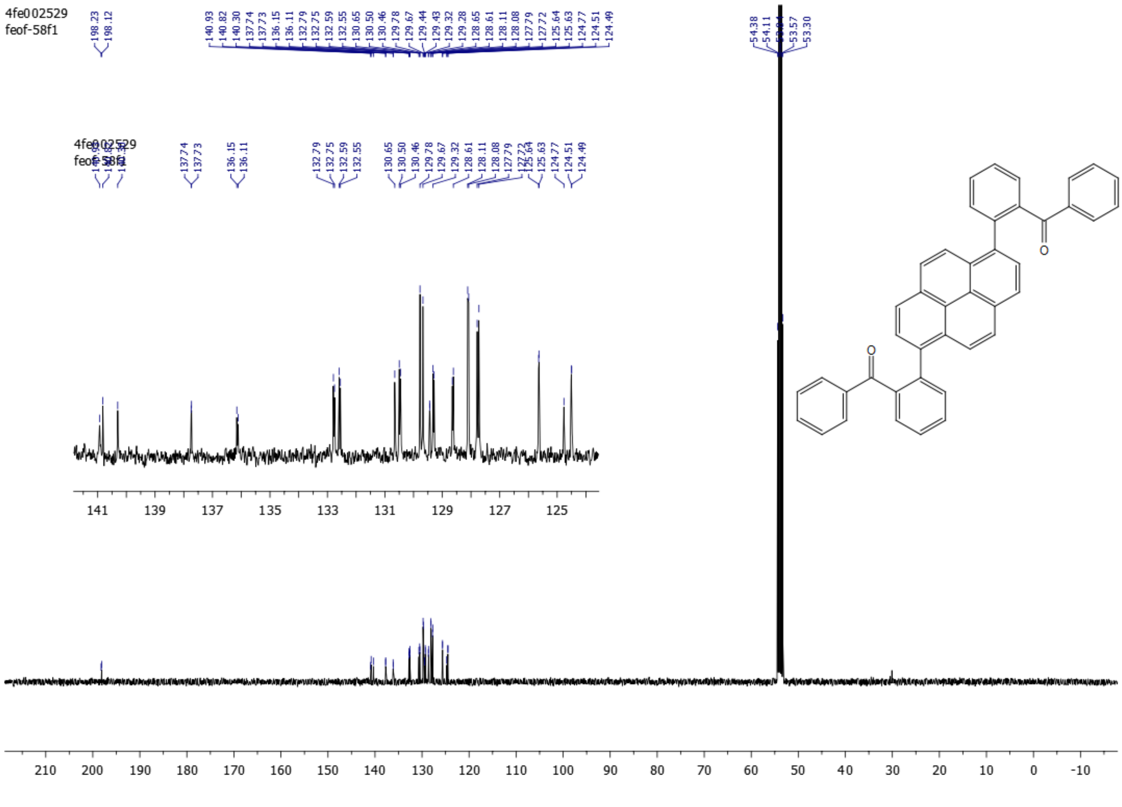

Supplementary Figure 48. ^{13}C NMR (101 MHz, CD_2Cl_2, 293 K) of (pyrene-1,6-diylbis(2,1-phenylene))bis(phenylmethanone) (**S6**).

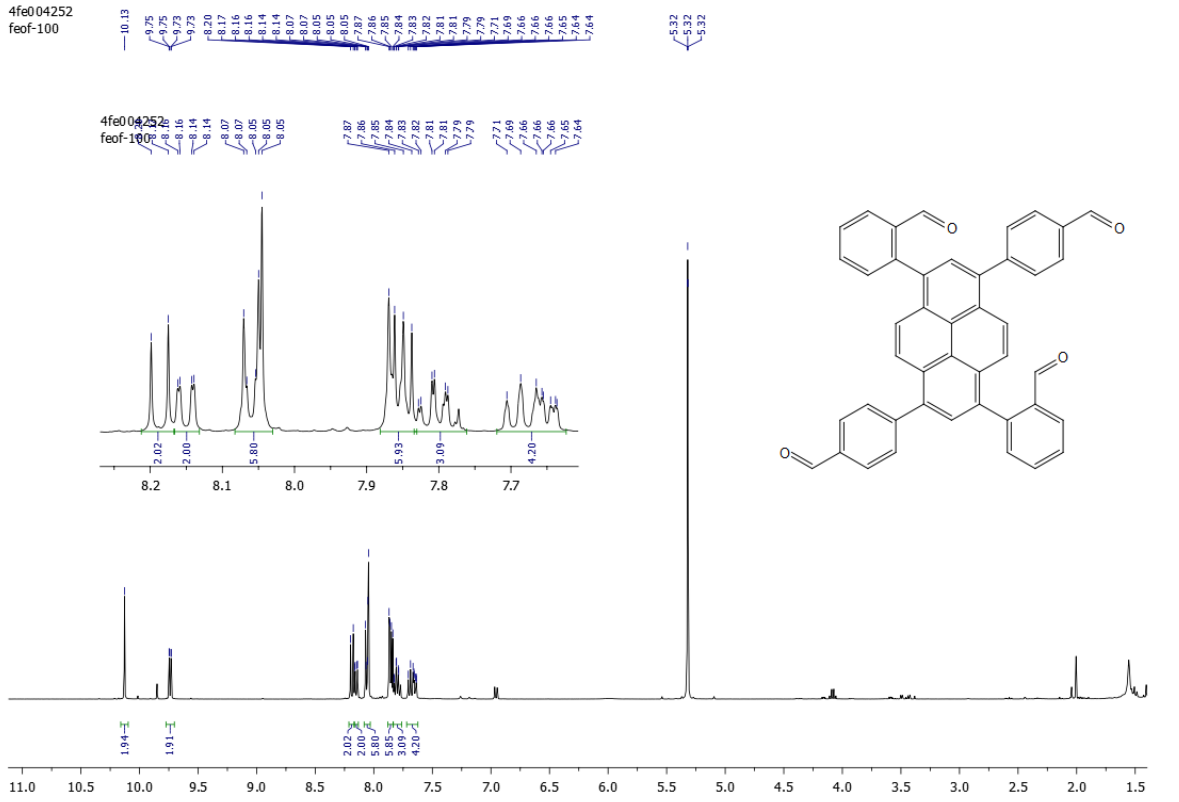

Figure S49. ^{1}H NMR (400 MHz, CD_2Cl_2, 293 K) of 4,4'-(3,8-bis(2-formylphenyl)pyrene-1,6-diyl)dibenzaldehyde (**16**).

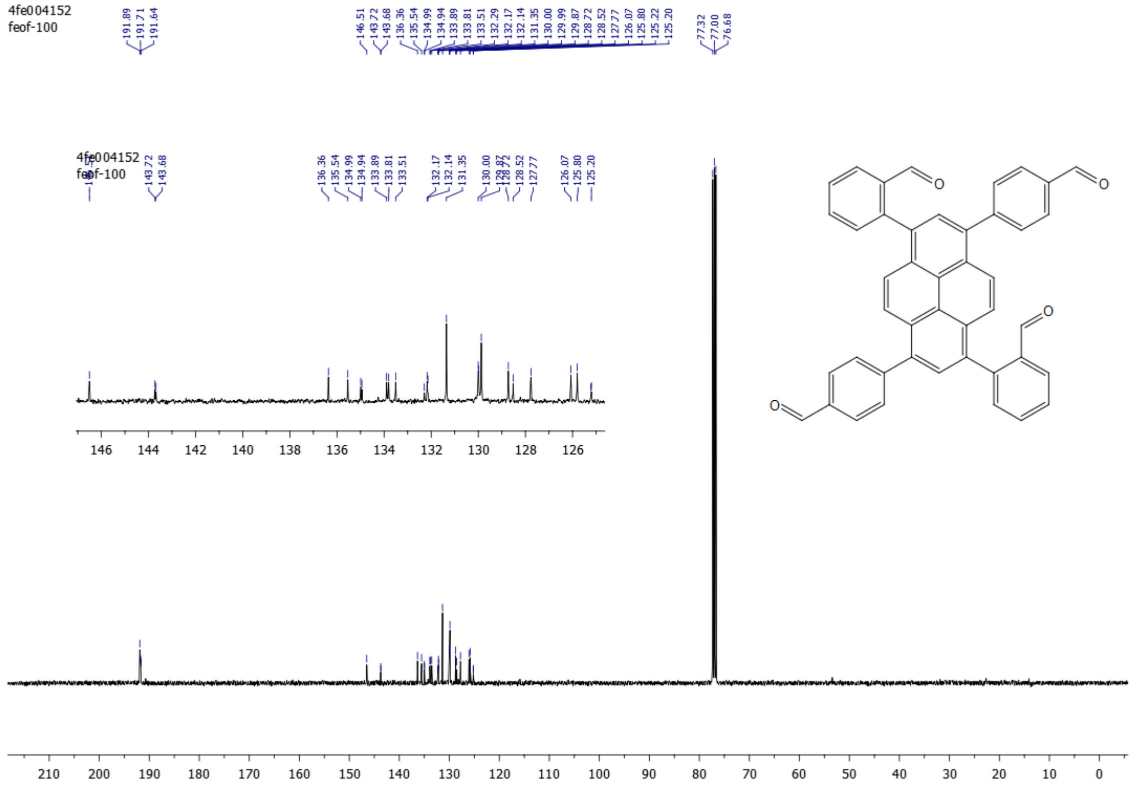

Supplementary Figure 50. ^{13}C NMR (101 MHz, CD_2Cl_2, 293 K) of 4,4'-(3,8-bis(2-formylphenyl)pyrene-1,6-diyl)dibenzaldehyde (**16**).

Spectral appendix (MS, HRMS, HPLC, UV-Vis)

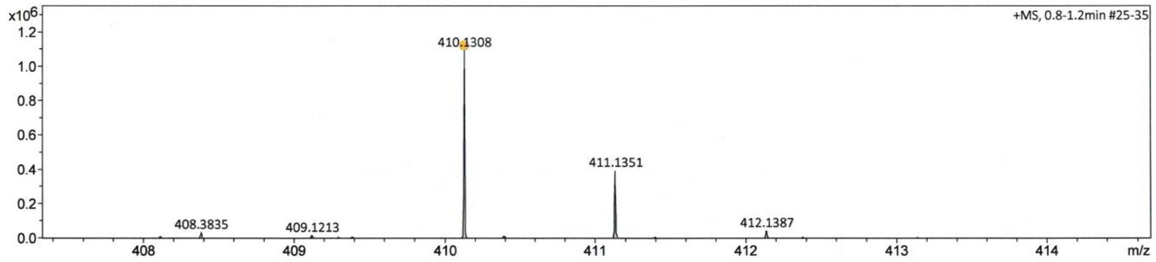

Supplementary Figure 51. APPI-HRMS spectrum of 1,6-bis(2-formylphenyl)pyrene (**3**).

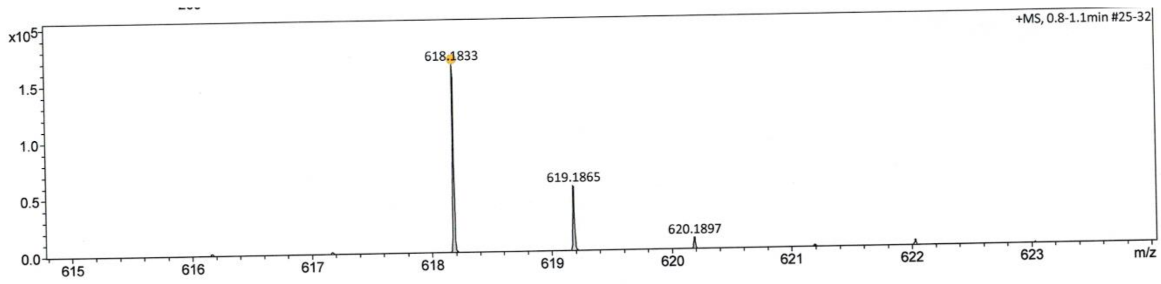

Supplementary Figure 52. APPI-HRMS spectrum 1,3,6,8-tetrakis(2-formylphenyl)pyrene (**8**).

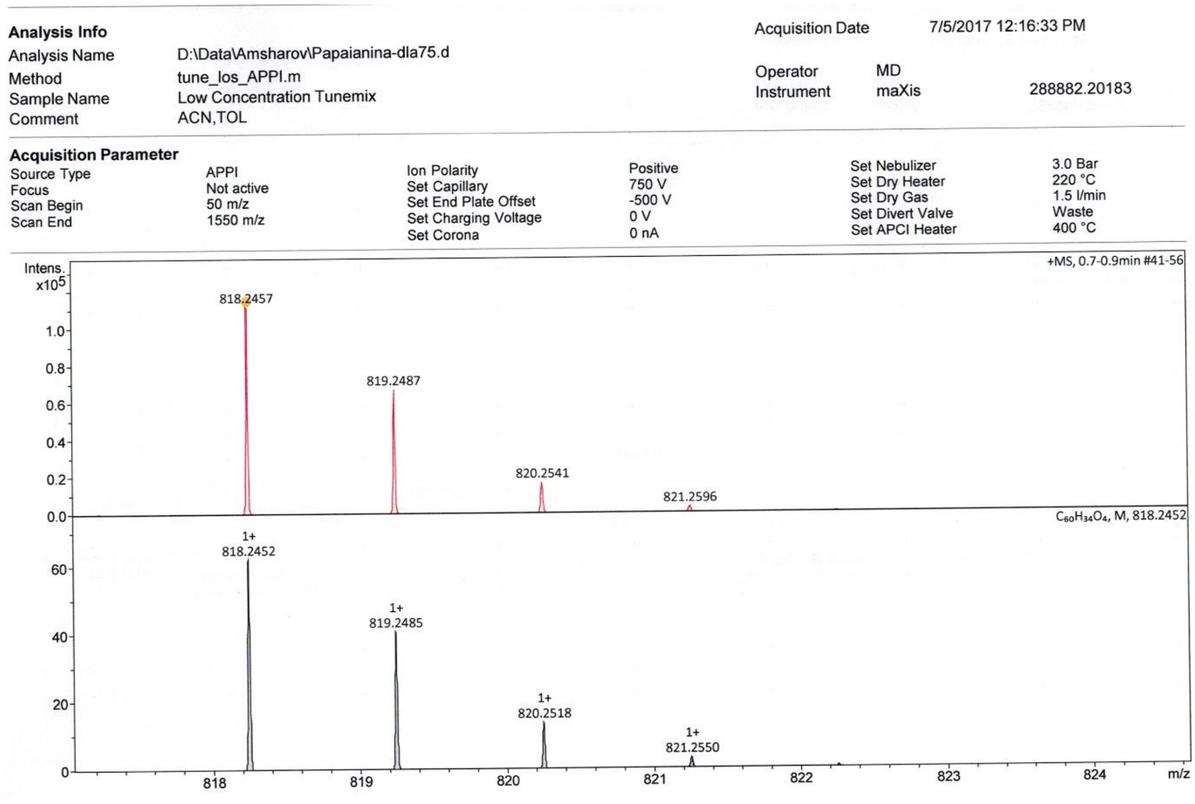

Supplementary Figure 53. APPI-MS spectrum of 1,3,6,8-tetrakis(3-formylnaphthyl)pyrene (**9**).

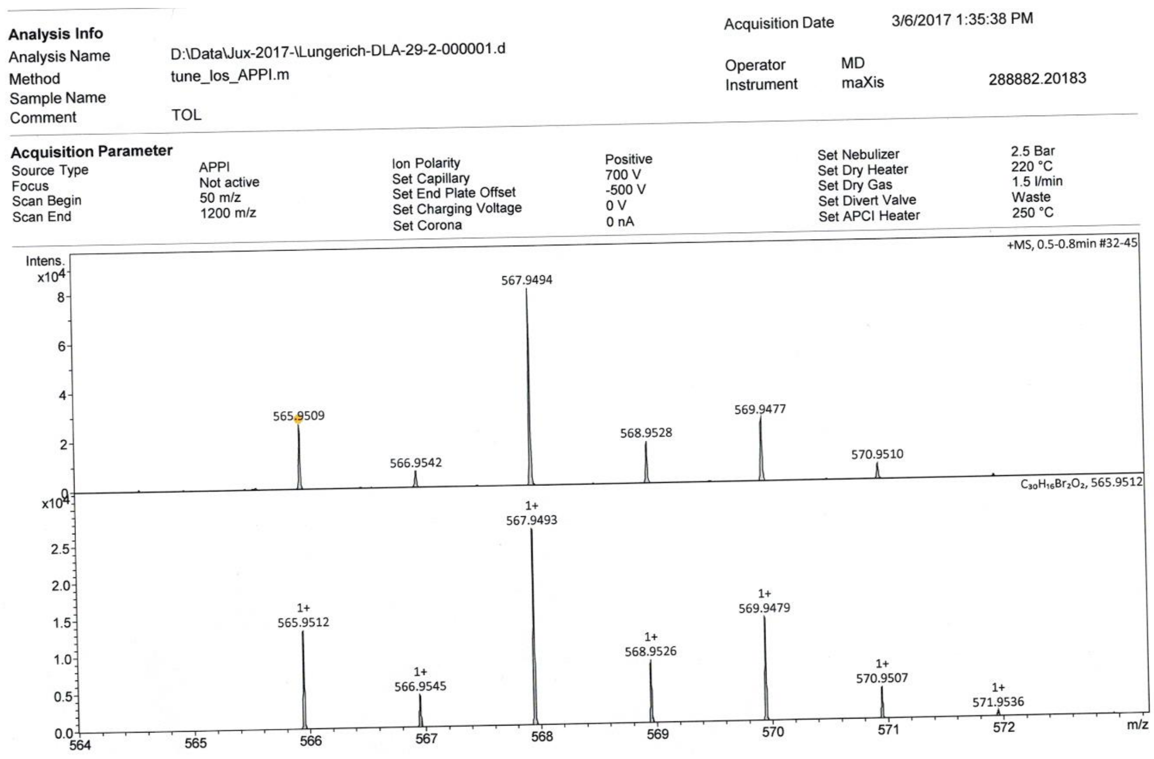

Supplementary Figure 54. APPI-HRMS spectrum of 1,6-dibromo-3,8-bis(2-formylphenyl)pyrene (**6**).

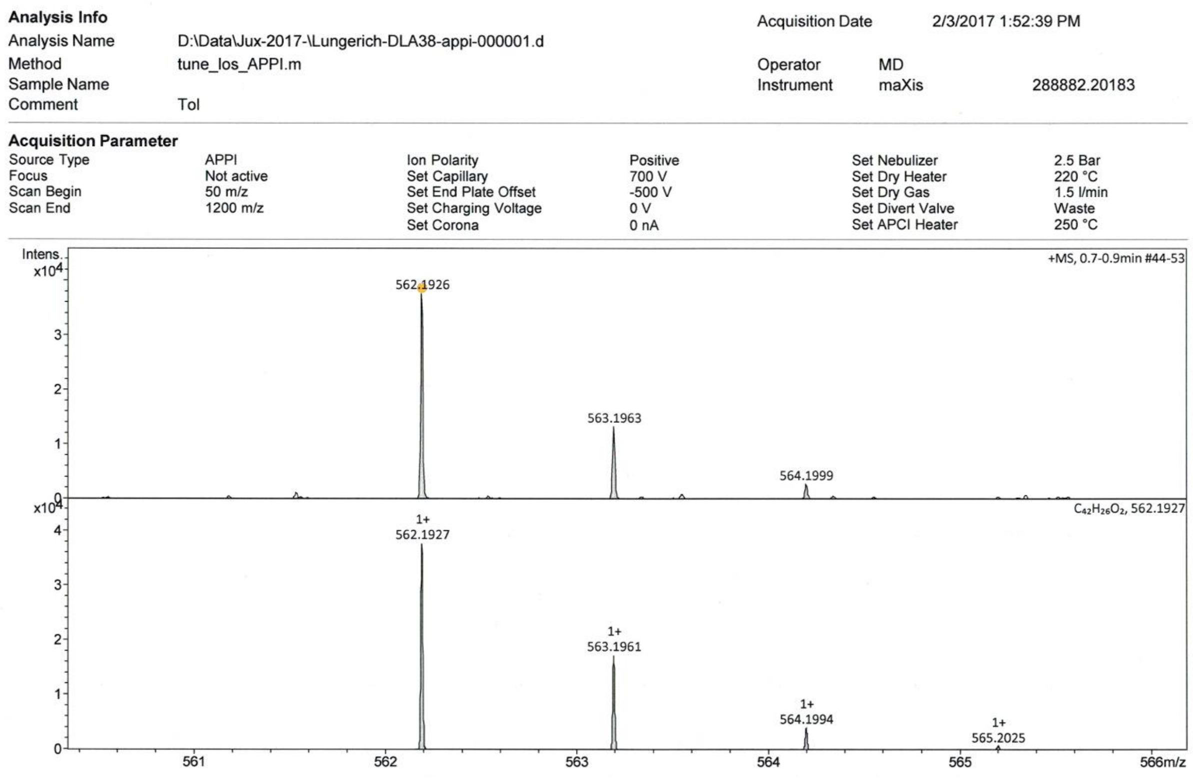

Supplementary Figure 55. APPI-HRMS spectrum of 1,6-bis(2-formylphenyl)-3,8-diphenylpyrene (**3a**).

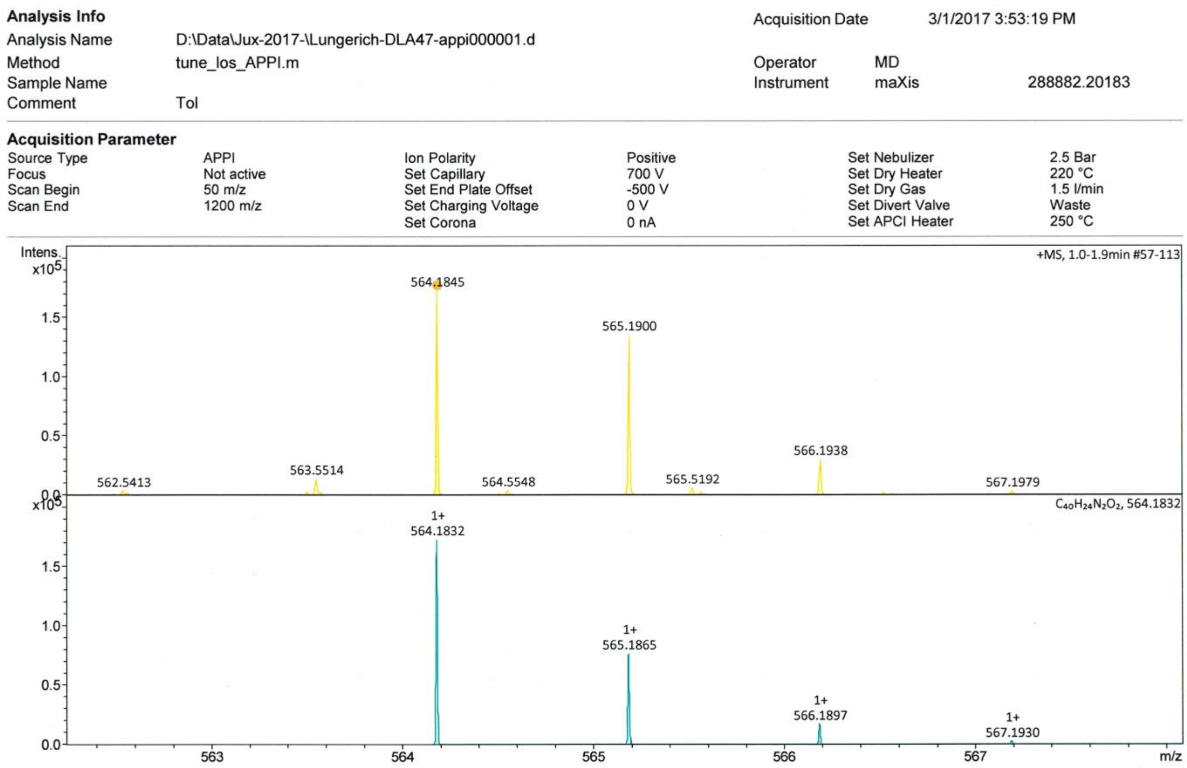

Supplementary Figure 56. APPI-HRMS spectrum of 1,6-bis(2-formylphenyl)-3,8-bis(4-pyridyl)pyrene (**S5**).

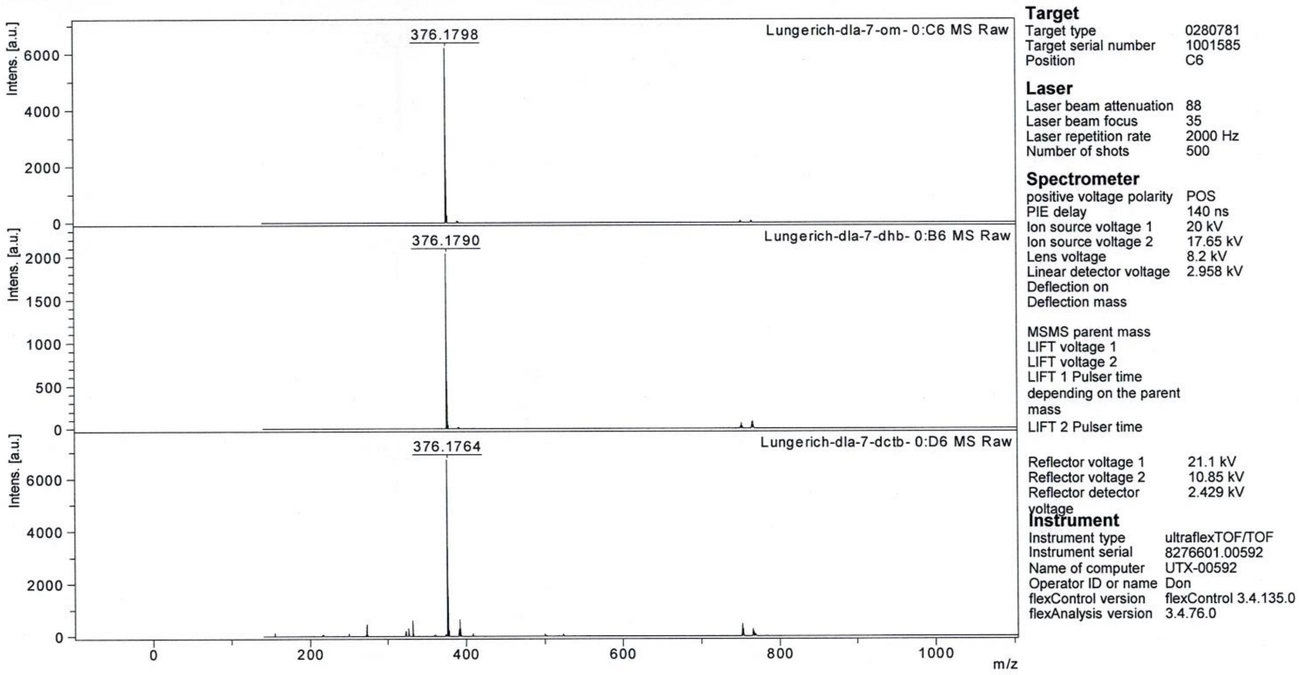

Supplementary Figure 57. MALDI-TOF MS spectrum of **DBATT**; top: without matrix (LDI); middle: DHB matrix; bottom: DCTB matrix.

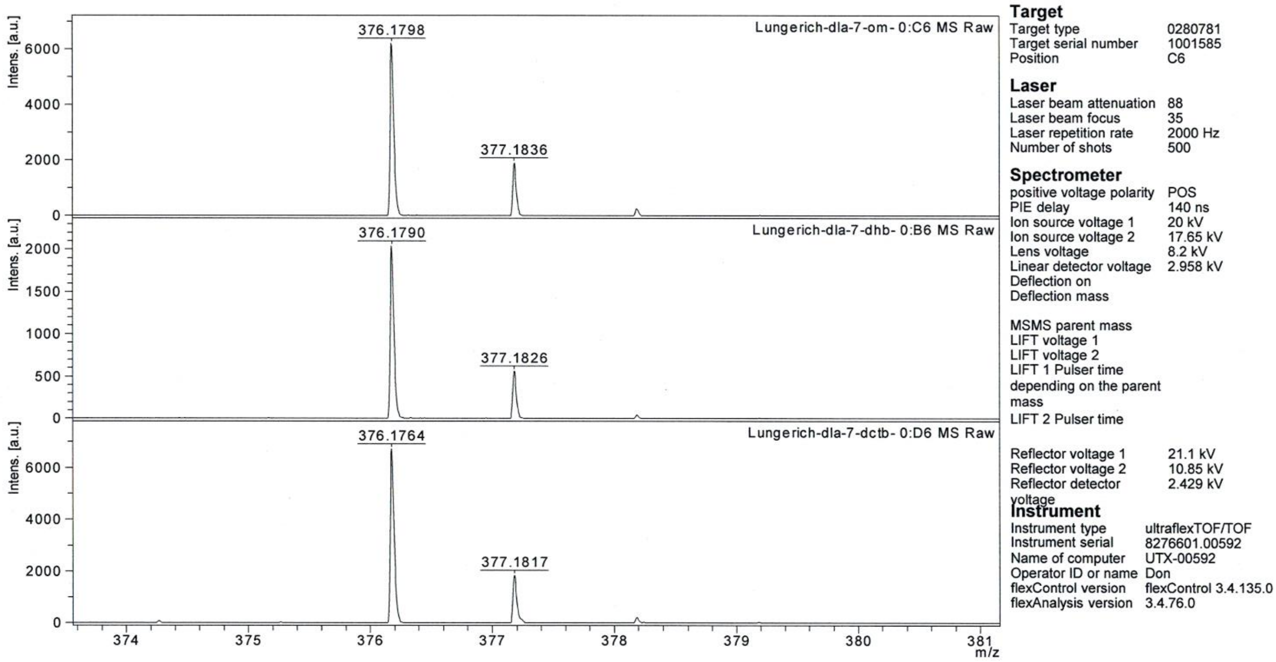

Supplementary Figure 58. MALDI-TOF HRMS spectrum of **DBATT**; top: without matrix (LDI); middle: DHB matrix; bottom: DCTB matrix.

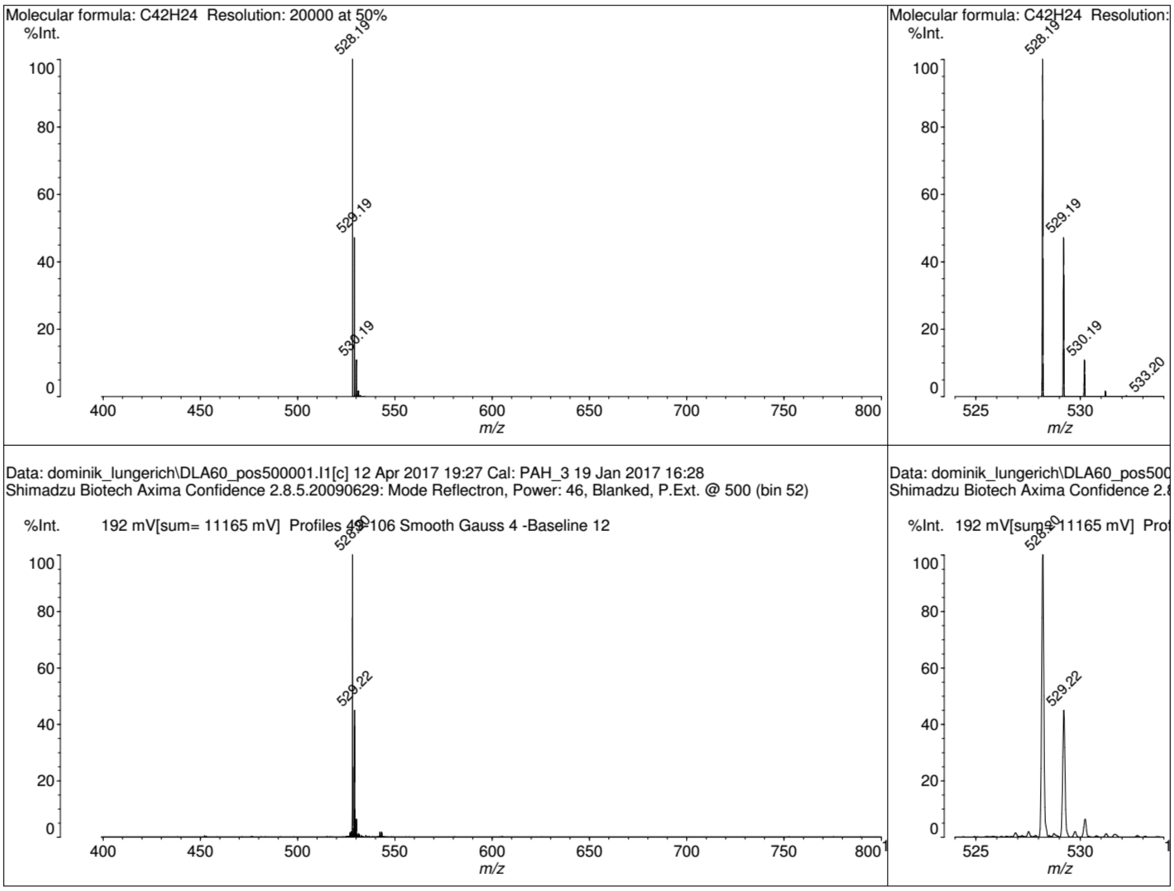

Supplementary Figure 59. LDI-TOF MS spectrum of 6,14-bisphenyl-2.3,8.9-dibenzanthanthrene (**bPh-DBATT**).

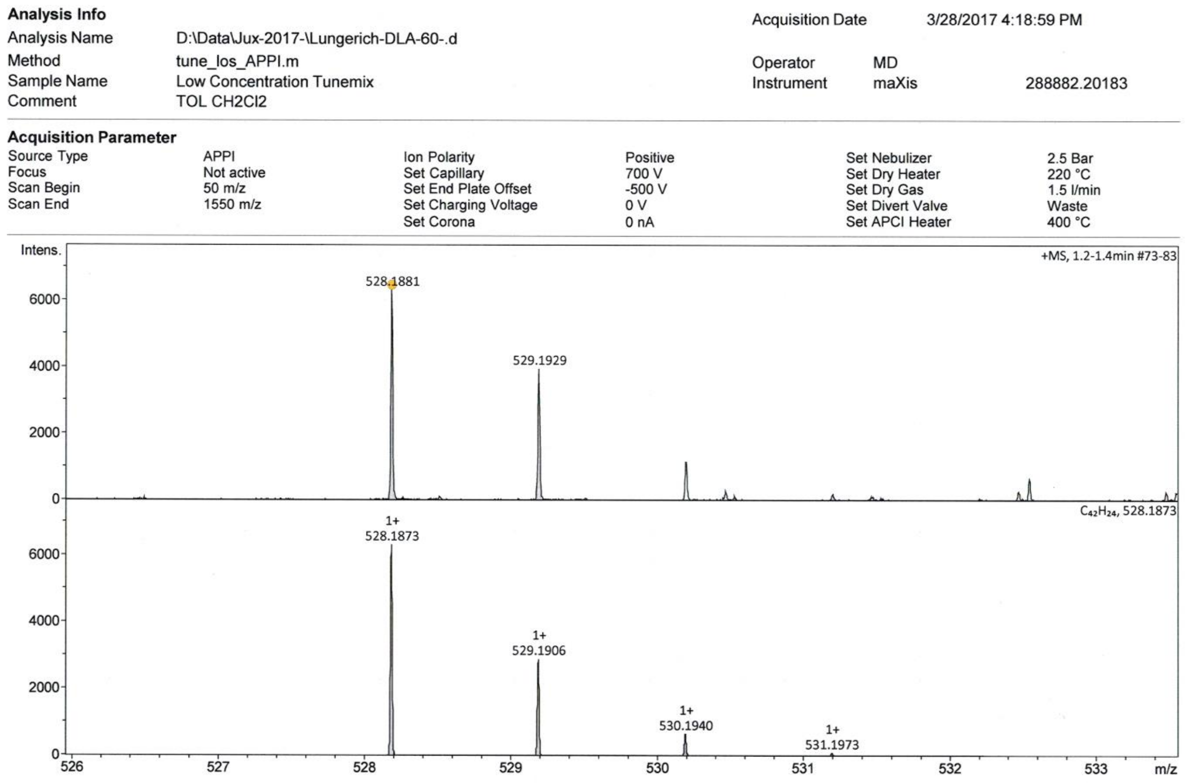

Supplementary Figure 60. APPI-HRMS spectrum of 6,14-bisphenyl-2.3,8.9-dibenzanthanthrene (**bPh-DBATT**).

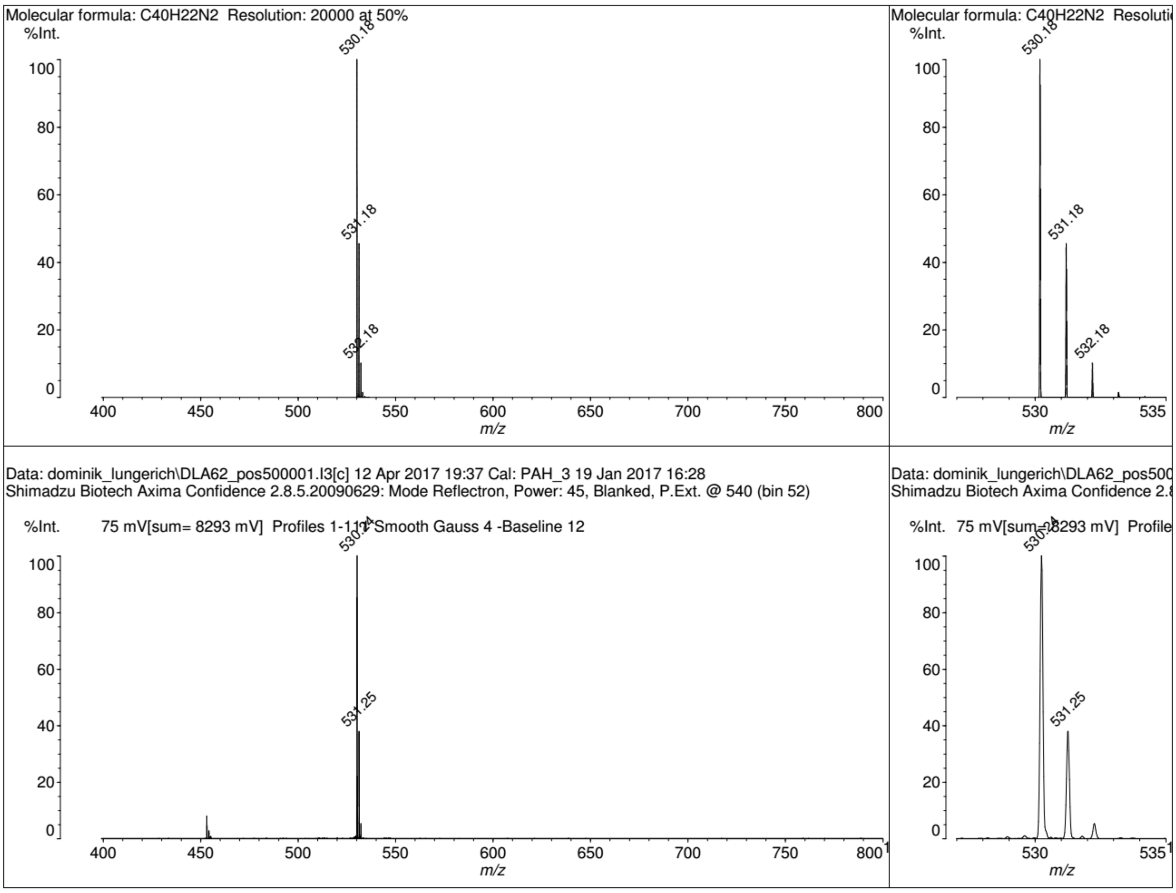

Supplementary Figure 61. LDI-TOF MS spectrum of 6,14-bis(4-pyridinyl)-2.3,8.9-dibenzanthanthrene (**S7**).

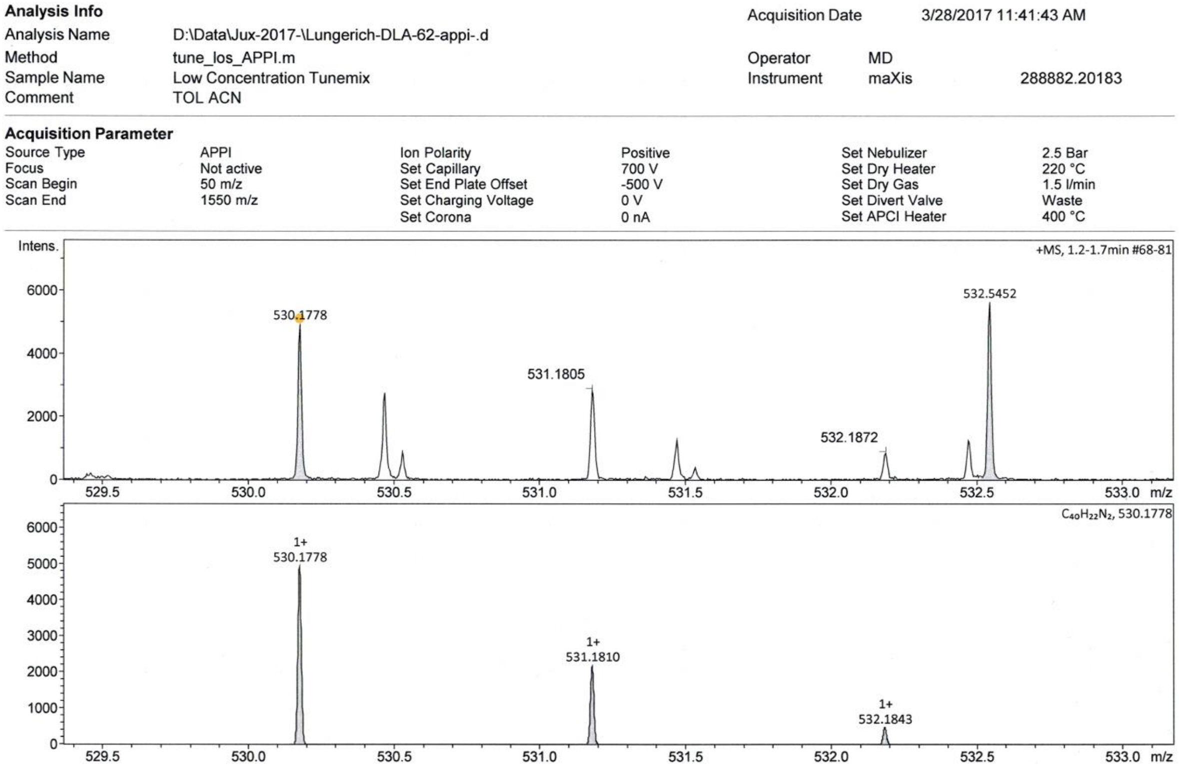

Supplementary Figure 62. APPI-HRMS spectrum of 6,14-bis(4-pyridinyl)-2.3,8.9-dibenzanthanthrene (**S7**).

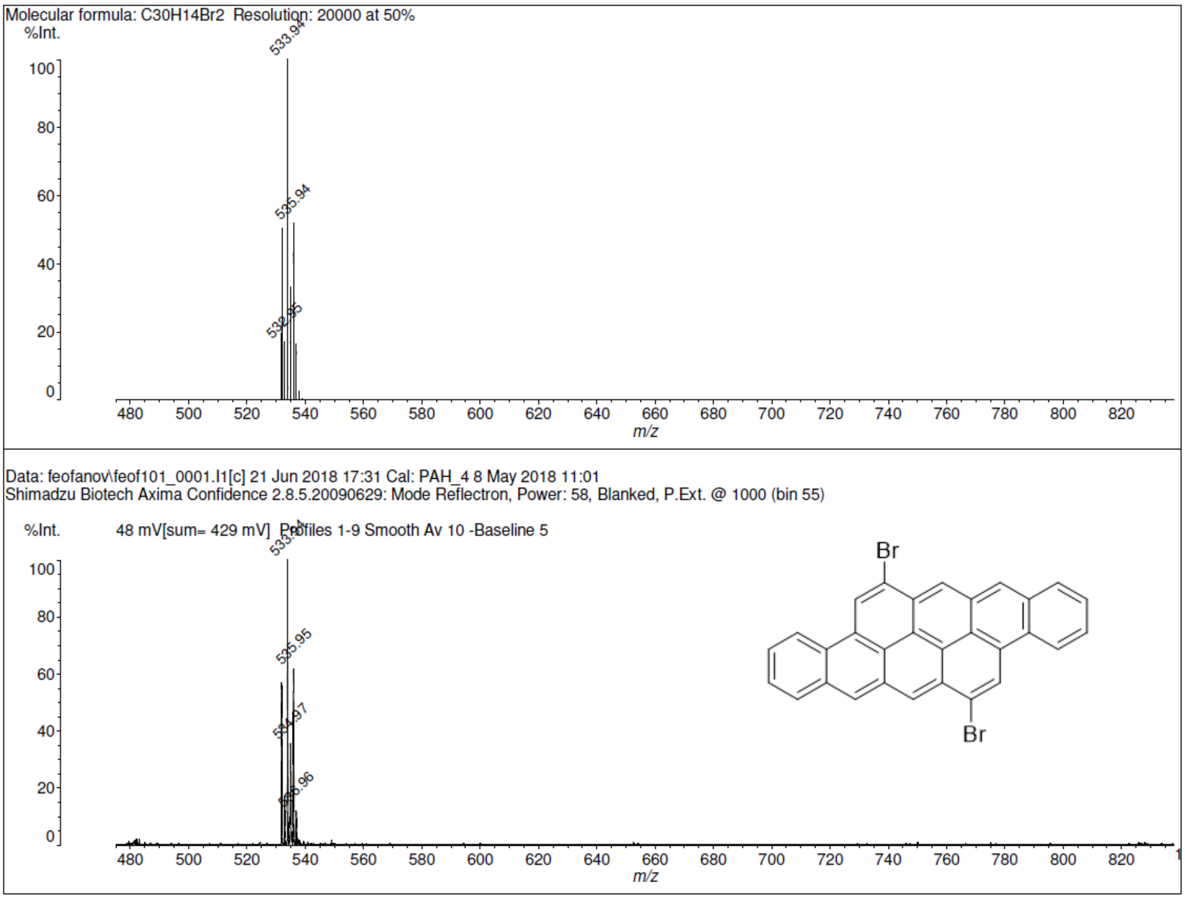

Supplementary Figure 63. LDI-TOF MS spectrum of 6,14-dibromo-2.3,8.9-dibenzanthanthrene (**bBr-DBATT**).

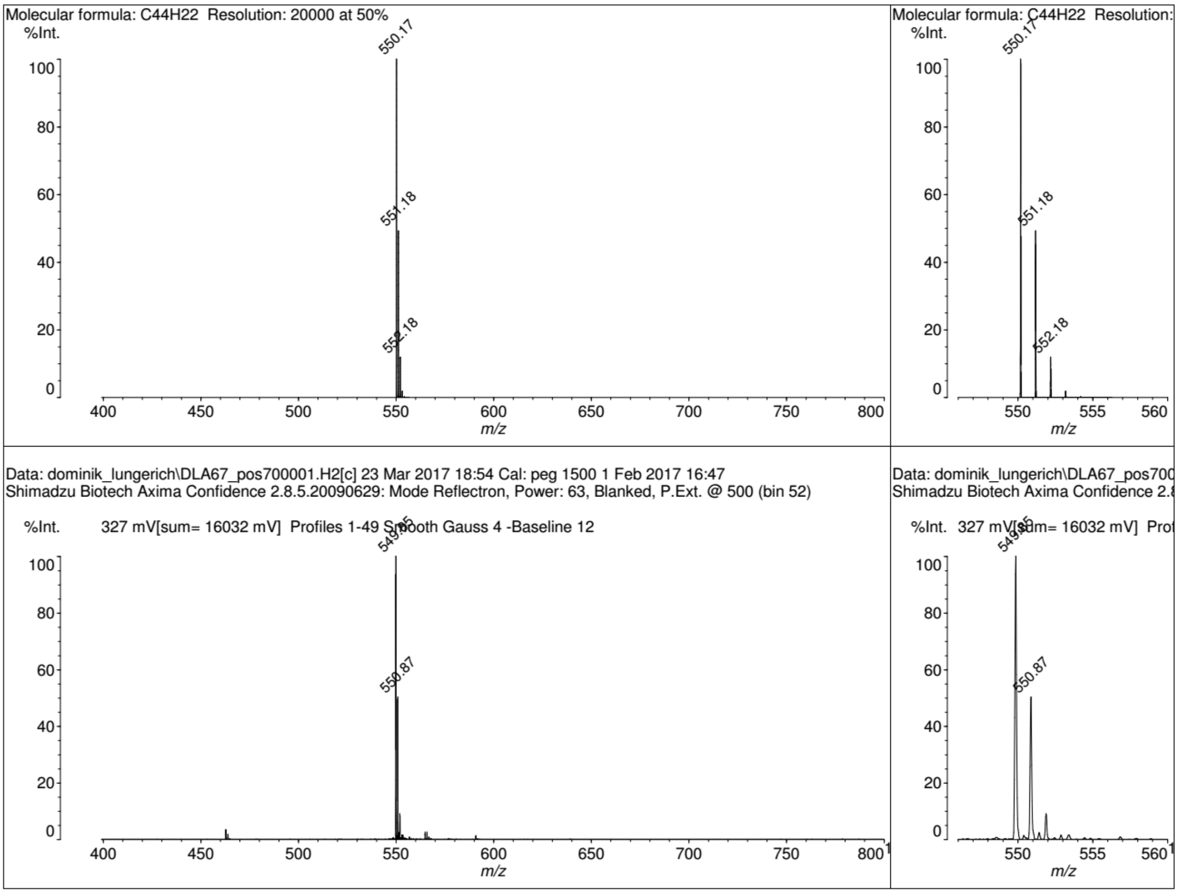

Supplementary Figure 64. LDI-TOF MS spectrum of tetrakis-tetracene (**TTc**).

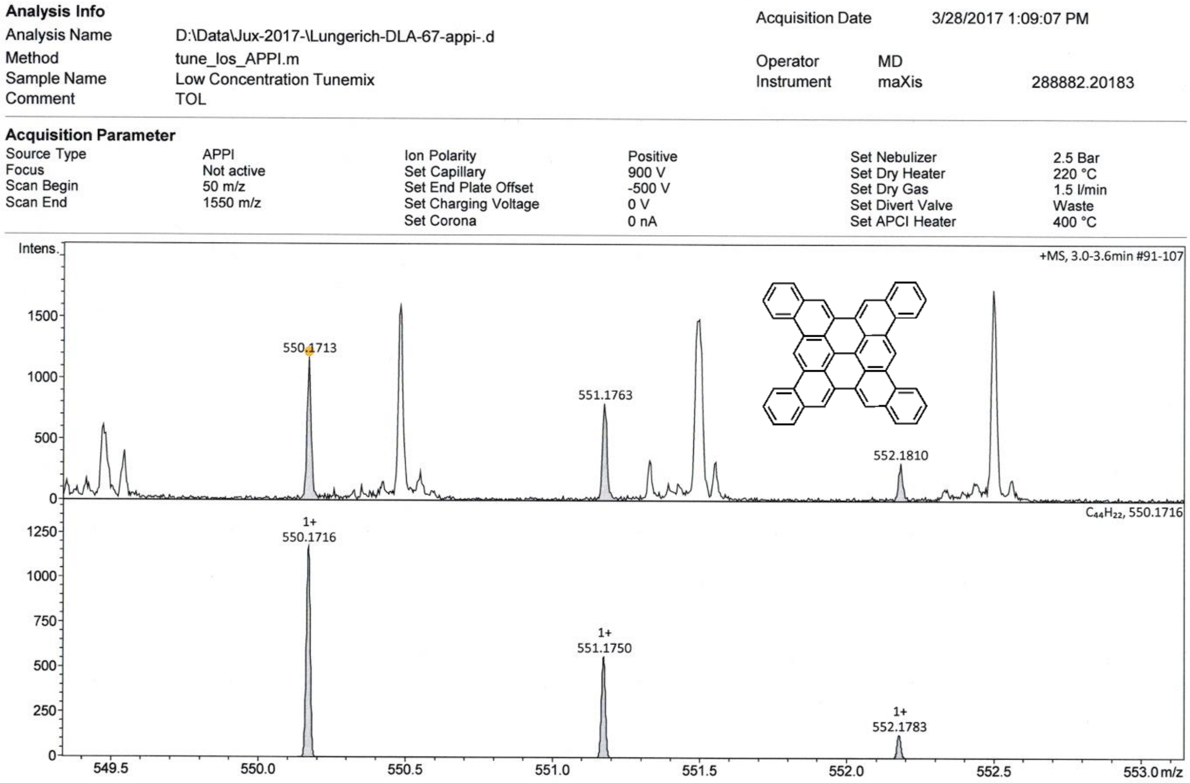

Supplementary Figure 65. APPI-HRMS spectrum of tetrakis-tetracene (**TTc**).

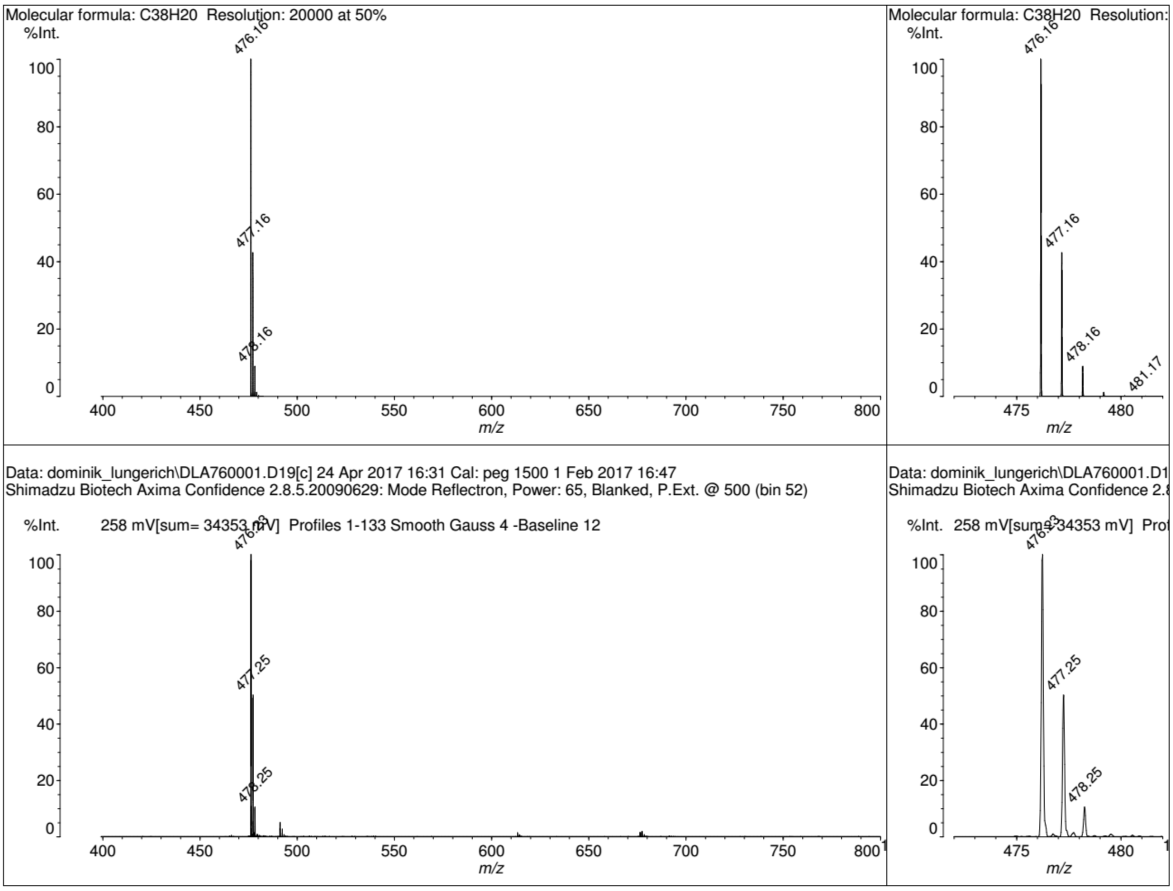

Supplementary Figure 66. LDI-TOF MS spectrum of bis-pentacene (**BPc**).

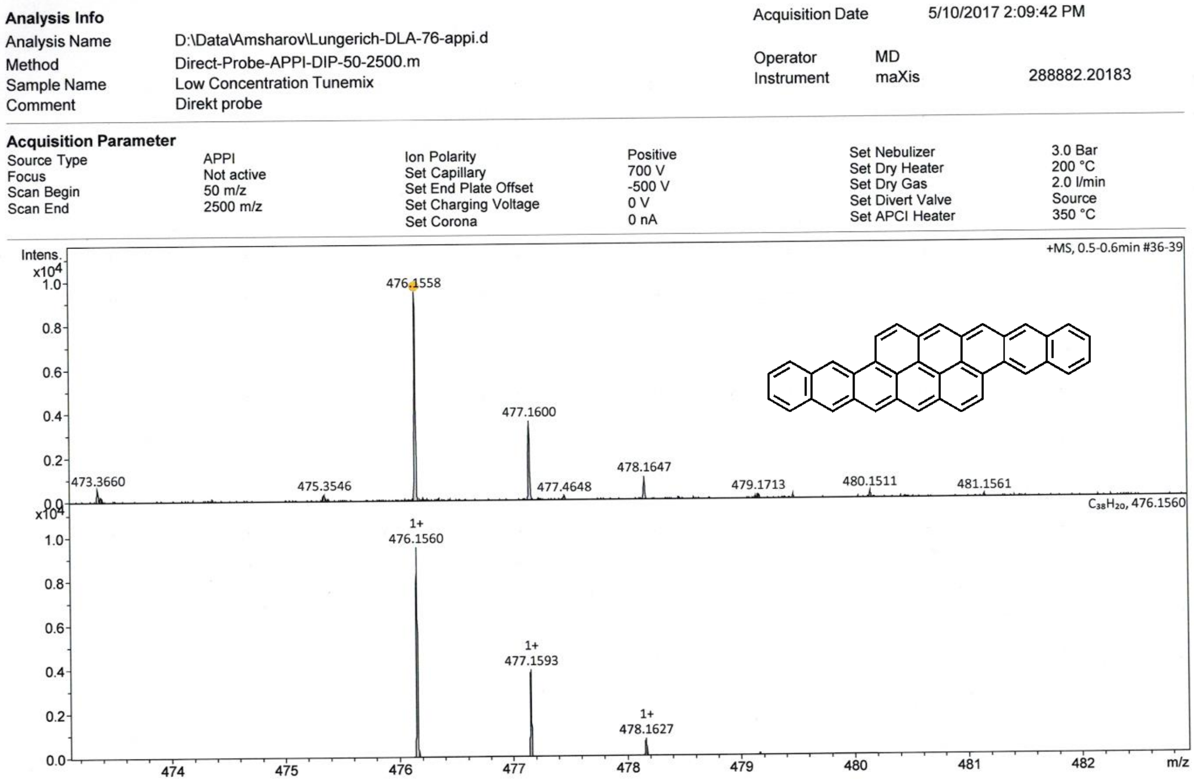

Supplementary Figure 67. APPI-HRMS spectrum of bis-pentacene (**BPc**).

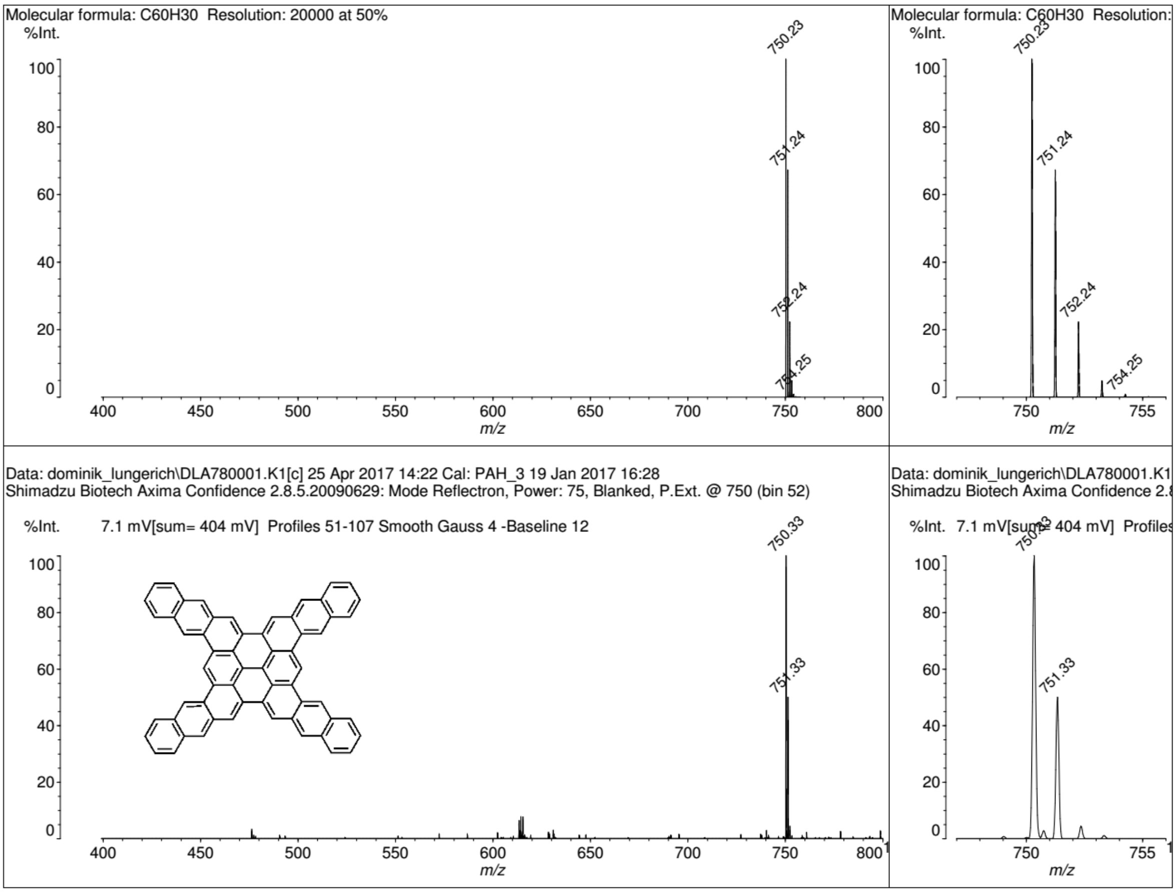

Supplementary Figure 68. LDI-TOF MS spectrum of tetrakis-pentacene (**TPc**).

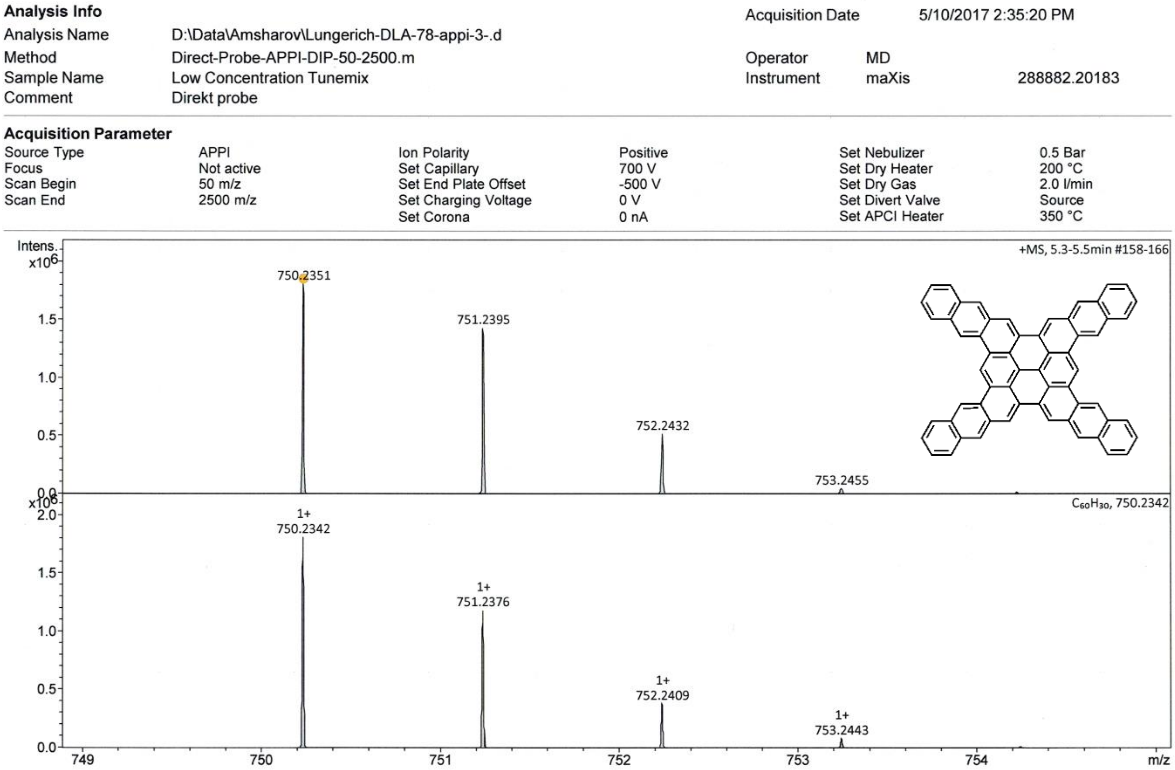

Supplementary Figure 69. APPI-HRMS spectrum of tetrakis-pentacene (**TPc**).

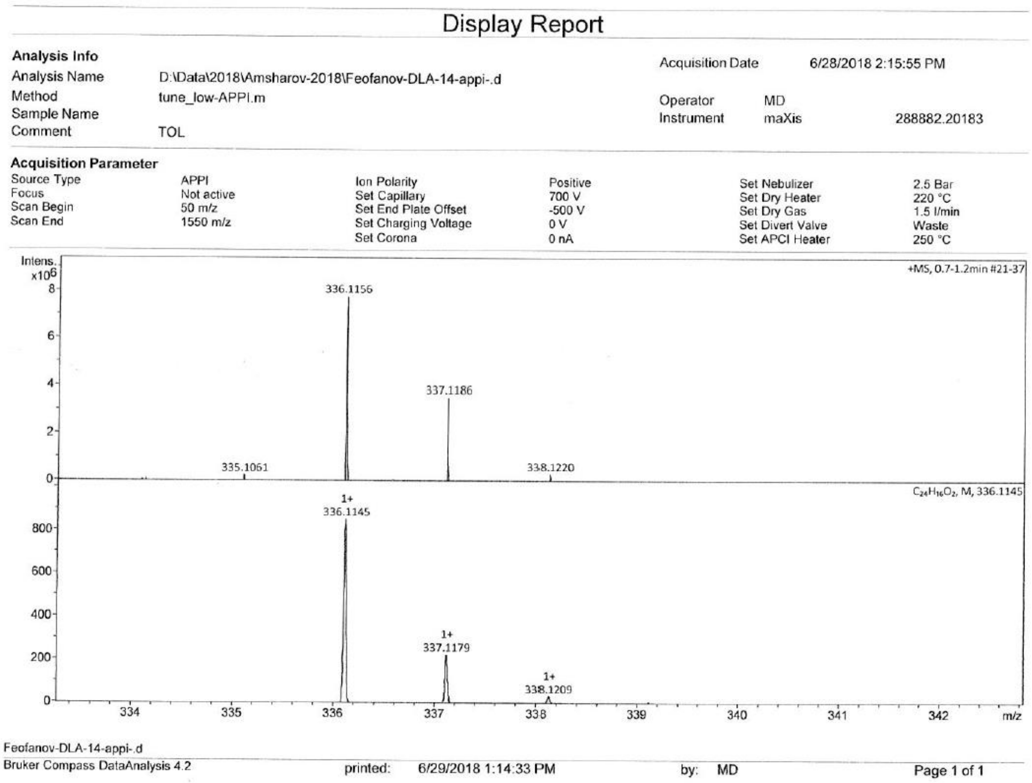

Supplementary Figure 70. APPI-HRMS spectrum of 2,2'-(naphthalene-1,4-diyl)dibenzaldehyde (**10**).

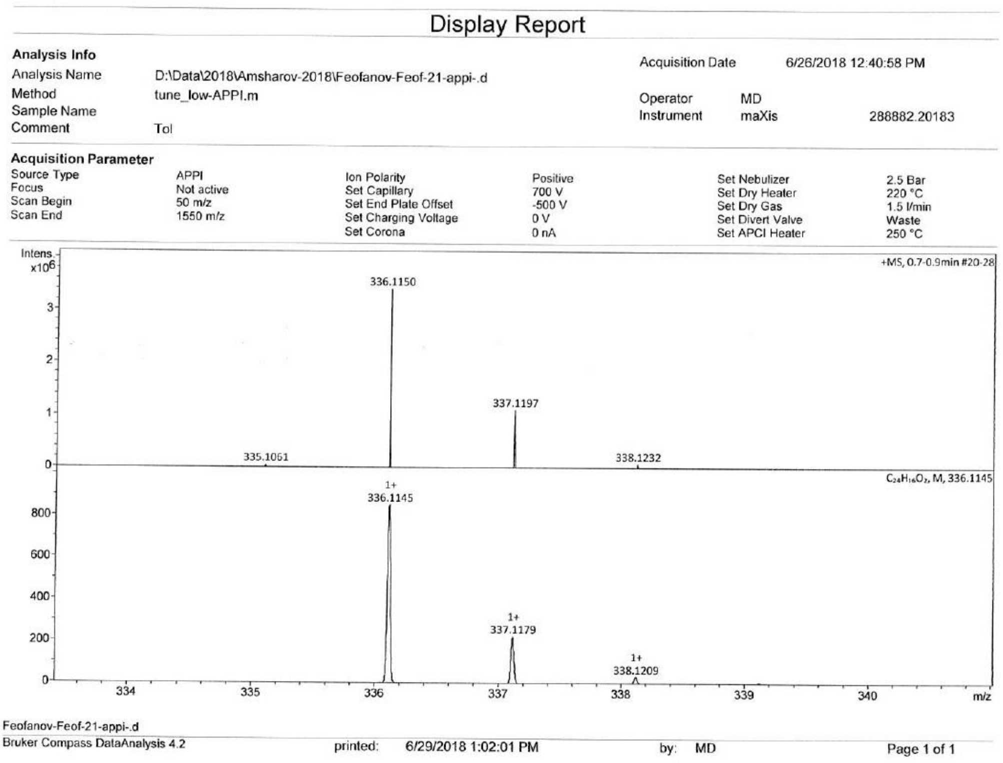

Supplementary Figure 71. APPI-HRMS spectrum of 2,2'-(naphthalene-1,5-diyl)dibenzaldehyde (**12**).

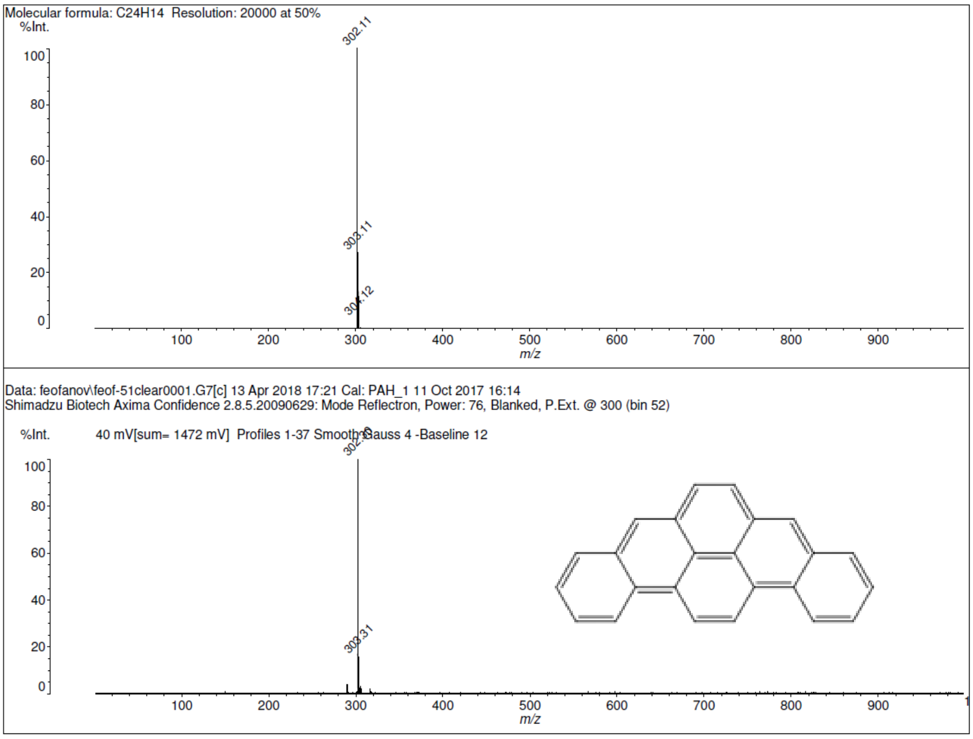

Supplementary Figure 72. LDI-TOF MS spectrum of benzo[*rst*]pentaphene (**11**).

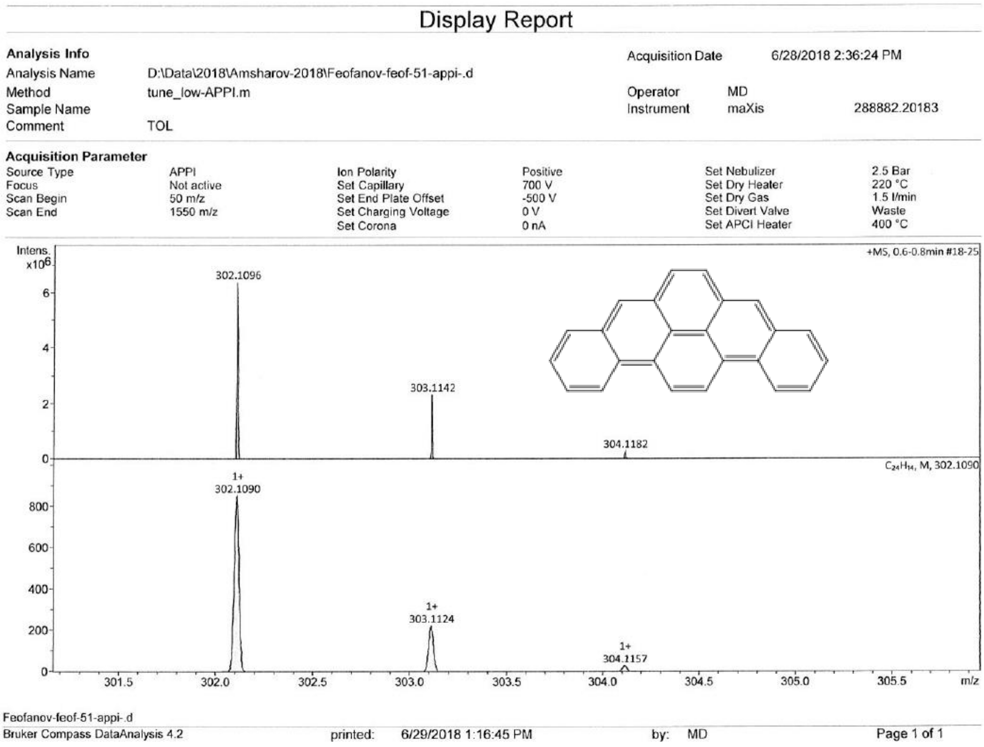

Supplementary Figure 73. APPI-HRMS spectrum of of benzo[*rst*]pentaphene (**11**).

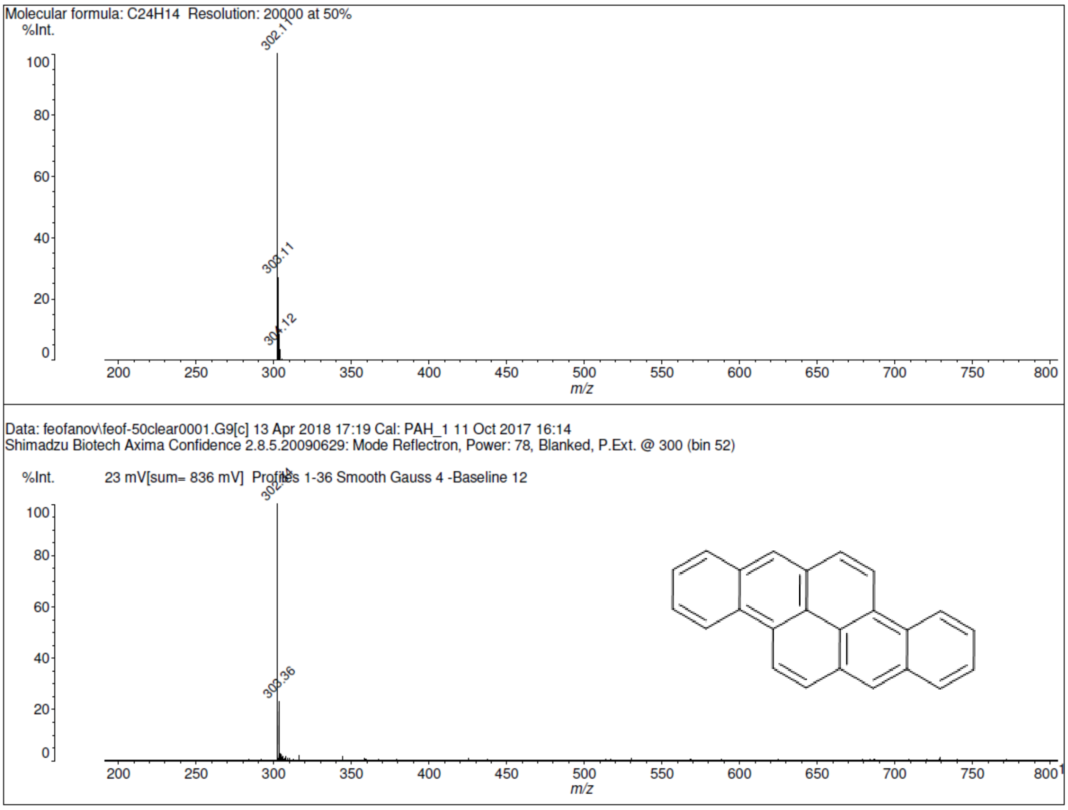

Supplementary Figure 74. LDI-TOF MS spectrum of dibenzo[b,def]chrysene (**13**).

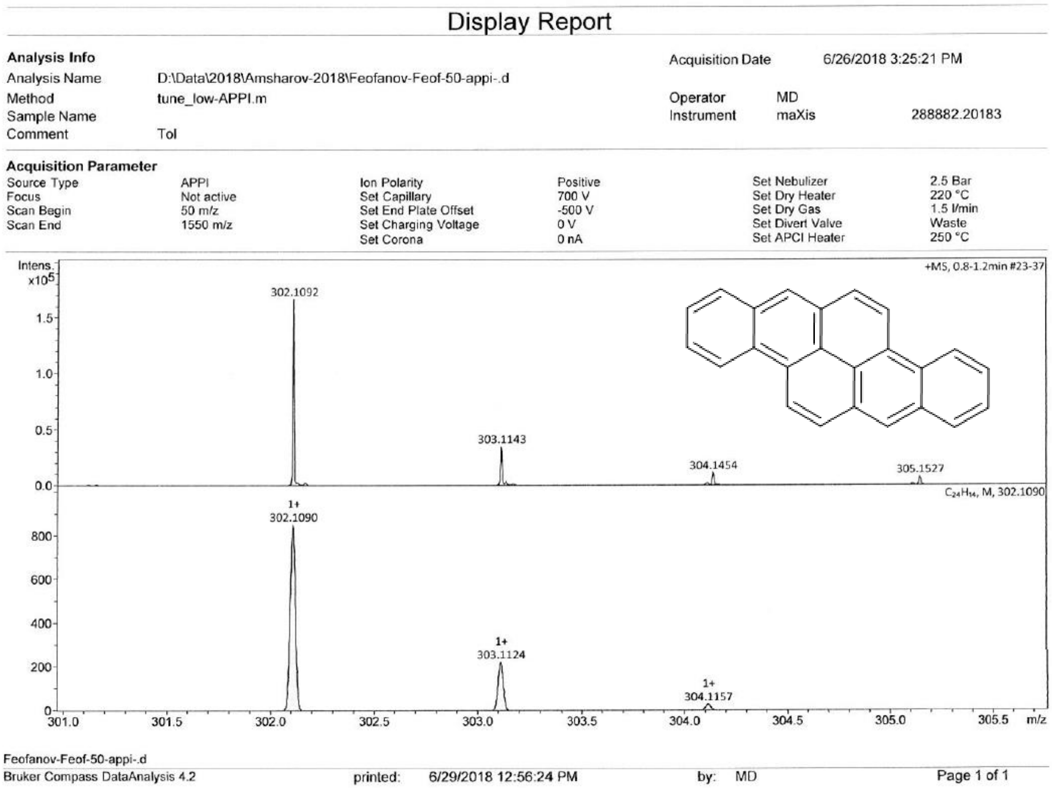

Supplementary Figure 75. APPI-HRMS spectrum of dibenzo[b,def]chrysene (**13**).

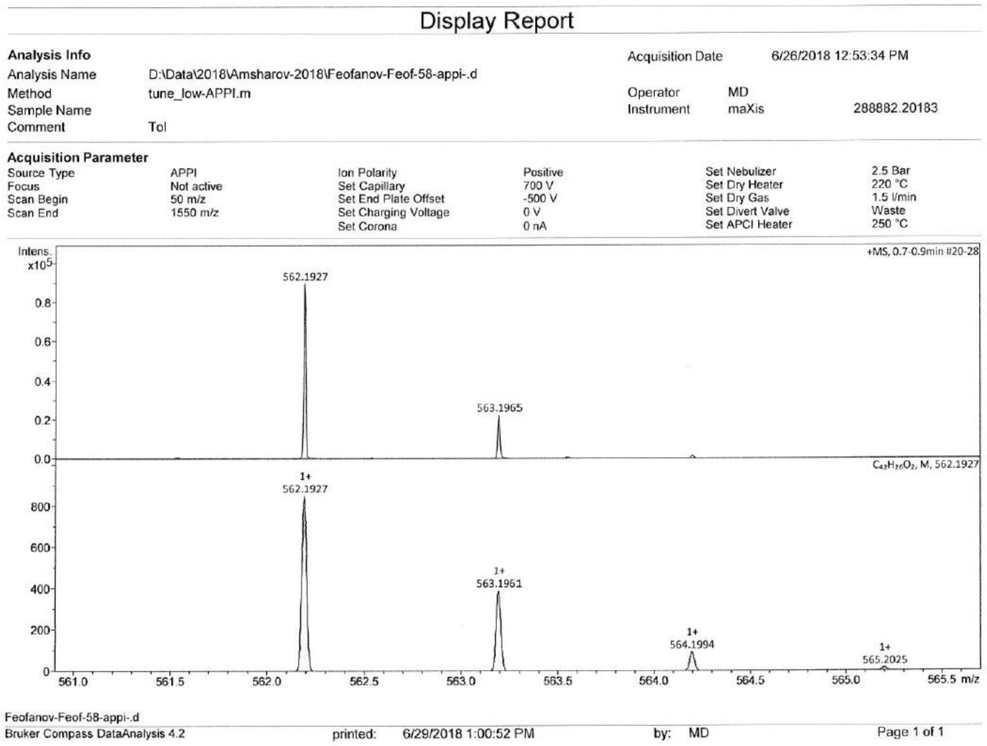

Supplementary Figure 76. APPI-HRMS spectrum of (pyrene-1,6-diylbis(2,1-phenylene))bis(phenylmethanone) (**S6**).

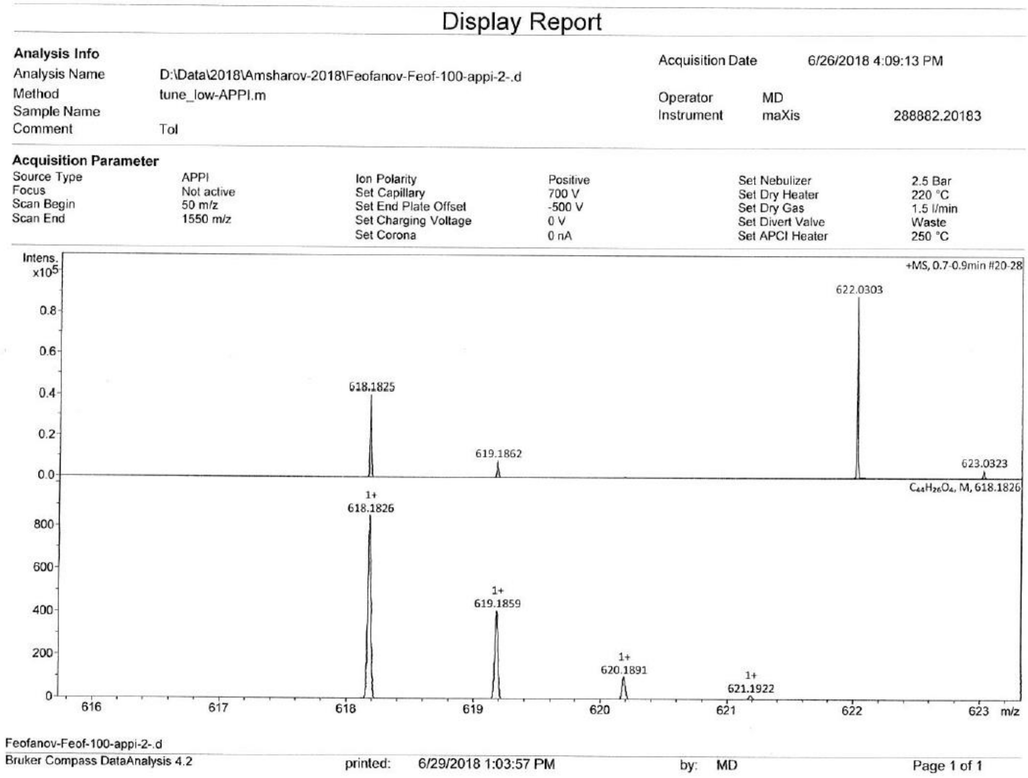

Supplementary Figure 77. APPI-HRMS spectrum of 4,4'-(3,8-bis(2-formylphenyl)pyrene-1,6-diyl)dibenzaldehyde (**16**).

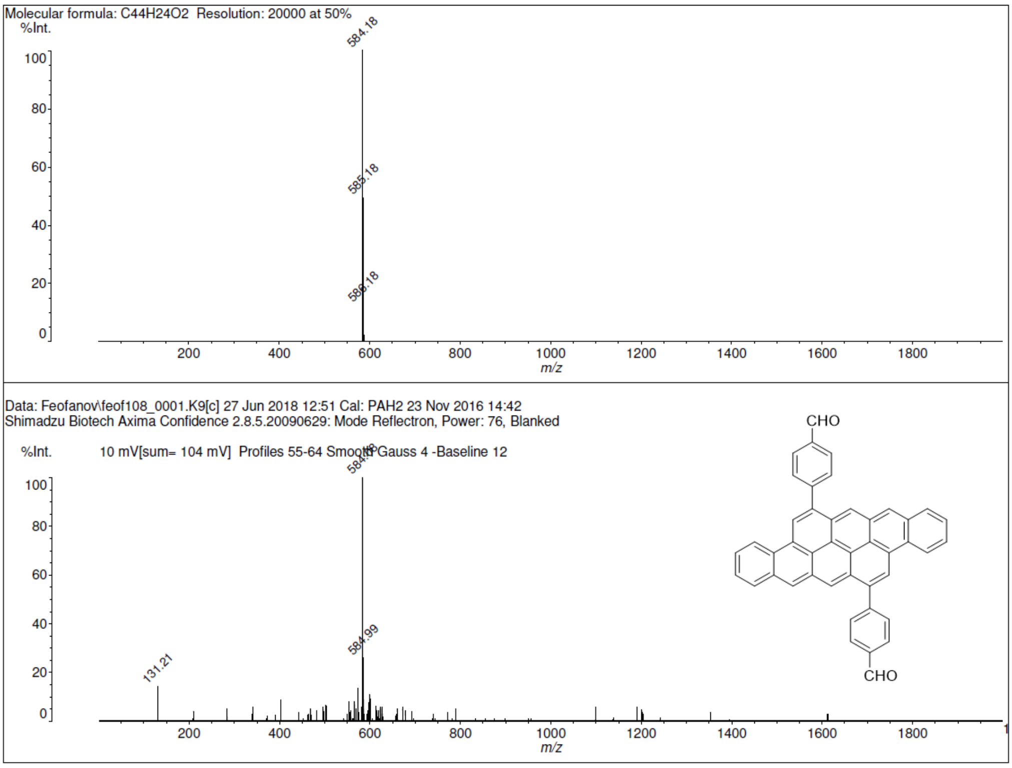

Supplementary Figure 78. LDI-TOF MS spectrum of 6,14-bis(*para*-formylphenyl)-2.3,8.9-dibenzanthanthrene (**17**).

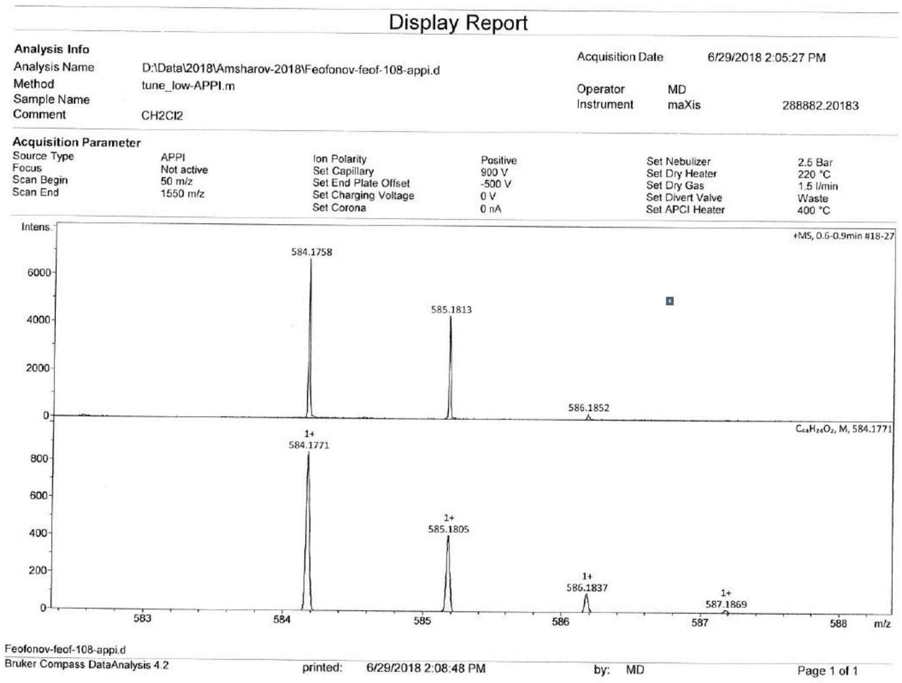

Supplementary Figure 79. APPI-HRMS spectrum of 6,14-bis(*para*-formylphenyl)-2.3,8.9-dibenzanthanthrene (**17**).

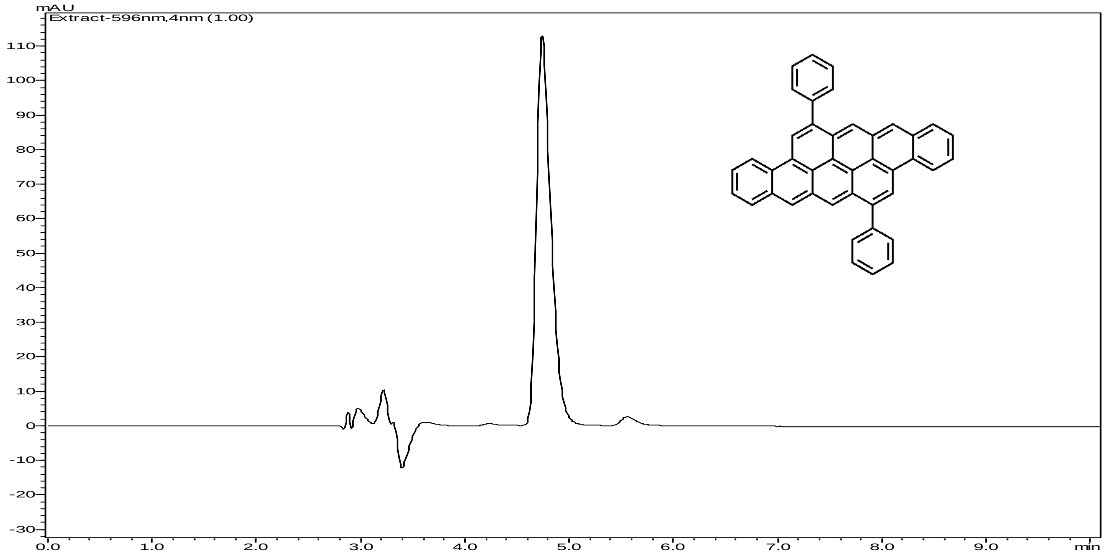

Supplementary Figure 80. HPLC profile of bisphenyl DBATT (**bPh-DBATT**) as obtained after *DPEX*. Detected at 598 nm (PBr column, toluene as eluent, 1 mL/min, 40ºC).

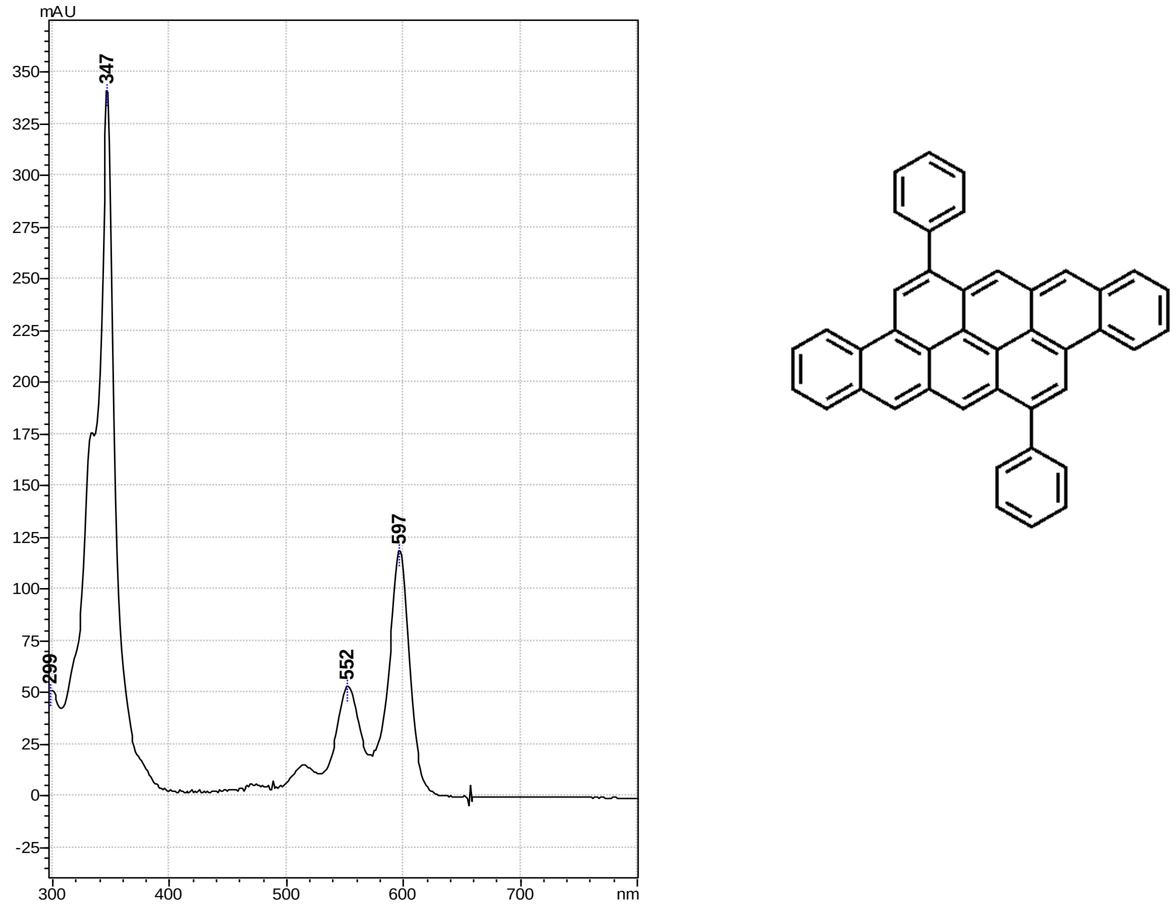

Supplementary Figure 81. UV-Vis spectrum of bisphenyl DBATT (**bPh-DBATT**) (toluene).

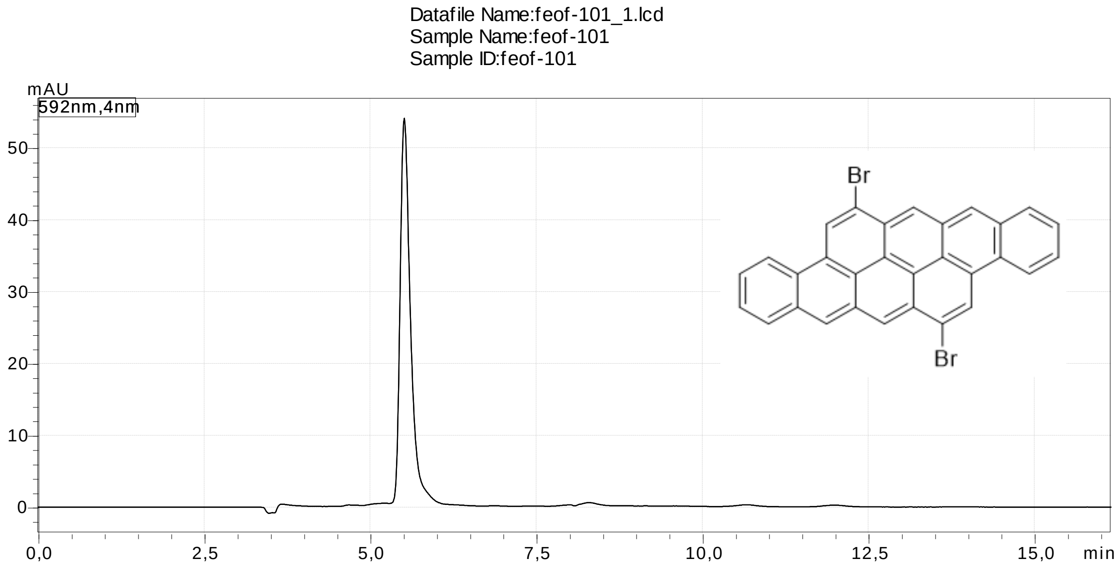

Supplementary Figure 82. HPLC profile of 6,14-dibromo-2.3,8.9-dibenzanthanthrene (**bBr-DBATT**) as obtained after *DPEX*. Detected at 592 nm (PBr column, toluene as eluent, 1 mL/min, 40ºC).

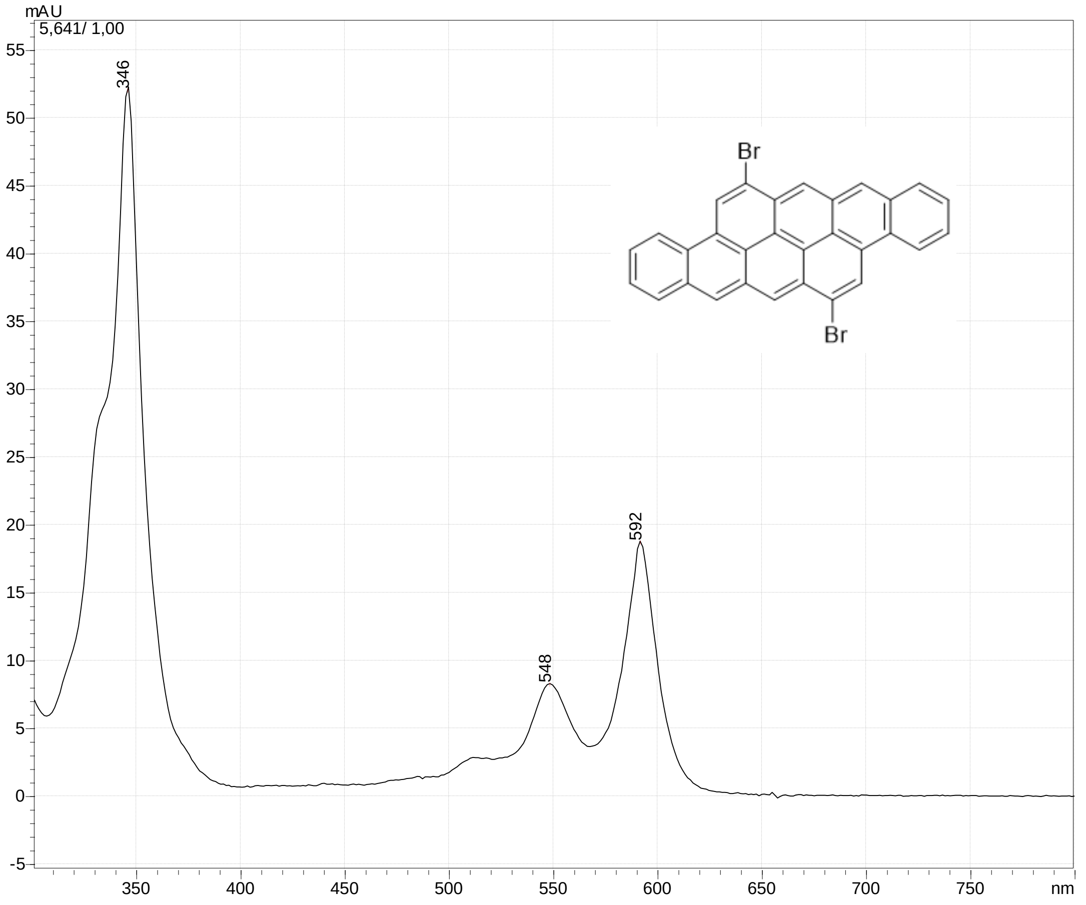

Supplementary Figure 83. UV-Vis spectrum of 6,14-dibromo-2.3,8.9-dibenzanthanthrene (**bBr-DBATT**) (toluene).

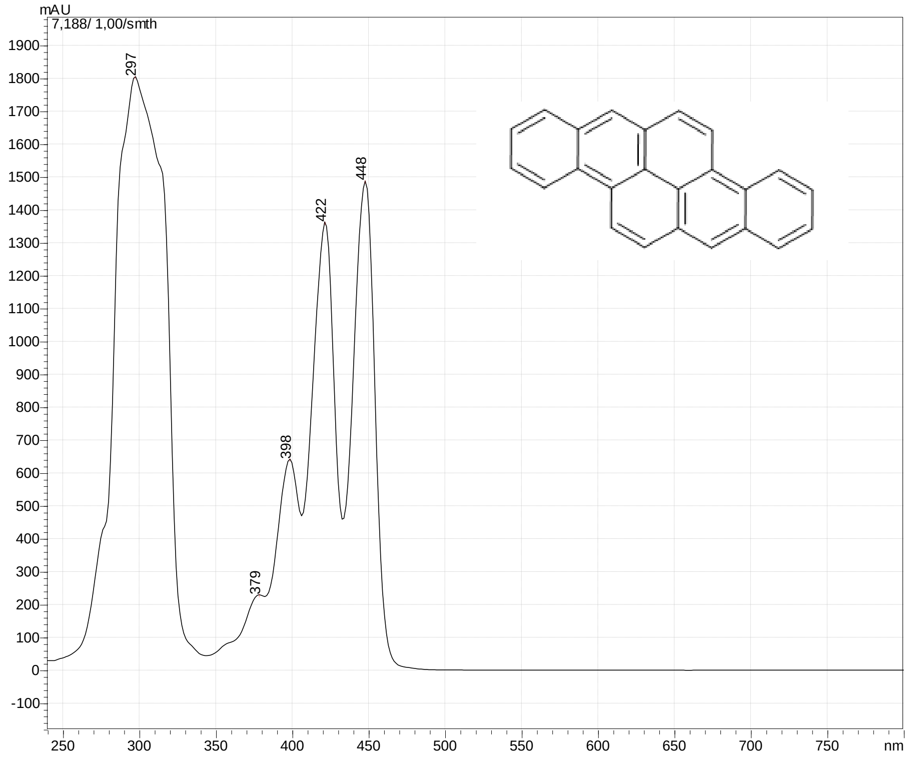

Supplementary Figure 84. UV-Vis spectrum of dibenzo[*b,def*]chrysene (**13**) (toluene).

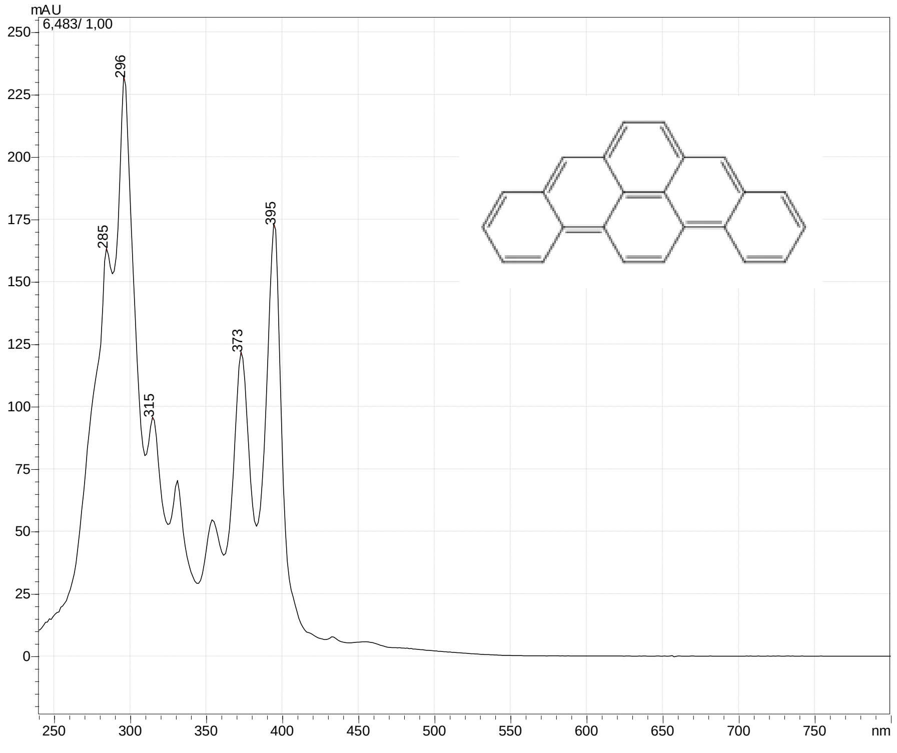

Supplementary Figure 85. UV-Vis spectrum of 6,14-dibromotetraceno[2,1,12,11-opqra]tetracene (**13**) (toluene).

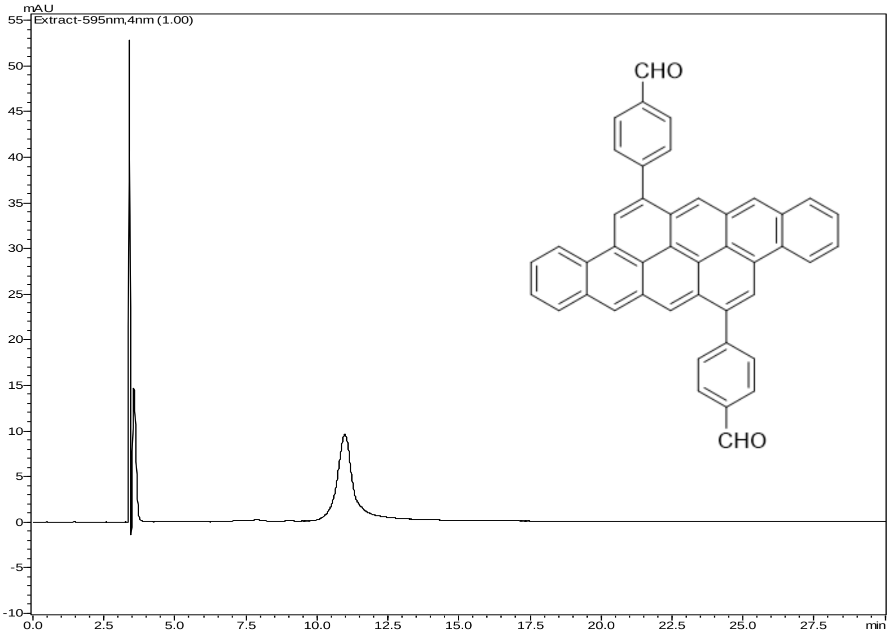

Supplementary Figure 86. HPLC profile of 6,14-bis(*para*-formylphenyl)-2.3,8.9-dibenzanthanthrene (**17**) after aqueous work-up of *DPEX* reaction; detected at 595 nm (PBr column, toluene-MeOH 1-1 as eluent, 1 mL/min, 40ºC).

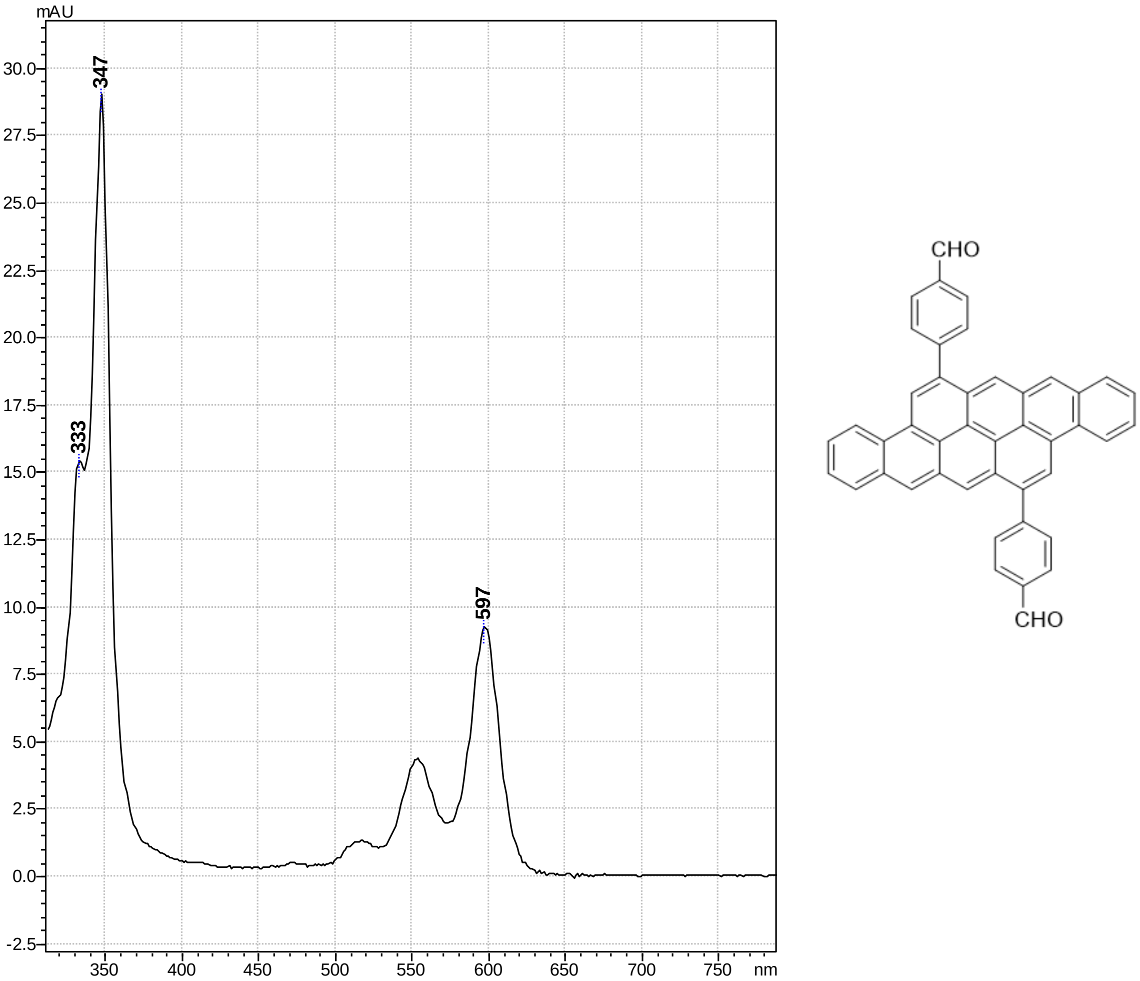

Supplementary Figure 87. UV-Vis spectrum 6,14-bis(*para*-formylphenyl)-2.3,8.9-dibenzanthanthrene (**17**) (toluene/MeOH 1/1).

Supplementary References

1. Gaussian 09, Revision A.02, Frisch, M. J., et al. Gaussian, Inc., Wallingford CT, 2016.

2. Horcas, I., et al. WSxM: A software for scanning probe microscopy and a tool for nanotechnology. *Rev. Sci. Instr.* **78**, 013705 (2007).

3. Barth, J.V., Brune, H., Ertl G. & Behm, R.J. Scanning tunneling microscopy observations on the reconstructed Au(111) surface: Atomic structure, long-range superstructure, rotational domains, and surface defects. *Phys. Rev. B* **42**, 9307-9318 (1990).

4. Torrente, I.F., Franke, K.J. & Pascual, J.I. Structure and electronic configuration of tetracyanoquinodimethane layers on a Au(111) surface. *Int. J. Mass Spectrom.* **277**, 269-273 (2008).

5. Yang, Z., et al. Two-dimensional delocalized states in organometallic bis-acetylide networks on Ag(111). *Nanoscale* **10**, 3769-3776 (2018).

6. Endo, O., Kondoh, H. & Ohta, T. Scanning tunneling microscope study of bromine adsorbed on the Ag(111) surface. *Surf. Sci.* **441**, L924-L930 (1999).

7. Jiang L, et al. Low-temperature, bottom-up synthesis of graphene via a radical-coupling reaction. *J. Am. Chem. Soc*. **135**, 9050-9054 (2013).

8. Kamada K, et al., Singlet diradical character from experiment *J. Phys. Chem. Lett.* **1**, 937–940 (2010).

9. Dral, P.O. & Clark, T. Semiempirical UNO–CAS and UNO–CI: method and applications in nanoelectronics, *J. Phys. Chem. A* **115**, 11303–11312 (2011).
